# Conservation and divergence of protein pathways in the vertebrate heart

**DOI:** 10.1371/journal.pbio.3000437

**Published:** 2019-09-06

**Authors:** Joel D. Federspiel, Panna Tandon, Caralynn M. Wilczewski, Lauren Wasson, Laura E. Herring, Samvida S. Venkatesh, Ileana M. Cristea, Frank L. Conlon

**Affiliations:** 1 Princeton University, Princeton, New Jersey, United States of America; 2 University of North Carolina at Chapel Hill, Chapel Hill, North Carolina, United States of America; 3 Department of Genetics, Harvard Medical School, Boston, Massachusetts, United States of America; University of Colorado Denver - Anschutz Medical Campus, UNITED STATES

## Abstract

Heart disease is the leading cause of death in the western world. Attaining a mechanistic understanding of human heart development and homeostasis and the molecular basis of associated disease states relies on the use of animal models. Here, we present the cardiac proteomes of 4 model vertebrates with dual circulatory systems: the pig (*Sus scrofa*), the mouse (*Mus musculus*), and 2 frogs (*Xenopus laevis* and *Xenopus tropicalis*). Determination of which proteins and protein pathways are conserved and which have diverged within these species will aid in our ability to choose the appropriate models for determining protein function and to model human disease. We uncover mammalian- and amphibian-specific, as well as species-specific, enriched proteins and protein pathways. Among these, we find and validate an enrichment in cell-cycle–associated proteins within *Xenopus laevis*. To further investigate functional units within cardiac proteomes, we develop a computational approach to profile the abundance of protein complexes across species. Finally, we demonstrate the utility of these data sets for predicting appropriate model systems for studying given cardiac conditions by testing the role of Kielin/chordin-like protein (Kcp), a protein found as enriched in frog hearts compared to mammals. We establish that germ-line mutations in Kcp in *Xenopus* lead to valve defects and, ultimately, cardiac failure and death. Thus, integrating these findings with data on proteins responsible for cardiac disease should lead to the development of refined, species-specific models for protein function and disease states.

## Abstract 2

Comparison of cardiac proteomes across four vertebrate model systems reveals species-specific differentially enriched proteins and pathways, including the Xenopus-enriched Kielin/chordin-like protein (Kcp), which is shown to be important for proper heart development.

## Introduction

Heart disease remains the most common cause of death in the western world [1]; however, comparatively little is known about the proteins and pathways that lead to formation of a human heart or how disruption of these pathways lead to the pathologies of cardiac disease. To determine the function of cardiac proteins and to aid the development of diagnostics and treatments, an array of model systems have been used [2–6]. To date, such studies have relied predominately on the mouse. However, this system has limitations, including the difficulty to perform live imaging over wide developmental time windows. Moreover, rodent anatomy is not always reflective of a human heart. For example, the mouse resting heart rate is 600 to 800 beats per minute, implying that its conduction system is not similar to that of a human [7]. The pig heart has many anatomical and physiological properties with a higher resemblance to the human heart, yet it remains a poor model for a subset of human cardiac disease states [8–10] and is associated with high cost.

Because of these limitations, there has been an effort to test gene function from large data sets in a nonmammalian system in a high-throughput fashion [11]. *Xenopus* allows in a single organism integration of systems-level genomic and proteomic analyses with quantitative live imaging of cardiac cell behaviors in an externally developing embryo. Moreover, many of the developmental processes are conserved between *Xenopus* and human [12–16]. Indeed, *Xenopus* has proven to be a valuable tool for defining human cardiac mutations [16–24], as well as for modeling human heart disease [25–32].

Because there are fundamental anatomical and physiological differences between frogs, rodents, and humans in cardiac development [33–37], it is critical to determine which proteins and protein pathways are conserved and which have diverged between *Xenopus*, mouse, and pigs. To address these issues and provide insights into which model system is better suited for investigating cardiac protein function and different cardiac conditions, we have conducted a comparative proteomic analysis of cardiac tissue in these major heart disease model systems. Specifically, we compared the cardiac proteomes of *Mus musculus*, *Sus scrofa*, and, to determine the conservation between mammals and *Xenopus* and the divergence in a single genus, *Xenopus laevis* and *X*. *tropicalis*.

Here, we report a conserved set of proteins, protein complexes, and protein pathways that correlate with a common set of cardiac metabolic functions in species with a separate pulmonary and systemic circulatory system. We further uncover mammalian- and amphibian-specific, as well as species-specific, enriched proteins and protein pathways. To test the utility of these data sets for predicting an appropriate model system to study a given cardiac condition, we examined the requirement for Kielin/chordin-like protein (Kcp) in the *Xenopus* heart. In our proteome data set, KCP was found to be enriched in *Xenopus* hearts when compared to mouse and pig hearts. Therefore, we chose to study Kcp because it represents a promising approach to modeling protein function in the human heart while bypassing some of the above-stated limitations of mouse and pig models. Over the past 20 years, mutations of proteins expressed in human, mouse, and *Xenopus* hearts have shown a similar phenotype, such as the Holt Oram disease/ T-box transcription factor 5 (TBX5)- [26–28, 38] and Tbx20-related congenital heart disease [29–32]. Although our study reveals candidate proteins from all 4 models that may be causative to human disease, the proteins known to be expressed in human and found in our study as enriched in frog, but not pig and mouse, provide promising insights, given the interest in alternative models. To investigate the role of Kcp, we have generated a germline null mutation of Kcp in *X*. *laevis* and performed a series of functional assays. Our results demonstrate that *Xenopus* has an essential requirement for Kcp in heart function. Altogether, our study provides a resource for cardiac proteomes in 4 major model systems, uncovering conserved and divergent protein pathways and providing insight into selection of appropriate model systems for either modeling cardiac development or investigating disease.

## Results

### Proteome characterization of 4 model organisms identifies a core cardiac program

**Fig 1:**
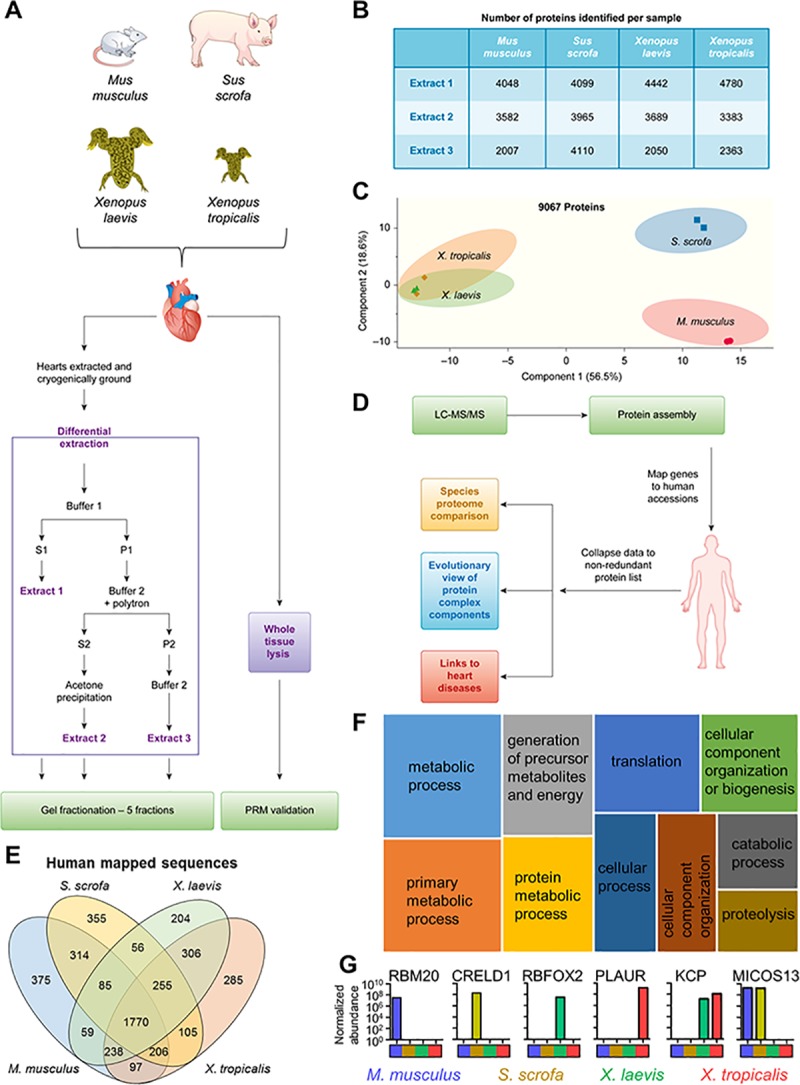
Workflow for investigating multispecies cardiac proteomes. (A) Heart tissue from *M*. *musculus*, *S*. *scrofa*, *X*. *laevis*, and *X*. *tropicalis* was collected and subjected to differential protein extraction as detailed. The resulting 3 extracts were fractionated by SDS-PAGE. (B) Number of proteins identified in the 5 gel fractions for each extract (extract 1–3 from panel A) per species. These numbers include proteins found in 2 or more extracts. (C) PCA of MS1 proteome data demonstrates that the mammalian samples separate from each other and the *Xenopus* samples but the *Xenopus* samples maintain a tight association. (D) Data analysis workflow. (E) Identified proteins were mapped to human accession numbers using Blast2GO. (F) The shared proteins in all species represent a core cardiac proteome that is enriched for a variety of pathways. Shown here in a tree map are the top 10 most enriched (adjusted *p* ≤ 0.05) GO biological process terms; box size scales with enrichment significance of the terms. (G) Examples of proteins detected in subsets of the species analyzed. See S8 Table for numerical data underlying figure. GO, Gene Ontology; Kcp, Kielin/chordin-like protein; LC-MS/MS, liquid chromatography coupled with tandem mass spectrometry;MS1, precursor ion.

To define those proteins and protein pathways that are conserved or diverged between *M*. *musculus*, *S*. *scrofa*, *X*. *laevis*, and *X*. *tropicalis*, we performed a differential protein extraction from whole aged-matched female heart tissues (Fig 1A and 1B). The resulting fractions were analyzed by liquid chromatography coupled with tandem mass spectrometry (LC-MS/MS) in biological replicates. To address the variation in sequence coverage across species in the databases, we generated and used for data analysis a combined FASTA file, containing sequences from all 4 species plus human (S1A Fig and S1 Table). For this analysis, we chose to examine canonical protein sequences only and not consider isoforms, because the detection of isoform-specific peptides across species may not be as robust as peptides present in all isoforms of a protein. Our analyses showed that the combined FASTA search strategy helped to increase the overall coverage of the proteome for each species by compensating for the sparser databases for some species (i.e., pig and *X*. *laevis*) and was superior to a search using single-species FASTA files for each organism (S1B and S2 Figs and S2 Table). Furthermore, each extract in our differential extraction protocol gave access to a unique pool of proteins (in addition to the overlapping identifications between the extracts; Fig 1B and S3 Fig). A higher number of identified proteins were present in extract 3 of the pig compared to the other species, indicating that the differential extraction protocol employed was less orthogonal for this species. We examined the properties of the proteins isolated by each extraction method by combining any protein detected in each extract across species and performed an enrichment analysis using Gene Ontology (GO) cellular component (S4 Fig). We found that most protein classes had members detected in all 3 extracts, with some that were detected in only 1 extract or a subset of extracts. For example, many nuclear proteins were detected in extracts 2 and 3, whereas endosomal proteins were detected in extracts 1 and 2. We also noted the presence of several ion channels that were detected in extracts 1 and 2 (S3 Table and S4 Table).

To reduce redundant assignments of the same protein to multiple species, the 15,382 identified proteins were assembled and clustered in Scaffold. In total, 9,067 protein clusters were observed, with approximately 4,500 proteins reproducibly identified for each species (Fig 1B, S5 Fig and S5 Table). This depth is in line with other previous studies of cardiac proteomes [39–41].

Quantification was performed using summed abundance-based precursor data for all detected peptides with median normalization (S6 Table). We next considered the possibility that our quantitation could be biased if protein ortholog length differed across species. Indeed, we observed a significant difference in the overall number of theoretical tryptic peptides across the species for the total proteomes (S6A Fig and S7 Table). However, for the proteins that were detected and quantified in our data sets, no significant difference in the theoretical number of tryptic peptides was observed between the species (S6B Fig). This allowed us to determine that we can use summed abundances for relative quantitation without length correction. Principal component analysis (PCA) of this quantitative data (Fig 1C) revealed that *M*. *musculus* and *S*. *scrofa* samples separated, whereas the 2 *Xenopus* species remained clustered, suggesting the presence of proteins that are commonly enriched in the amphibian species when compared to the mammalian species tested. Multiscatter plot analysis confirmed that the 2 *Xenopus* species had the highest degree of similarity (Pearson correlation = 0.83; S7 Fig). Strikingly, these proteome signatures were still observed when the PCA was conducted using the different extract samples (Fig 1A) as inputs instead of summed data from all extracts, indicating that the signature differences were captured in all individual fractions (S8 Fig).

To identify shared proteins and protein pathways, we removed remaining redundancies (e.g., where products of the same gene might exist in 2 or more clusters for the different species; Fig 1D and S9 Fig). This resulted in 4,710 quantifiable proteins (Fig 1E and S8 Table) that were used in all subsequent analyses, with 1,770 (38%) found in all species. This core cardiac proteome showed an enrichment of proteins (*p* ≤ 0.05) in pathways that include metabolism, translation, and cellular organization (Fig 1F and S10 Fig). A key component of cardiac proteomes is the myofilament subproteome [42], which is not shown as enriched in Fig 1F because of a lack of annotation in GO biological process, but which is present in our data set in the core proteome. We noted that approximately 70% of the myofilament subproteome, which included troponins C, T, and I, was detected across all species (S8 Table). By estimating the enrichment of the myofilament subproteome as a fraction of the cardiac proteome signal intensity for each species, we observed that these subproteomes corresponded to approximately 26% for the mammalian species and approximately 36% for the 2 *Xenopus* species. Also of note in our data set is that some proteins were detected in only one or a subset of species. For example, proteins such as RNA binding motif protein 20 (RBM20), Cysteine rich with EGF life domains 1 (CRELD1), RNA binding fox-1 homolog 2 (RBFOX2), and plasminogen activator, urokinase receptor (PLAUR) were only observed in one species, whereas others like KCP and MICOS complex subunit MIC13 (MICOS13) were detected in only 2 model organisms (Fig 1G). The approximately 10% higher contribution of the myofilament subproteome to the cardiac proteome of the *Xenopus* species further highlights proteins found to be enriched in these species (e.g., RBFOX2, PLAUR, and KCP). Altogether, our results suggest that abundance differences in cardiac proteins exist between these 4 model systems.

### Species-specific abundance profiles of cardiac protein complexes

**Fig 2:**
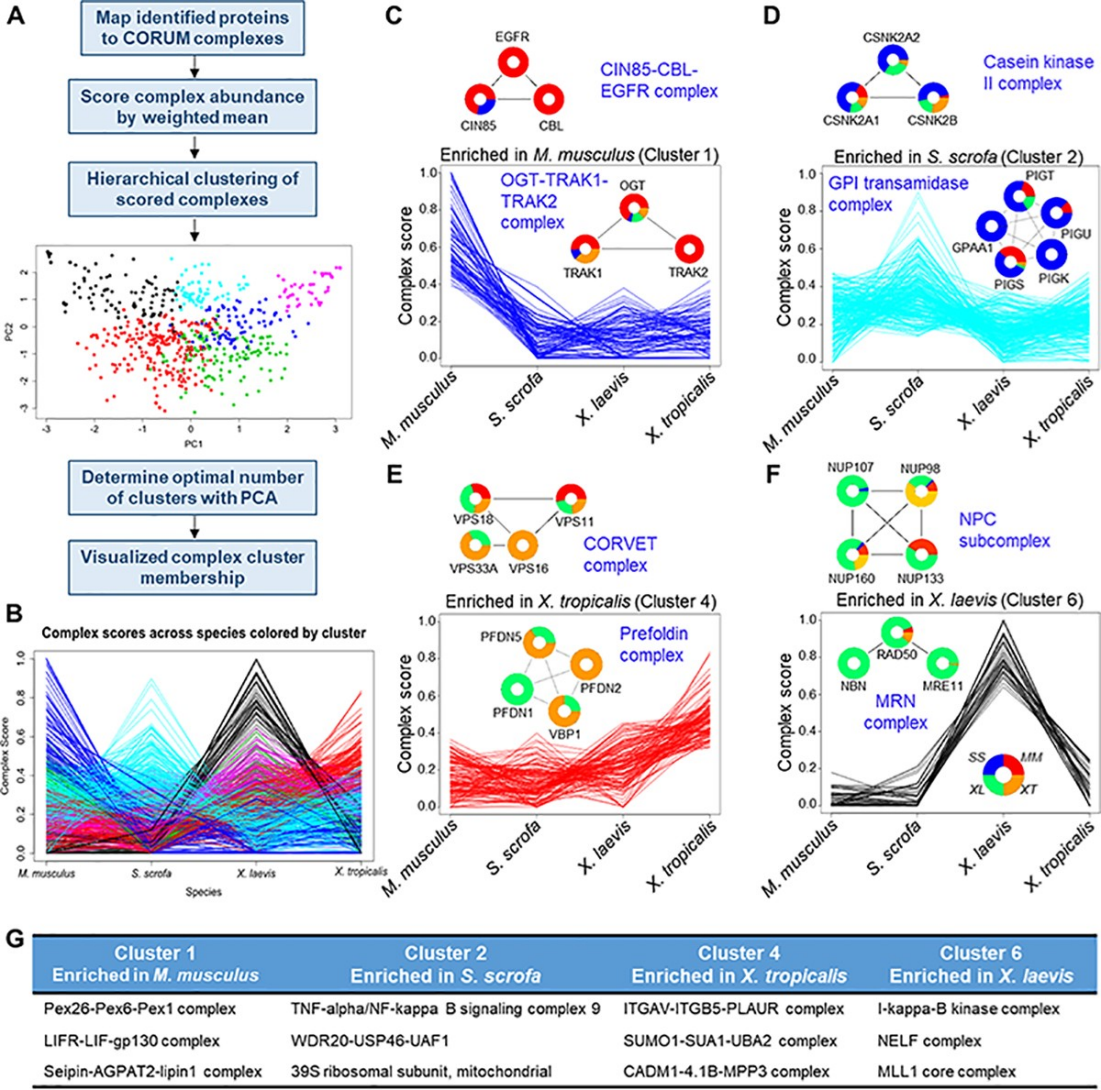
Evolutionary comparison of protein complexes. (A) Proteins detected in this study were mapped to known human protein complexes listed in CORUM. (B) A complex score was calculated from the MS1 peak area of individual protein components of each complex. These complexes were then clustered into 6 clusters and analyzed further. (C–F) Individual clusters are plotted along with example protein complexes, demonstrating the underlying abundance data that drove the complex clustering. (G) Three representative complexes for each cluster are listed. See S9 Table for numerical data underlying figure. CORUM, Comprehensive Resource of Mammalian Protein Complexes.

The assembly of proteins into macromolecular complexes is known to represent functional units within the proteome and to facilitate numerous biological processes, including those essential for cardiac function [43–46]. To expand our investigation of the heart proteome and begin to address the potential of differential abundances in cardiac proteins across species, we next aimed to predict which cardiac protein complexes are shared or unique between these model species. To accomplish this, we designed a computational approach (Fig 2A and S1 Code) that allowed us to use our quantitative proteome data to infer the presence and relative abundance of protein complexes in each species. To ensure robust and conservative complex monitoring, stepwise filtering criteria were used (S11 Fig). First, identified proteins were mapped to human complexes in Comprehensive Resource of Mammalian Protein Complexes (CORUM) [47], which we filtered to retain those complexes with 3 or more members. To further increase our stringency, while still allowing for the potential identification of less conserved complexes, we utilized a bipartite identification threshold. We removed any complexes consisting of 5 member proteins or less in which we had identified less than 40% of the complex components in one species. For complexes with 6 or more member proteins, the detection of 20% of the complex components in at least one species was required for the complex to be considered present. Using this filtered set of 794 complexes, we assigned an abundance score for each protein complex. Hierarchical clustering of the resultant scores revealed 6 clusters (Fig 2B), which were empirically determined using PCA and the cluster dendrogram. Examples of top-scoring protein complexes are shown for each cluster, with the corresponding cross-species MS quantification (Fig 2C–2F and S12A and S12B Fig). The 3 top-scoring complexes in each cluster are listed (Fig 2G and S12C Fig), and scores for all complexes are listed in S9 Table.

Functional enrichments within each cluster were assessed by overrepresentation analysis against the CORUM database (adjusted *p* ≤ 0.05). Cluster 1 (Fig 2C) contained numerous protein complexes related to immune response that were detected almost uniquely in *M*. *musculus*. Cluster 2 (Fig 2D) had several complexes involved in mitochondrial translation and metabolism, whereas cluster 4 (Fig 2E) was driven by protein complexes with roles in cell–cell contact and extracellular matrix organization. Cluster 6 (Fig 2F) was largely driven by protein complexes enriched in *X*. *laevis*, including cell-cycle–associated and DNA repair complexes. Clusters 3 and 5 (S12 Fig) contained complexes involved in mRNA splicing and receptor signaling. Perhaps surprising was the apparent lack of a cluster of complexes that displayed relatively similar abundances across species. To investigate this further, we filtered for those complexes that had less than 20% variance in their abundances across species, regardless of cluster assignment. This resulted in a subset of 24 complexes, largely derived from cluster 2. We examined the functional role of these protein complexes and found them to be largely involved in protein ubiquitination, autophagy, and RNA transport (S13 Fig).

Although analysis of CORUM complexes is a useful and informative way to examine protein complex conservation among these species, we additionally examined a cardiac-specific protein–protein interaction database to increase our protein complex coverage and potentially pick up cardiac-specific protein interactions. We chose to use the BioSNAP database [48], which is based on experimental evidence of physical protein–protein interactions, for this analysis and extracted all cardiac binary interactions from it. We then expanded these into the maximal possible complexes of at least 3 proteins in size based on the reported binary associations. Our own proteome data were mapped to this in the same way as CORUM, and the resulting complexes were hierarchically clustered into 8 clusters based on the dendrogram (S14A Fig, S10 Table, and S2 Code). We again detected clusters of proteins that were enriched in each species as well as those that were more evenly spread across the 4 species. Many of the same GO terms as in the CORUM analysis were also enriched in this BioSNAP clustering. For example, the *X*. *laevis*–enriched cluster (cluster 7; S14A Fig) was annotated for DNA repair proteins as was seen in the CORUM analysis. Both the CORUM and BioSNAP analyses provided complementary information as each database had uniquely mapped complex members from our data set (S14B Fig). For example, both databases provided insight into different proteins involved in mRNA splicing and translation, whereas BioSnap was more enriched for mitochondrial-organization–associated complexes and CORUM was more enriched for some metabolic complexes. Altogether, our results highlight the existence of differential abundance profiles for protein complexes within these cardiac model systems, which may drive some of the different observed phenotypes in these species.

### Mammalian and *Xenopus* cardiac pathway analysis reinforces concept of diverged proteomes

Although the core cardiac proteome identified in this study represents a set of conserved proteins and pathways, our protein complex analysis showed a surprising level of species-specific variation in complex abundances. To further investigate this, we used our quantitative MS data to examine potential abundance differences between species outside of the context of protein complexes for enrichments related to cardiac functions. We assessed the relative abundance of each protein shared across all 4 species and clustered these abundance profiles using k means (S15A Fig). The cluster showing commonalities among mammalian proteomes (cluster C) was enriched for proteins involved in metabolic processes related to lipid and carbohydrate metabolism, whereas the *Xenopus* proteomes (cluster D) exhibited enrichments in cell-cycle–associated proteins, intracellular signal transduction, and cell communication (S15B Fig and S11 Table). Also evident were clusters of species-enriched proteins, complexes, and pathways. For example, the *M*. *musculus*–driven cluster (A) was enriched in vesicle-mediated transport functions, *S*. *scrofa* (B) was enriched in mitochondrial and transcriptional categories, *X*. *tropicalis* (F) showed enrichment in RNA processing proteins, whereas *X*. *laevis* (E) had enrichment in cell adhesion proteins (S15B Fig). These results of differential protein abundances among commonly detected proteins in the 4 species serve to further emphasize the variation in the cardiac proteomes for these model systems.

We next examined proteins detected uniquely in either mammalian or amphibian samples (S16 Fig and S12 Table). Using ClueGO, 63 terms were found as significantly enriched in either mammals or amphibians (adjusted *p* ≤ 0.01). In mammals, an increased number of metabolic and mitochondrial proteins was observed, possibly reflecting the increase in energy production required for 4-chambered hearts versus the 3-chambered heart of frogs.

### Cell-cycle enrichment in *Xenopus laevis* represents an example of a single genus cardiac proteome divergence

**Fig 3:**
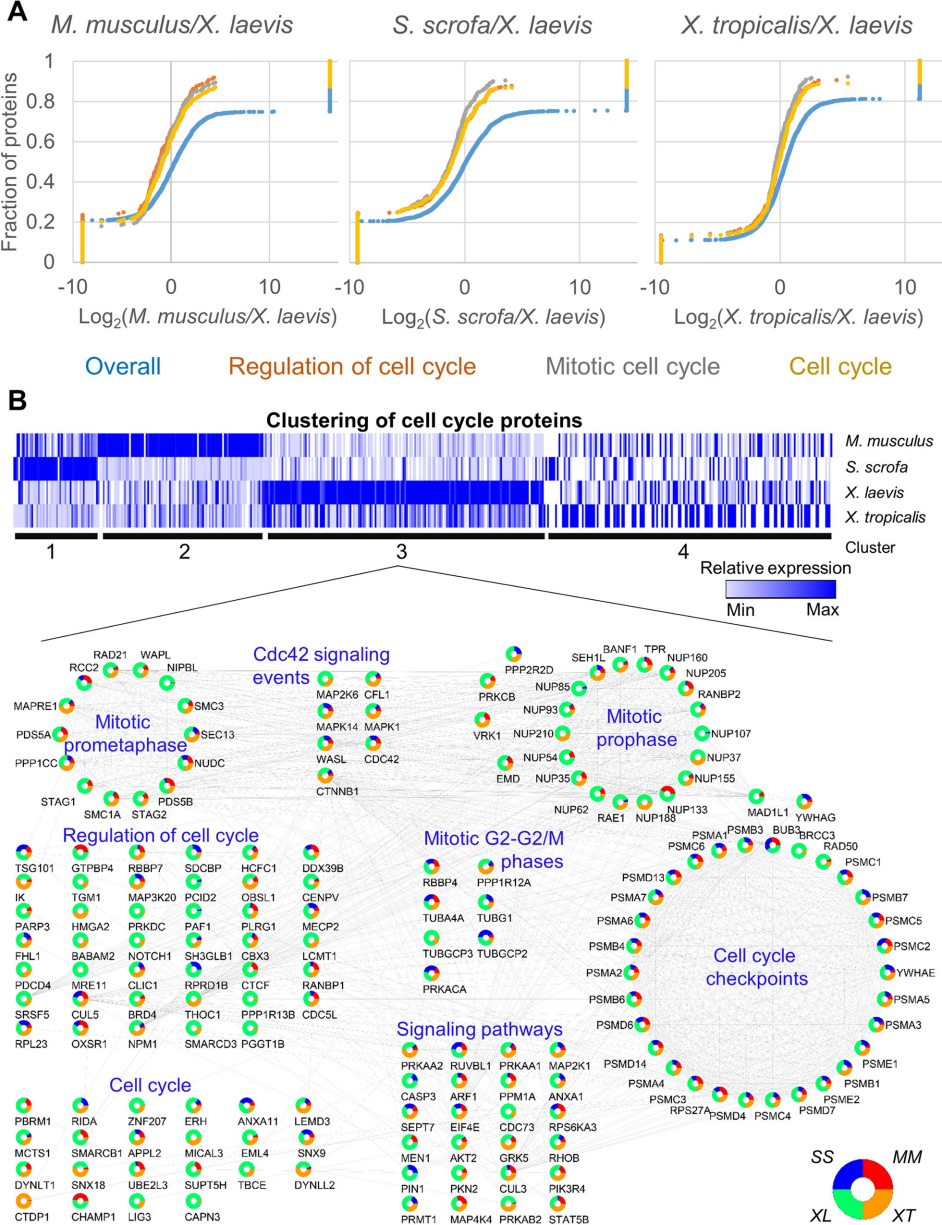
GSEA reveals the increased relative abundance in cell-cycle proteins in *Xenopus laevis*. (A) Pairwise GSEA was carried out between *X*. *laevis* and the 3 other species, revealing an enrichment (adjusted *p* ≤ 0.05) in cell cycle related proteins in *X*. *laevis*. (B) All proteins found in any enriched cell cycle related category by GSEA were clustered using k means (k = 4). Relative protein abundance is shown. The proteins found in cluster 3 were further analyzed in Cytoscape and grouped by function to show the interrelated nature of the proteins enriched in this cluster. See S13 Table and S14 Table for numerical data underlying figure. GSEA, Gene Set Enrichment Analyses.

An unexpected finding from our analyses was the divergence between the 2 related frog species, *X*. *tropicalis* and *X*. *laevis*. We investigated this further by conducting pairwise Gene Set Enrichment Analyses (GSEA) of *X*. *laevis* with all other species (S13 Table and S14 Table). Our analysis confirmed there were indeed differences between the 2 frogs, with one of the most striking changes being a significant enrichment in cell-cycle proteins in *X*. *laevis*. This set of proteins includes 74 that are at least 1.5-fold enriched in abundance in *X*. *laevis* compared with *X*. *tropicalis*, as well as other proteins with smaller abundance differences. The proteins responsible for this enrichment fell into 11 different GO categories (Fig 3A and S17 Fig) and were found to be in a diverse set of cell-cycle pathways, including cell-cycle checkpoints, G2 and M transition, cell-cycle regulation, and cell-cycle signaling (Fig 3B). To account for a possible bias derived from gene duplications in the allotetraploid *Xenopus laevis*, we performed again the quantification by correcting for genes in cell cycle that are duplicated. This analysis provided further support for our original observation, because the enrichment of cell-cycle proteins was retained (S18 Fig and S15 Table). Further evidence for the specificity of this enrichment comes from our finding that this is not a global up-regulation of protein abundances in *X*. *laevis* when compared to the other species. For example, mitochondrial proteins were enriched in *S*. *scrofa* compared to the other species (S19 Fig). Thus, changes in cardiac protein abundance appear to be species specific and to have occurred within a single genus.

### Targeted mass spectrometry validation of cell-cycle protein enrichment in *X*. *laevis*

To further validate our discovery of a diverged set of cardiac protein abundances across species, we performed an orthogonal assay based on quantitative targeted mass spectrometry (MS). We focused this analysis on the enriched cell-cycle proteins in *X*. *laevis* versus *X*. *tropicalis*, *M*. *musculus*, and *S*. *scrofa*. This assay served not only as a specific validation of the enrichment in cell-cycle protein in *X*. *laevis* but also as a general validation of our proteomic discovery method. The discovery approach discussed above relied on quantifying proteins based on all detected peptides, some of which were unique to a certain species because of differences in protein sequences, and then mapping the proteins identified to human orthologs. Therefore, to further validate our findings, we used a targeted assay, based on parallel reaction monitoring (PRM)-MS that eliminates potential variabilities. For this, we had to first compile and experimentally validate a list of peptides that were specific to each protein but shared across all orthologs of the proteins in the organisms studied. This ensured that the exact same molecular species was being measured for all model systems, thereby providing a more stringent and accurate quantitative comparison.

**Fig 4:**
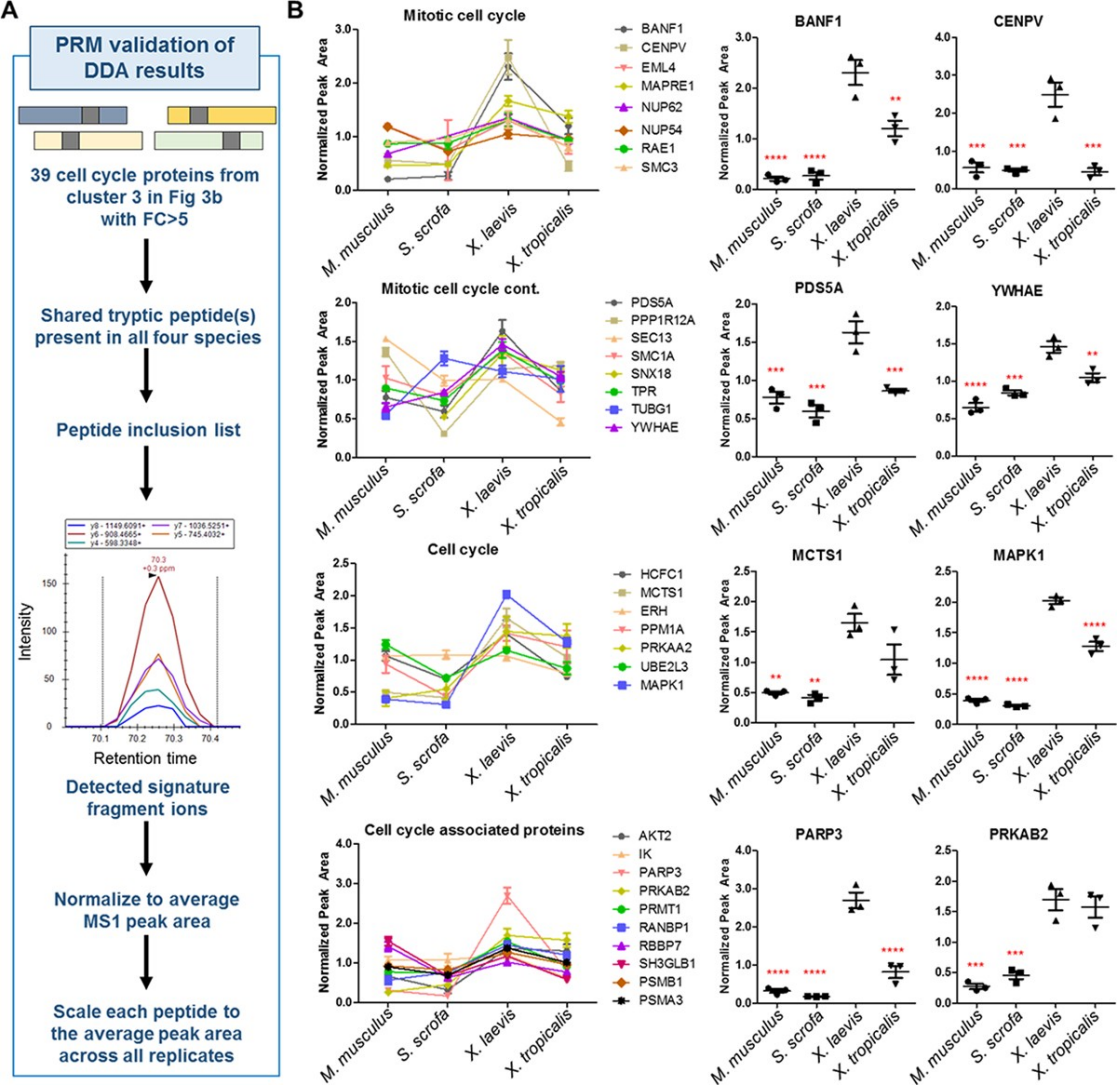
Validation of cell-cycle enrichment in *X*. *laevis* using targeted MS. (A) Workflow of target protein and peptide selection for PRM MS–based validation. (B) Mean protein abundance with SEM in each species by PRM assay is shown. The data were grouped by functional classes for visualization. For all proteins in (B), *Xenopus laevis* was significantly (*p* ≤ 0.05) higher than at least one other species. Shown to the right of each graph are examples of the most enriched proteins in each functional class (**p ≤ 0.01, ***p ≤ 0.001, ****p ≤ 0.0001). See S16 Table for numerical data underlying figure. DDA, data dependent analysis; MS, mass spectrometry; PRM, parallel reaction monitoring.

Our analysis focused on those cell-cycle proteins identified as enriched in *X*. *laevis* by at least 5-fold versus at least one other species (Fig 4A), from which we iteratively generated a final list of 39 high-confidence cell-cycle protein targets (S16 Table). The final PRM assays were run on an independent set of samples in 3 biological replicates, and loading was normalized based on the average abundance of all peptides present in the sample (i.e., average MS1 abundance). Resulting peak areas for each protein are illustrated as grouped by functional categories (Fig 4B). Quantification graphs with significance levels for individual proteins are illustrated in S20 Fig. This assay confirmed enrichment in *X*. *laevis* compared to at least one other species for 33 of the 39 proteins targeted (85%; Fig 4B). Examples of best-enriched proteins within each functional category are illustrated (Fig 4B, right).

We noted that, although almost all of the proteins targeted by PRM were found to be enriched in *X*. *laevis*, many of the fold changes were more muted than what was observed in the discovery data (Fig 3). However, several major differences between these experimental workflows have to be taken into account. A key point of the PRM assay was to target identical peptides in the homologous proteins from the different species, which necessarily limited the number of peptides that were useful for quantitation. In contrast, the discovery data used all the identified peptides for MS1 quantification, perhaps facilitating the larger observed fold changes. Additionally, we opted to perform the PRM assay without additional biochemical fractionation of the samples to limit possible biases derived from sequential sample handling steps. However, the lack of fractionation also results in increased sample complexity, which can impact peptide detection by MS. Altogether, given these major differences between workflows, the recapitulation of increased abundance of cell-cycle proteins in *X*. *laevis* provided confidence for this finding and served to further validate our discovery approach as a whole.

### Modeling of human cardiac disease states

**Fig 5:**
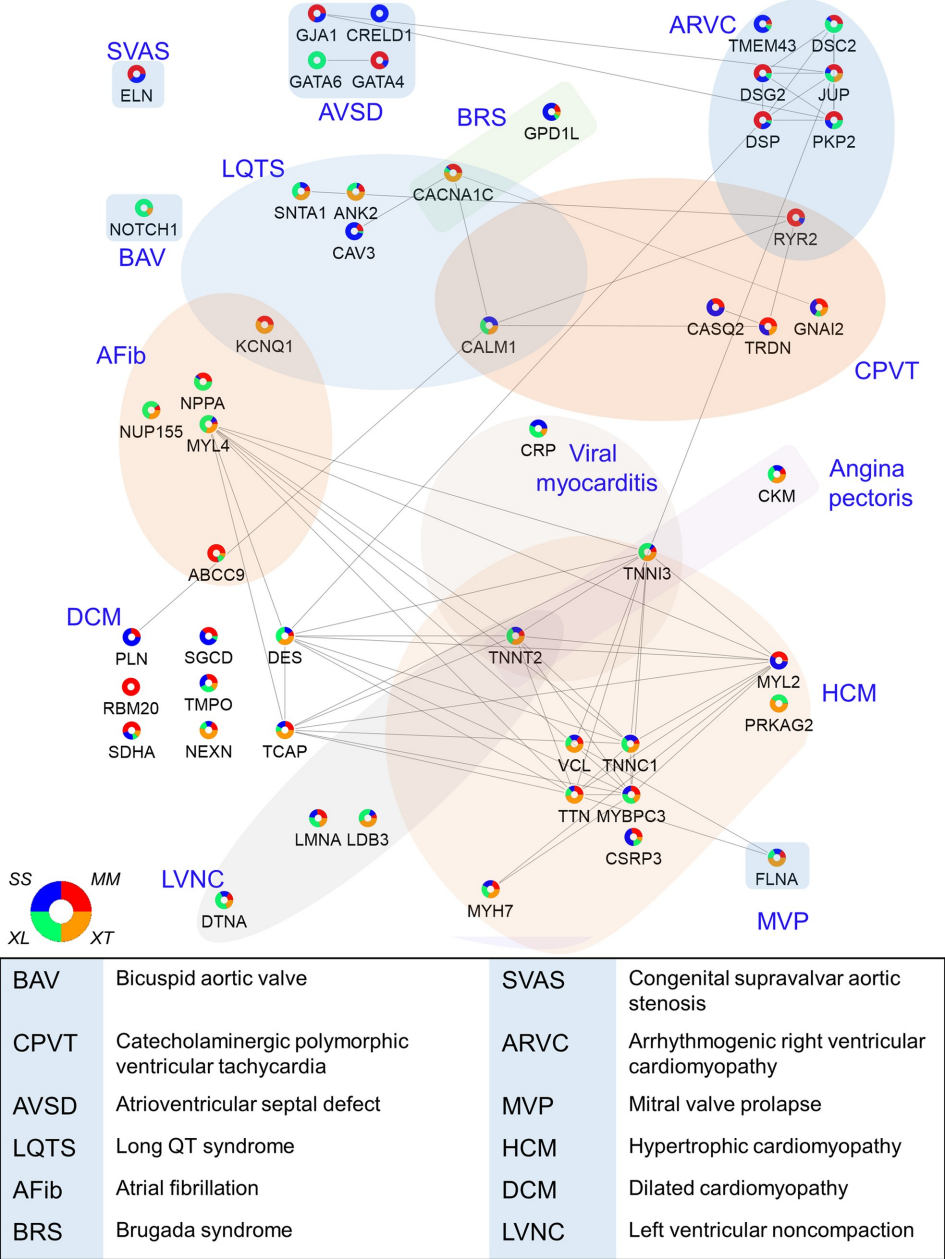
Association of cardiac protein abundance and model system for selected proteins with known roles in human cardiac disease. A diagram of detected proteins with links to human heart disease reported in the KEGG database was created in Cytoscape showing relative protein abundance data across the 4 species examined here, as well as known protein–protein interactions between these heart disease–related proteins. See S8 Table for numerical data underlying figure. AFib, atrial fibrillation; ARVC, arrhythmogenic right ventricular cardiomyopathy; AVSD, atrioventricular septal defect; BAV, bicuspid aortic valve; BRS, brugada syndrome; CPVT, catecholaminergic polymorphic ventricular tachycardia; DCM, dilated cardiomyopathy; HCM, hypertrophic cardiomyopathy; LQTS, long QT syndrome; LVNC, left ventricular noncompaction; MVP, mitral valve prolapse; SVAS, congenital supravalvar aortic stenosis.

In our discovery data, we noted that some proteins were detected in only 1 or 2 species but not found in all (e.g., Fig 1G). This observation of species-specific differential abundance profiles across proteins, protein complexes, and protein pathways led us to investigate the correlation between these proteins and those implicated in human cardiac disease (Fig 5). Surprisingly, we find that only some model organisms have enriched abundance for the proteins associated with any given human disease state. For example, dilated cardiomyopathy (DCM), which is defined by systolic dysfunction and dilation of one or both ventricles, can be caused in humans by mutations in the genes encoding cardiac phospholamban (PLN), delta-sarcoglycan (SGCD), and RNA-binding protein 20 (RBM20) [49, 50]. Interestingly, we detected RBM20 only in mouse, and PLN was detected in both pig and mouse, whereas SGCD was observed in pig, mouse, and at low levels in *X*. *laevis*. It is important to note that the absence of detection of a protein in a species does not mean that this particular species does not express this protein but rather that the protein abundance is more enriched in the species in which the protein was detected. Additionally, a lack of detection in a particular species can also occur in cases in which proteins have diverged across evolutionary space to the point that there is limited amino acid sequence homology. With careful consideration of these caveats, this type of analysis can point to pathways that may be up-regulated in a given species.

These findings of species-specific differential abundances were not unique to DCM but were observed in each of the human cardiac disease states examined. For example, we find a similar set of correlations for atrioventricular septal disease (AVSD) [51], which in human is caused by mutations in CRELD1 (enriched in pig), Gap junction alpha-1 (GJA1) and transcription factor GATA-4 (GATA4) (enriched in mouse and pig), and GATA6 (enriched in *X*. *laevis*). Together, these findings lead us to propose that the capacity of an animal model to accurately mimic the pathologies of a given heart disease may be directly related to the abundance level of those proteins in that species.

### KCP is essential in *X*. *laevis* for heart development and survival

**Fig 6:**
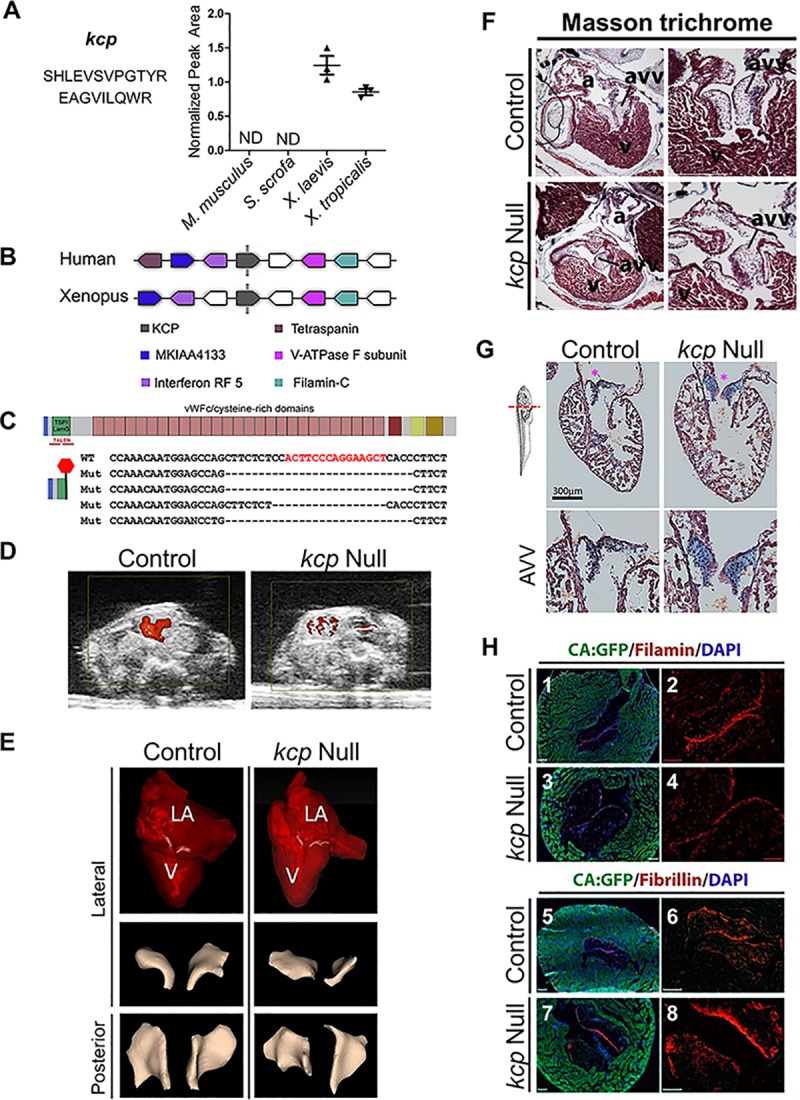
Kcp is essential for heart development and survival in *X*. *laevis*. (A) PRM quantification of peptides specific for Kcp confirms that Kcp is enriched in *Xenopus*. (B) Human and *Xenopus* show chromosomal synteny as revealed by Metazome. KCP is indicated in black. Upstream and downstream genes are colored as indicated. (C) Schematic of Kcp showing relative position of TALEN-induced mutations and bottom predicted protein generated by TALEN mutagenesis. (D) Stiol images of ultrasound Doppler of living wild type and Kcp^Δexon2/ Δexon2^ at Stage 64 positioned with dorsal top, ventral bottom of image. Blood flow shown in red. (E) Contrast-enhanced CT imaging of wild-type and Kcp^Δexon2/ Δexon2^ hearts (Stage 64) with AVV highlighted in white, bottom panels show lateral and posterior views showing thicker and misshapen AVV in Kcp^Δexon2/ Δexon2^ hearts verses control. (F) The AVV are detached from the muscular walls in Kcp mutants. Transverse sections through Masson trichrome stained Stage 64 control hearts (heterozygous) at low (upper left panel) and high (upper right panel) magnification focused on the AVV. Similar sections through a Kcp null froglet in at low (lower left panel) and high (lower right panel) magnification. Note in lower left panel an enlarged AVV in the outer valve leaflet detaching from the muscle wall. (G) Histology of wild-type and Kcp^Δexon2/ Δexon2^ hearts with Alcian Blue staining shows massive accumulation of collagen (blue) in AVV. a* Denotes position of AVV. (H) AVVs in kcp null hearts show a decrease in Filamin A and a concomitant increase in Fibrilin in the outer valve leaflet. (1–8) Immunochemistry of Cardiac-Actin:GFP (marking cardiomyocytes with GFP) froglets stained for Filamin (red), and DAPI (blue) in (1 and 2) heterozygous controls and (3 and 4) Kcp null. (5 and 6) Immunochemistry of Cardiac-Actin:GFP (marking cardiomyocytes with GFP) froglets stained for Fibrillin (red) and DAPI (blue) in (5 and 6) heterozygous controls and (7 and 8) Kcp null. See S16 Table for numerical data underlying figure. a, atria; avv, atrioventricular valve; GFP, green fluorescent protein; Kcp, Kielin/chordin-like protein; PRM, parallel reaction monitoring; TALEN, transcription activator-like effector nuclease; v, ventricle.

Thus far, studies have predominantly relied on mice to test the function of human cardiac proteins. Our findings imply not all human cardiac proteins are similarly expressed in mice, and therefore, the mouse may not be suitable for evaluating the function of each cardiac protein. One of the utilities of our data set is that it allows the choice of an animal model to determine the requirement for a human cardiac protein a priori. To test this, we focused on proteins known to be expressed in the hearts of humans and that our data demonstrated to be enriched in frogs but absent in mouse and pig. One such protein was Kcp. Kcp is a secreted protein composed of multiple cysteine-rich domains and expressed predominantly in the developing neural system, limb bud, and kidney during development [52–54]. Unlike other cysteine-rich proteins, such as Chordin, Kcp is unique in inhibiting transforming growth factor beta (TGFβ) signaling and can augment bone morphogenetic protein (BMP) signaling [52–54]. Our data set shows Kcp was detected only in *X*. *laevis and X*. *tropicalis* hearts, indicating that this protein is enriched in *Xenopus* when compared to mouse or pig (S8 Table and Fig 1G). Given the difference in the number of peptides identified in *X*. *tropicalis* and *X*. *laevis*, we sought to confirm this observation by performing a more accurate quantification using targeted MS. For this, we searched for tryptic peptide sequences that are conserved among these species. We observed that, although the sequence of the *Xenopus* Kcp is longer than the mammalian KCP, many residues are conserved across the *Xenopus* species, pig, mouse, and human (S21 Fig). Additionally, Kcp is annotated to contain several copies of Von Wiilebrand factor (VWF) type C domains and one VWF type D domain, most of which are also conserved across species. However, despite these sequence similarities, the 4 sequences examined here do not contain shared tryptic peptides. Therefore, we designed a targeted MS assay (using PRM) to monitor 2 peptides shared between *X*. *tropicalis* and *X*. *laevis*, as well as 4 tryptic peptides found to be shared between the mouse and pig species (S16 Table). We were able to readily detect the 2 shared peptides in both *Xenopus* species (Fig 6A). However, none of the targeted peptides were detectable in the mammalian species, further supporting the enrichment of Kcp in the *Xenopus* species. Our PRM results indicate similar levels between the 2 species of *Xenopus*. Therefore, *Xenopus* could provide a relevant model system for determining the requirement for Kcp in heart tissue.

To investigate the function of Kcp, we cloned *X*. *laevis* Kcp and found *X*. *laevis* to have a single pair of alleles of Kcp (Chromosome 3L: 77869526–77925178) that are in synteny with human Kcp (Fig 6B). In order to confirm the activity of *X*. *laevis* Kcp, we conducted bioassays testing its ability to augment BMP signaling. These assays are based on the observation that injection of BMPs into ventral blastomeres in *Xenopus* leads to a partial secondary axis formation [55] (S22A and S22B Fig). We find injecting low doses of either BMP2 mRNA alone (500 pg) or Kcp mRNA alone (500 pg) into ventral blastomeres of *X*. *laevis* leads to a low percent of anterior axis protrusions (12% and 0%, respectively), edema (7.5% and 6%, respectively), or anterior defects (2% and 2%, respectively). In contrast, we observe a synergistic effect by co-injection of BMP2 and Kcp, including anterior axis protrusions (25%), edema (21%), anterior defects (32%), and a unique class of posterior defects (19%; S22C–S22E Fig and S17 Table). Altogether, these data demonstrate *X*. *laevis* Kcp acts to augment BMP2 signaling.

To determine the endogenous function of *X*. *laevis* Kcp, we utilized transcription activator-like effector nuclease (TALEN) technology to introduce mutations into coding exon 2 of the Kcp locus by nonhomologous end joining (Fig 6C). Embryos injected with Kcp TALENs were grown until adulthood, whereby founder F0 animals were mated to produce mutant Kcp offspring. Sequencing showed that the mutations consistently result in the introduction of a premature stop codon in exon 2, thereby truncating the protein prior to the cysteine-rich domains that have been shown to be essential for BMP protein modulators such as Chordin (Fig 6C) [52–54]. Frogs heterozygous for a 29 bp deletion in Kcp (Kcp^Δexon2/+^) were viable and fertile and displayed no obvious phenotypic abnormalities. By contrast, in timed heterozygous matings, no froglets homozygous for KCP^Δexon2/ Δexon2^ were recovered after Stage 66, and we found a marked lethality of KCP^Δexon2/ Δexon2^ froglets between Stages 62 to 66 in 4 independent matings (Fig 6D–6G).

The timing of lethality is of particular interest as it represents the time when both pulmonary and systemic cardiac circulations are required to support the air-breathing adaptations of the froglet. To determine the morphology of the cardiac defects at this period, we conducted live ultrasound coupled with Doppler power imaging on control and Kcp^Δexon2/ Δexon2^ froglets. Results show a significant decrease in blood flow in Kcp^Δexon2/ Δexon2^ by Stage 64, indicative of poor cardiac function and thus the likely cause of death (Fig 6D and S1–S4 Movies). Contrast-enhanced CT imaging additionally showed Kcp^Δexon2/ Δexon2^ to have atrioventricular valves (AVVs) that were thicker and misshapen in the outer parietal valve leaflet relative to controls (Fig 6E and S5 and S6 Movies).

Based on these finding, we hypothesized that Kcp^Δexon2/ Δexon2^ animals have AVV myxomatous valve degeneration. In humans, this disease is associated with reduced cellular composition in the AVV and detachment from the muscular walls in association with an increase in collagen deposition in the outer leaflet. To test if Kcp null froglets were dying from AVV myxomatous valve degeneration, we performed histological analysis to look at collagen deposition using Alcian Blue staining. When compared to control animals, we find Kcp^Δexon2/Δexon2^ animals to have a detached AVV (Fig 6F) and a marked increase in collagen along the medial and lateral edges of the AVV (Fig 6G). To confirm the AVV myxomatous valve degeneration phenotype, we further assayed for hallmarks of this disease analyzing the expression of fibrillin and filamin [56] in control animals and Kcp mutants. Consistent with the Kcp null froglets having AVV myxomatous valve degeneration, we observe that Kcp^Δexon2/ Δexon2^–derived hearts have a marked decrease in the outer leaflet in filamin A with a concomitant increase in fibrillin (Fig 6H). Taken together, these findings demonstrate that there is an essential requirement for Kcp in *X*. *laevis* valve development and further suggest that *X*. *laevis* might be a suitable model system to study the role of Kcp in heart disease.

## Discussion

The data set described here allowed for comparison of the heart proteomes of *X*. *laevis*, *X*. *tropicalis*, *M*. *musculus*, *and S*. *scrofa* and used several different bioinformatic approaches to gain insight into the proteomic data. The different species’ data were mapped to orthologous human proteins to allow direct comparison among the species and incorporation of known human data. For proteins of interest that displayed species enrichment, we validated the quantitative data obtained from this methodology by performing a targeted MS assay that quantified only those peptides fully shared between the 4 species. These findings expand on the growing interest in understanding the cardiac proteome, including recent studies in the human heart [57, 58], in mouse models of mitochondrial heteroplasmy [59], and aorta atherosclerotic plaque formation [60].

Our comparative analysis between *X*. *laevis*, *X*. *tropicalis*, *M*. *musculus*, and *S*. *scrofa* cardiac proteomes showed that species with a pulmonary and systemic circulatory system contain a conserved set of cardiac proteins, complexes, and pathways that correlate with a common core set of metabolic functions. Of the pathways shared by *X*. *laevis*, *X*. *tropicalis*, *M*. *musculus*, and *S*. *scrofa*, metabolism and mitochondrial associated proteins represented the most significantly enriched protein classes, reflecting the universal importance of energy generation in heart tissue. Proper expression and localization of proteins is also of importance in all species examined, as evidenced by enrichments in translation, nuclear transport, protein folding, and localization.

Given the high degree of similarities in the genomic sequence and given the highly conserved anatomy, physiology, and molecular underpinnings of heart development between *X*. *tropicalis* and *X*. *Laevis* [19, 24, 34], we surprisingly find a species-specific divergence of protein abundance profiles in the heart. Interestingly, this has occurred even within a single genus, with a dramatic increase in protein abundance observed in the cell cycle machinery of *X*. *laevis* compared with that of *X*. *tropicalis*. *X*. *laevis* is an allotetraploid frog possessing 2 diploid genomes, the L and S genomes, derived from the mating of 2 distinct ancestral species 17 to 18 million years ago [61]. Approximately 56% of genes in the present-day *X*. *laevis* genome are duplicated between the L and S genomes because of the allotetraploidy event, and the genes encoding the cell-cycle proteins are randomly distributed on either the L or the L and S genomes [61]. However, even if we assumed that this duplication in the genome necessarily leads to a duplication in protein abundance, the enrichment in cell cycle was still observed. Therefore, it would appear that allotetraploidy alone cannot account for these alterations in protein abundance in *X*. *laevis*.

Recently, it has been reported that adult *X*. *tropicalis* but not *X*. *laevis* undergo cardiac regeneration upon injury. One possibility may be the difference in age of the animals used in the 3 different studies [62–66]. The tissue used in our study was derived from age-matched female hearts; therefore, the differences in cell-cycle proteins does not appear to relate to aging or sex differences. An alternative hypothesis, in line with the finding here, is animals that can undergo repair induce cardiac cell-cycle genes upon injury. In contrast, animals that cannot undergo repair up-regulate cell-cycle proteins upon terminal differentiation of cardiomyocytes and cannot increase expression of these proteins in response to injury, and therefore, the cardiomcyoytes cannot initiate cell division.

Regeneration not only depends on cell division but also on growth pathways. The target of rapamycin (TOR) pathway is required to control metabolic growth in homeostasis and regeneration [67, 68]. Here, we find *X*. *tropicalis* has an increase in expression in components of the TORC1 arm of the TOR signaling pathway relative to *X*. *laevis* (S8 Table). This raises the possibility that the TORC1 pathway, a pathway involved in nutrient sensing and growth, is at least in part responsible for the injury repair in *X*. *tropicalis*.

Because of its size, anatomy, and physiology, the pig has been a system of choice in industry for modeling numerous cardiac disease states, including atherosclerosis [69], myocardial infarction, and Tetralogy of Fallot (TOF), as well as for testing therapeutic treatments such as collagen injections and catheter procedures [70]. However, the pig appears to be a poor model for ischemia/reperfusion, for example [71], and may not accurately mimic all disease states, as demonstrated by the failure of some pig-proven drugs to have effects on humans [72, 73]. Correspondingly, we found that Kcp was enriched in cardiac abundance in *Xenopus* compared to the mammalian species examined here and that deletion of Kcp in *Xenopus* resulted in severe cardiac defects. Thus, our findings, and the resources we make available for further study, will enable researchers to determine the most appropriate model systems for exploring the molecular mechanisms for specific cellular events of cardiac development and disease.

## Materials and methods

### Ethics statement

All animal procedures were approved by the University of North Carolina Institutional Animal Care and Use Committee (approval numbers 16–273, 18–139, and 18–043), performed in accordance with the National Institute of Health Guide for the Care and Use of Laboratory Animals. The University of North Carolina is accredited by AAALAC and regulated by the United States Department of Agriculture. All efforts were made to minimize animal suffering and the number of animals used. All mice (*M*. *musculus*) were housed in a temperature- and humidity-controlled facility with a 12-hour light/dark cycle. Food and water were available ad libitum. All *Xenopus* animals were housed in Techniplast recirculating systems in a temperature- and humidity-controlled facility with a 12-hour light/dark cycle.

### Reagents used

Unless otherwise noted, all reagents were purchased from Sigma Aldrich (St. Louis, MO).

### Sample preparation for data dependent analysis

Whole heart tissue was removed from age-matched sexually mature female adult animals, flushed with PBS, frozen in liquid nitrogen, and cryogenically homogenized using a mortar and pestle. The *Sus scrofa* female hearts were received (Pel-Freez, Rogers, AR) on wet ice, diced into pieces, snap frozen in liquid nitrogen, and then blended in a stainless-steel Waring blender.

Several fractionation techniques have been shown to be valuable to improve the depth of analysis when studying tissue proteomes. In particular, an elegant study from the Mann group fractionated human heart into 16 different subregions and coupled this with high pH reverse phase fractionation to generate a deep proteome of the human heart [57]. Given our focus on comparing the cardiac proteomes of different species, such a fractionation approach would have been practically challenging given the major differences in the heart sizes and structures between the species studied. Therefore, we opted to homogenize the entire heart for all the species compared and performed a differential extraction and gel fractionation approach to increase access to transcription factors and to deal with the large dynamic range issues inherent to the heart. For each species, frozen powder from the following number of hearts was combined equally for each biological replicate: *M*. *musculus*: 3, *X*. *laevis*: 3, *X*. *tropicalis*: 7, *S*. *scrofa*: 2. The mixed tissue powder was then dissolved in homogenization buffer (10 mM Tris [pH 7.4], 250 mM sucrose, 1 mM EDTA, 0.15% NP-40, 10 mM sodium butyrate, 1 mM PMSF, 1X protease inhibitors, and 1X phosphatase inhibitors). Insoluble material was pelleted out and the remaining supernatant (S1, Extract 1) was put aside for further processing. The pellet (P1) was resuspended in lysis buffer (20 mM K-HEPES [pH 7.4], 110 mM KOAc, 2 mM MgCl2, 0.1% Tween-20, 1 μM ZnCl2, 1 mM CaCl2, 150 mM NaCl, 0.5% Triton X100, 1X protease inhibitors, and 1X phosphatase inhibitors) and then homogenized via Polytron (Kinematica, Bohemia, NY). Remaining insoluble material (P2) was pelleted out, and the supernatant (S2) was subjected to acetone precipitation to concentrate the protein. The resulting acetone pellet was resuspended in lysis buffer (as above, Extract 2). The pellet P2 was resuspended in lysis buffer (as above, Extract 3). Protein yields of all 3 subcellular fractions were assessed by Bradford assay and 25 μg of protein from each fraction was reduced with 25 mM iodoacetamide at room temperature in the dark for 1 h. LDS buffer (Novex, Waltham, MA) and 3 μL of reducing agent (Novex, Waltham, MA) was added to each sample and boiled for 10 min at 70°C. Each sample was then resolved by 4% to 12% Bis-Tris SDS-PAGE (Novex, Waltham, MA) in MOPS buffer (Novex, Waltham, MA) for a length of 1.5 cm into the gel. The gels were then Coomassie stained, cut into 5 fractions, and digested overnight with trypsin (125 ng/gel slice, Pierce, Waltham, MA) in 25 mM ammonium bicarbonate (pH 8.0). Peptides were extracted from the gel using 3 consecutive washes of 60% acetonitrile (ACN, ThermoFisher Scientific, Waltham, MA), 1% formic acid (FA, ThermoFisher Scientific, Waltham, MA), 39% H_2_O with 20 min incubations. The extracted peptides were dried in vacuo and resuspended in 10 μL of 1% FA, 1% ACN, 98% H_2_O for LC-MS/MS analysis.

#### Sample preparation for PRM targeted analysis

Independent heart tissues from sexually mature adults were thawed on ice, minced into small pieces, and homogenized in 50 mM Tris-HCl (pH 8.0), 100 mM NaCl, and 0.5 mM EDTA by douncing in a Tenbroeck homogenizer (ThermoFisher Scientific, Waltham, MA). The homogenate was then mixed 1:1 with the same buffer containing 4% SDS for a final SDS concentration of 2%. One whole heart per replicate was used except for *S*. *scrofa* hearts, in which 300 mg of ground powder (as above for the data dependent samples) from a single heart was used instead of intact heart tissue. The homogenized tissues in the SDS buffer were then heated at 95°C for 5 min and then sonicated in a cup horn sonicator (1 s pulses, medium power) for 20 s. The heating and sonication process was repeated 3 times until little or no insoluble material was visible. Samples were centrifuged to pellet any remaining insoluble material and a BCA assay (Pierce) was performed to assess protein concentration. A total of 50 μg of protein from each sample was simultaneously reduced and alkylated with 20 mM tris(2-carboxyethyl)phosphine (Pierce, Waltham, MA) and chloroacetamide (ThermoFisher Scientific, Waltham, MA), respectively, for 20 min at 70°C. Protein samples were then cleaned up by methanol-chloroform precipitation [74]. Briefly, LC-MS grade methanol, chloroform, and water (at a 4:1:3 ratio, ThermoFisher Scientific, Waltham, MA) were added to the sample with vortexing following each addition. The samples were spun at 2,000*g* for 5 min at room temperature and the top phase was removed. Three volumes of cold methanol were then added, and the samples were spun at 9,000*g* for 2 min at 4°C. All liquid was removed, and the protein pellets were washed with 5 volumes of cold methanol and then spun at 9,000*g* for 2 min at 4°C. All liquid was removed again, and the dried protein pellets were resuspended in 50 mM HEPES (pH 8.5) at a 0.5 μg/μL concentration. Trypsin (Pierce, Waltham, MA) was added at a 1:50 protease:protein ratio, and the samples were incubated at 37°C overnight. Peptides were cleaned up via SDB-RPS StageTip as described by Sauls and colleagues [75], briefly, peptides were acidified, bound to StageTips, washed with 0.5% trifluoroacetic acid (ThermoFisher Scientific, Waltham, MA) in H_2_O, and eluted directly into autosampler vials (ThermoFisher Scientific, Waltham, MA) with 5% ammonium hydroxide, 80% ACN, 15% H_2_O. Peptides were dried in vacuo and resuspended in 1% ACN, 1% FA, 98% H_2_O, and peptide concentrations were assessed by A205 assay on a Nanodrop One (ThermoFisher Scientific, Waltham, MA). Samples were adjusted to equal concentrations and analyzed by LC-MS/MS.

### MS analyses for DDA

Peptides (5 μL) were analyzed by LC-MS/MS using an EasyNano nLC1000 UHPLC (Thermo Scientific, Waltham, MA) coupled online to an EASYSpray ion source and Q Exactive HF (Thermo Scientific, Waltham, MA). Peptides were separated on an EASYSpray C18 column (75 μm ×50 cm, ThermoFisher Scientific, Waltham, MA) heated to 50°C using a gradient of solvents A (0.1% FA in H_2_O) and B (0.1% FA, 2.9% H_2_O in ACN) at a flow rate of 250 nL/min as follows: 5% B to 20% B over 40 min, then to 32% B in 11 min, then to 90% B in 2 min, and hold at 90% B for 12 min. Peptides were ionized at 2.5 kV and an MS1 survey scan was performed from 400 to 1,600 m/z at 120,000 resolution with an automatic gain control (AGC) setting of 3 × 10^6^ and a maximum injection time (MIT) of 100 ms recorded in profile. The top 15 precursors were then selected for fragmentation and MS2 scans were acquired at a resolution of 15,000 with an AGC setting of 2 × 10^4^, a MIT of 50 ms, an isolation window of 1.6 m/z, normalized collision energy of 27, intensity threshold of 2 × 10^4^, peptide match set to preferred, and a dynamic exclusion of 30 s recorded in profile. MS/MS data were analyzed by Proteome Discoverer (Thermo Fisher Scientific, Waltham, MA, version 2.1.1.21, https://www.thermofisher.com/order/catalog/product/OPTON-30795). A fully tryptic Sequest HT search against a combined Uniprot database containing human, *M*. *musculus*, *X*. *laevis*, *X*. *tropicalis*, and *S*. *scrofa* canonical protein sequences appended with common contaminants (141,645 entries, downloaded 2016_12) was conducted. Sequest settings required peptides to have 5 ppm accuracy on the precursor and 0.02 Da accuracy on the fragments. Allowed peptide modifications included static carbamidomethyl modifications to cysteine, dynamic oxidation of methionine, dynamic deamidation of asparagine, and dynamic methionine loss, and acetylation of protein n-termini. The precursor ions area detector node in Proteome Discoverer was used to allow MS1 quantitation. The resulting peptide spectrum matches were imported into Scaffold (Proteome Software, Portland, Oregon, version 4.7.3, http://www.proteomesoftware.com/products/scaffold/) with an X!Tandem rescore with phosphorylations on STY included as additional modifications. The protein clustering algorithm in Scaffold was used, and the data were assembled at a 2 peptide/protein level with a 99.9% protein probability and 96% peptide probability. This resulted in a final protein and peptide FDR of 0.9% and 0.11%, respectively. The total (summed abundance) precursor intensity quantitation in Scaffold was utilized for all subsequent analysis. The proteins included in quantitation were those observed either with a minimum of 2 unique peptides in both replicates or with 4 or more unique peptides in one replicate. All detected peptides for a protein meeting the above conditions were used for quantitation, including peptides common among all species and peptides unique for one or more species.

### Targeted quantification by PRM

Peptides (2 μL) were analyzed by LC-MS/MS using a Dionex Ultimate 3000 UPLC (Thermo Scientific) coupled online to an EASYSpray ion source and Q Exactive HF. Peptides were separated on an EASYSpray C18 column (75 μm × 25 cm or 75 μm × 50 cm) heated to 50°C at a flow rate of 250 nL/min as follows: 1% B to 4% B over 9 min, then to 14% B over 40 min, then to 25% B over 20 min, then to 50% B over 4 min, then to 97% B over 0.5 min, then hold at 97% B for 6 min, then to 70% B over 0.5 min. A peptide inclusion list was generated with retention time windows of 5 min used to isolate precursors for fragmentation. Peptides were ionized at 1.7 kV, and a scheduled PRM method was run in which MS2 scans were acquired at a resolution of 30,000 with an AGC setting of 2 × 10^5^, a MIT of 150 ms, an isolation window of 0.8 m/z, fixed first mass of 125.0 m/z, and normalized collision energy of 27 recorded in profile. After every 15 MS2 scans, a single MS1 scan was performed from 380 to 1,800 Da at a resolution of 15,000 and an AGC setting of 3 × 10^6^. Label-free targeted quantitation of peptides specific for a protein of interest and with identical sequences between all 4 species was designed and analyzed using the Skyline software. Summed area under the curve of 3 to 4 transitions per peptide was used for quantitation. The peptide peak areas were normalized by the average MS1 intensity of that sample as reported by RawMeat (Vast Scientific). Peptide values for each sample were scaled to the average of each peptide across all runs. The average of multiple peptides was used as the inferred value for the protein measurement when more than one peptide was quantified. Differences across species were assessed using an ANOVA analysis with a pairwise Bonferroni’s post-test between *X*. *laevis* and each other species in Prism version 5.04 (GraphPad Software, San Diego, CA, https://www.graphpad.com/scientific-software/prism/).

### Computational prediction of protein complex abundance and conservation among species

Protein complex clustering was performed by mapping the identified proteins to human protein complexes in CORUM. To focus on major macromolecular assemblies, protein complexes had to contain a minimum of 3 protein members to pass our criteria for further investigation. In order to ensure accurate assessment of the presence and abundance of complexes, each complex analyzed with 5 member proteins or less had to be observed with at least 40% coverage within at least 1 species. For those complexes with 6 or more member proteins, only 20% identification of the complex components in at least 1 species was required. A weighted mean complex score, represented by the mean value of the precursor area in all quantified components multiplied by the percent of complex coverage in that species, was then calculated. The scored complexes were clustered in R using the hierarchical clustering algorithm via the default ward method. The resulting dendrogram was cut at a level that generated 6 clusters based on evaluation of the clustering via PCA. The clustered complexes were then assessed for functional annotation. For the analysis of cardiac-specific protein complexes using the BioSNAP database (https://snap.stanford.edu/biodata/datasets/10013/10013-PPT-Ohmnet.html), we utilized the reported binary interactions to generate the maximum possible set of protein complexes with at least 3 protein members. All scoring and clustering were performed as above for the CORUM analysis. The resulting complex data was grouped into 8 clusters based on the hierarchical clustering.

### Statistical analysis and data visualization

Biological duplicates of each species were analyzed by LC-MS/MS. Identified proteins were exported from Scaffold, and median normalization of the MS1 abundance values was performed in Excel (Microsoft, https://products.office.com/en-us/excel). The identified proteins were mapped to human accession numbers using Blast2GO (BioBam, version 5.0.13, https://www.blast2go.com/) [76] by performing a local blastp search against a human proteome file containing 20,205 canonical protein sequence entries using default settings to return the top blast hit for each protein. By mapping from less complete FASTA files (the 4 species analyzed) to a more complete one (human), we avoided many issues in mapping against partial or missing sequence information. The resulting mapped proteins contained multiple entries that mapped to the same human accession number, and these entries were averaged together to attain a single set of MS1 abundance values for each mapped human protein. Differential proteins were analyzed via overrepresentation analysis and gene set enrichment analysis using www.pantherdb.org [77] (version 13.1) for associated gene ontology enrichments with multiple comparison *p*-value adjustments. Tree maps were made using REVIGO [78]. Example proteins of different classes were graphed in GraphPad Prism version 5.04, and heat maps of protein classes were generated using Morpheus (Broad Institute, https://software.broadinstitute.org/morpheus/). Principal component analysis was performed in Perseus version 1.6.0.2 [79]. Numbers of theoretical tryptic peptides for each protein across species were generated using the Proteome Ruler plugin of Perseus [80]. Network diagrams were created in Cytoscape (version 3.6.1) [81] using the Reactome (version 7.0.2) [82] and ClueGO (version 2.5.1) [83] plugins. The mass spectrometry data was deposited to the PRIDE [84] and Panorama [85] databases.

### Code availability

The R script used in the computational prediction of protein complex abundance and conservation among species analysis is provided as a supplemental download with this manuscript.

### *Xenopus* manipulations

*Xenopus* embryos were cultured, microinjected, and staged according to Niewkoop and Faber [86, 87]. *Xla*.*Tg(Cardiac-actin*:*GFP)*^*Mohun*^ transgenic frogs were used as previously reported by Tandon and colleagues and Latinkic and colleagues [87, 88]. Animals were anaesthetized in tricaine according to IACUC procedures.

### TALEN design and *Xenopus* mutation analysis

TALENs targeting the second coding exon of *kcp* were designed using the online software TAL Effector Nucleotide Targeter 2.0 (Cornell University) and constructed with the Golden Gate TALEN and TAL Effector Kit (Addgene) [89] into the pCS2 vector for capped mRNA transcription using mMESSAGE mMachine SP6 Kit (Ambion, Waltham, MA). One-cell stage *Xenopus* wild-type or *Xla*.*Tg(Cardiac-actin*:*GFP)*^*Mohun*^ embryos were microinjected with 1 ng equal mixture of right-arm and left-arm TALEN mRNA to induce mutations. Mutations were confirmed by HOTSHOT genomic DNA preparation followed by PCR. Mutated alleles were identified using the T7 Endonuclease I assay (adapted from [90]), and subsequently cloned and sequenced. Mutant tadpoles were grown to adulthood and crossed with wild-type or *Xla*.*Tg(Cardiac-actin*:*GFP)*^*Mohun*^ frogs to generate F1 embryos and assess germline transmission of *kcp* locus mutations. Kcp mutant founders were subsequently confirmed and mated to generate F1 compound heterozygous Kcp mutant embryos, termed mutant for the duration of the article. Sibling heterozygous Kcp mutants were used as controls in all experiments and termed control. All mutant animals used in experimental analysis were PCR-genotyped and subsequently sequenced to verify the incorporation of mutations into both *kcp* alleles resulting in a frame-shift and premature stop codon in the LaminG protein domain, prior to the essential cysteine-rich elements.

### Histological sectioning, immunohistochemistry, and BMP/KCP assays

*Xenopus* tadpoles and froglets were fixed in 4% PFA or Bouin’s solution and processed for either paraffin sectioning, cryosectioning, and gelatin/agarose vibratome sectioning (10, 25, and 100 μm, respectively) as reported by Tandon and colleagues, Wallingford, Charpentier and colleagues, Gessert and Kuhl, and Dorr and colleagues [87, 91–94]. Paraffin sections were dewaxed, rehydrated, and stained with hematoxylin–eosin (HE) or Masson Trichrome stains using standard protocols [94]. For BMP/KCP assays, *Xenopus* embryos were obtained and injected with RNA according to Montagner and colleagues and Conlon and Smith [55, 95] and scored according to Montagner and colleagues and Moser and colleagues [55, 96].

#### Ultrasound imaging of kcp mutant cardiac function

Cardiac function was assessed in terminally sedated mutant and control animals by thoracic ultrasound using an Ultrasound Vevo2100 System (Small animal imaging facility, BRIC, UNC). Briefly, froglets were placed dorsal side down onto the stage immersed in a sealed bag containing 0.2% tricaine in frog system water. Ultrasound X imaging gel was then placed onto the surface of the bag. A 30 MHz pediatric probe was used to image cardiac contraction and blood flow (Doppler imaging) in the ventricle and was performed for the duration of 60 s at the stated orientations, after which the animal was humanely euthanized. Studies were conducted blind, and genotype was assessed postimaging analysis.

#### CT imaging of *kcp* mutant hearts

Analysis of cardiac valve morphology was performed using contrast-enhanced CT imaging using a SCANCO microCT 40 scanner (SAIF-BRIC, UNC). KCP mutants (F2, 29 bp deletion) and wild-type sibling froglets (Stage 66, NF) were terminally sedated and fixed in Bouin’s fixative for 48 h at 4°C, then immersed in 100% Ethanol for 48 h. Froglets were then placed in 0.03% PTA contrast reagent in 70% Ethanol for 1 h prior to imaging [97]. Images were analyzed using VivoQuant 2.50 software (inviCRO, LLC, Boston, MA) to provide 3D rendering images of valve tissue.

## Supporting information

S1 Fig Impact of combined search on identifications. (A) The number of sequences present in the FASTA databases for each species varied, with mouse having the most depth. (B) Percent of proteins identified from the 5 searched species in the 4 species analyzed by MS. See S1 Table for numerical data underlying figure. MS, mass spectrometry.
(TIF)

S2 Fig The combined database search approach yielded an increase in IDs. Percent of (A) proteins and (B) peptides identified uniquely in either a species-specific (blue bars) or combined species (gray bars) search. Orange bars represent proteins and peptides found in both search modes. (C) Table of values from (B). See S2 Table for numerical data underlying figure. ID, Identification.
(TIF)

S3 Fig Total proteins identified. (A) *M*. *musculus*, (B) *S*. *scrofa*, (C) *X*. *laevis*, and (D) *X*. *tropicalis*.
(TIF)

S4 Fig Examination of extract subproteomes. GO cellular component terms and *p*-value enrichments are shown for terms enriched in each extract. GO, Gene Ontology.
(TIF)

S5 Fig Number of proteins identified in each replicate by species. See S5 Table for numerical data underlying figure.
(TIF)

S6 Fig Assessment of tryptic peptide frequency across species. (A) The distribution of theoretical tryptic peptides present in the FASTA databases used in this study was significantly different between all species (*p* < 0.0001). (B) However, no significant difference in the distribution of theoretical tryptic peptides was observed across the 4 species for the proteins analyzed in this data set. Whiskers show 1 to 99 percentile. See S7 Table for numerical data underlying figure.
(TIF)

S7 Fig Multiscatter plot of median-normalized precursor values across the 4 species. The 2 *Xenopus* species exhibit the greatest degree of similarity.
(TIF)

S8 Fig PCA analysis of proteins identified in each extract from each species. See S3 Table for numerical data underlying figure. PCA, principal component analysis.
(TIF)

S9 Fig All identified proteins that could be mapped to human entries. The data in Fig 1E includes quantified proteins only. This schematic also includes any identified but not quantified proteins.
(TIF)

S10 Fig Tree map of significantly enriched GO biological process terms in the core cardiac proteome. This is an extended version of Fig 1F that includes all enriched GO terms. The size of the box correlates to the significance of enrichment for that term with larger box sizes being more significant. GO, Gene Ontology.
(TIF)

S11 Fig Workflow for protein complex abundance analysis related to Fig 2. (TIF)

S12 Fig Individual clusters 3 (A) and 5 (B) from the protein complex clustering in Fig 2 are shown and the top 3 complexes from each cluster are listed (C). See S9 Table for numerical data underlying figure.
(TIF)

S13 Fig Treemap of GO terms associated with protein complexes that have minimal variation across species. GO, Gene Ontology.
(TIF)

S14 Fig BioSNAP complex clustering. (A) The 8 clusters resulting from mapping the 4 species data set to cardiac complex information in BioSNAP are shown. One of the top GO terms for each cluster is show in blue. (B) Comparison of BioSNAP and CORUM complex analysis. Top enriched GO terms for each unique set of proteins are listed. See S10 Table for numerical data underlying figure. CORUM, Comprehensive Resource of Mammalian Protein Complexes; GO, Gene Ontology.
(TIF)

S15 Fig Analysis of differential abundance of shared cardiac proteins. (A) The 1,770 proteins that are shared across all 4 species were clustered using k means with k = 6 revealing clusters driven by higher protein expression in one or more species. (B) Each of the clusters from the heat map were analyzed for overrepresentation of GO biological process terms and the *p*-values of the significantly enriched terms were shown in a heat map. See S8 Table and S11 Table for numerical data underlying figure. GO, Gene Ontology.
(TIF)

S16 Fig Analysis of species unique proteins. (A) The proteins uniquely identified in the mammalian and *Xenopus* species were used to examine evolutionary driven protein expression differences in these organisms using ClueGO (B) Proteins found uniquely in one species only were combined with the cluster of proteins that were more highly expressed in that species and analyzed for overrepresentation of GO biological process terms. The *p*-values of the significantly enriched terms are shown in a heat map. See S12 Table for numerical data underlying figure. GO, Gene Ontology.
(TIF)

S17 Fig Cell cycle GO enrichments in every pairwise comparison with X. laevis. (A) *M*. *musculus*/*X*. *laevis*; (B) *S*. *scrofa*/*X*. *laevis*; (C) *X*. *tropicalis*/*X*. *laevis*. See S13 Table and S14 Table for numerical data underlying figure. GO, Gene Ontology.
(TIF)

S18 Fig Gene duplication corrected cell-cycle analysis. Data from Fig 3 were reproduced here with correction for gene duplication in *X*. *laevis*. Similar trends were observed as before. See S15 Table for numerical data underlying figure.
(TIF)

S19 Fig Pairwise GSEA analysis of heart proteomes. (A) *M*. *musculus*/*S*. *scrofa*; (B) *M*. *musculus/X*. *laevis*; (C) *M*. *musculus/X*. *tropicalis*; (D) *S*. *scrofa/X*. *laevis*; (E) *S*. *scrofa/X*. *tropicalis*; (F) *X*. *laevis/X*. *tropicalis*. See S13 Table and S14 Table for numerical data underlying figure. GSEA, Gene Set Enrichment Analyses.
(PDF)

S20 Fig Normalized peptide peak areas from PRM assays targeting cell-cycle proteins enriched in X. laevis. Gene names are listed above the graphs. Proteins for which *X*. *laevis* was significantly higher than the other species are noted with an asterisk. (*p ≤ 0.05, **p ≤ 0.01, ***p ≤ 0.001, ****p ≤ 0.0001). See S16 Table for numerical data underlying figure. PRM, parallel reaction monitoring.
(PDF)

S21 Fig Sequence alignment of Kcp across species. Kcp protein sequences from mouse, human, *X*. *laevis*, *X*. *tropicalis*, and pig were aligned to examine sequence conservation. Highlighted in dark blue are residues conserved among all 5 species, medium blue shows conservation in 4 species, and light blue depicts sequence conservation in 3 species. Kcp, Kielin/chordin-like protein.
(TIF)

S22 Fig Kcp augments BMP signaling. (A) Control embryos; mock injected. (B) Embryos injected with 500 pg Kcp mRNA. Embryos display protrusions (red arrows). (C) Embryos injected with 500 pg Bmp2 mRNA. Embryos displaying partial double axis (red arrows). (D) Embryos injected with 500 pg Kcp mRNA + 500 pg Bmp2 mRNA. Embryos displaying partial double axis, posterior truncations, edema. (E) Graph of the classes of phenotypic abnormalities in wild-type embryos verses those injected with Kcp alone, BMP2 alone, or BMP2 + Kcp. Results from 2 independent biological replicates with total number of embryos scored under each heading. See S17 Table for numerical data underlying figure. BMP, bone morphogenetic protein; Kcp, Kielin/chordin-like protein.
(TIF)

S1 Code R script used to perform CORUM analysis. CORUM, Comprehensive Resource of Mammalian Protein Complexes.
(R) Click here for additional data file.

S2 Code R script used to perform BioSNAP analysis. (R)

S1 Table Numbers of proteins identified in each organism from each species FASTA file. (XLSX)

S2 Table Number of proteins and peptides identified in species-specific FASTA searches, species-combined FASTA searches, or both. (XLSX)

S3 Table Exported fractionated data from Scaffold with MS1 quant values. (XLSX)

S4 Table Exported fractionated data mapped to human accessions. If the protein was present in either of the replicates for the extract it is marked as “Detected”.
(XLSX)

S5 Table Exported un-normalized data from Scaffold with mapped human accession numbers indicated. (XLSX)

S6 Table Normalized and replicate averaged MS1 values for heart proteomes. (XLSX)

S7 Table Number of theoretical tryptic peptides present in each protein across species. (XLSX)

S8 Table MS1 values of mapped human proteins with duplicates averaged. (XLSX)

S9 Table Results of CORUM protein complex analysis. The table below lists the complex components, scores of each complex in each species, and the cluster to which each complex belongs. CORUM, Comprehensive Resource of Mammalian Protein Complexes.
(XLSX)

S10 Table Results of protein complex analysis from binary BioSNAP data. The table below lists the complex components, scores of each complex in each species, and the cluster to which each complex belongs.
(XLSX)

S11 Table Enrichment of GO terms in species-enriched shared proteome. GO, Gene Ontology.
(XLSX)

S12 Table GO enrichments for species-enriched proteins and GO enrichment from ClueGO. GO, Gene Ontology.
(XLSX)

S13 Table Fold change values used for GSEA. GSEA, Gene Set Enrichment Analyses.
(XLSX)

S14 Table Enrichment results from pairwise GSEA. GSEA, Gene Set Enrichment Analyses.
(XLSX)

S15 Table *X. laevis* gene duplication corrected values for cell-cycle enrichment. (XLSX)

S16 Table Peptides targeted for PRM analysis. PRM, parallel reaction monitoring.
(XLSX)

S17 Table Quantitation of phenotypic outcomes of WT, Kcp, Bmp2, and Kcp + Bmp injected embryos. Kcp, Kielin/chordin-like protein; WT, wild type.
(XLSX)

S1 Movie Ultrasound Doppler of living Kcp^Δexon2/Δexon2^ froglets at Stage 64 positioned with dorsal top, ventral bottom of image. Blood flow shown in red.
(MP4)

S2 Movie Ultrasound Doppler of living wild-type froglets at Stage 64 positioned with dorsal top, ventral bottom of image. Blood flow shown in red.
(MP4)

S3 Movie Independent bio-replicate of samples in S1 and S4 Movies. **Ultrasound Doppler of living Kcp**^**Δexon2/Δexon2**^ **froglets at Stage 64 positioned with dorsal top, ventral bottom of image.** Blood flow shown in red.
(MP4)

S4 Movie Independent bio-replicate of samples in S1 and S3 Movies. **Ultrasound Doppler of living Kcp**^**Δexon2/Δexon2**^ **froglets at Stage 64 positioned with dorsal top, ventral bottom of image.** Blood flow shown in red.
(MP4)

S5 Movie Sagital Doppler ultrasound through the ventricle of wild-type froglet (Stage 64) positioned at the ventricle. (MP4)

S6 Movie Sagital Doppler ultrasounds through the ventricle of Kcp^Δexon2/Δexon2^ froglet (Stage 64) positioned at the ventricle. Note alteration in blood flow compared to S5 Movie.
(MP4)

## Abbreviations

ACN: acetonitrile
AGC: automatic gain control
AVSD: atrioventricular septal disease
AVV: atrioventricular valve
CORUM: Comprehensive Resource of Mammalian Protein Complexes
FA: formic acid
GO: Gene Ontology
GSEA: Gene Set Enrichment Analyses
HE: hematoxylin–eosin
Kcp: Kielin/chordin-like protein
LC-MS/MS: liquid chromatography coupled with tandem mass spectrometry
MIT: maximum injection time
MS: mass spectrometry
MS1: precursor ion
PCA: principal component analysis
PRM: parallel reaction monitoring
TALEN: transcription activator-like effector nucleases
