# Supplementary figures and images for: Conservation and divergence of protein pathways in the vertebrate heart
Source: PLoS Biol. 2019 Sep 6;17(9):e3000437. doi: 10.1371/journal.pbio.3000437 (PMC6750614; doi:10.1371/journal.pbio.3000437)

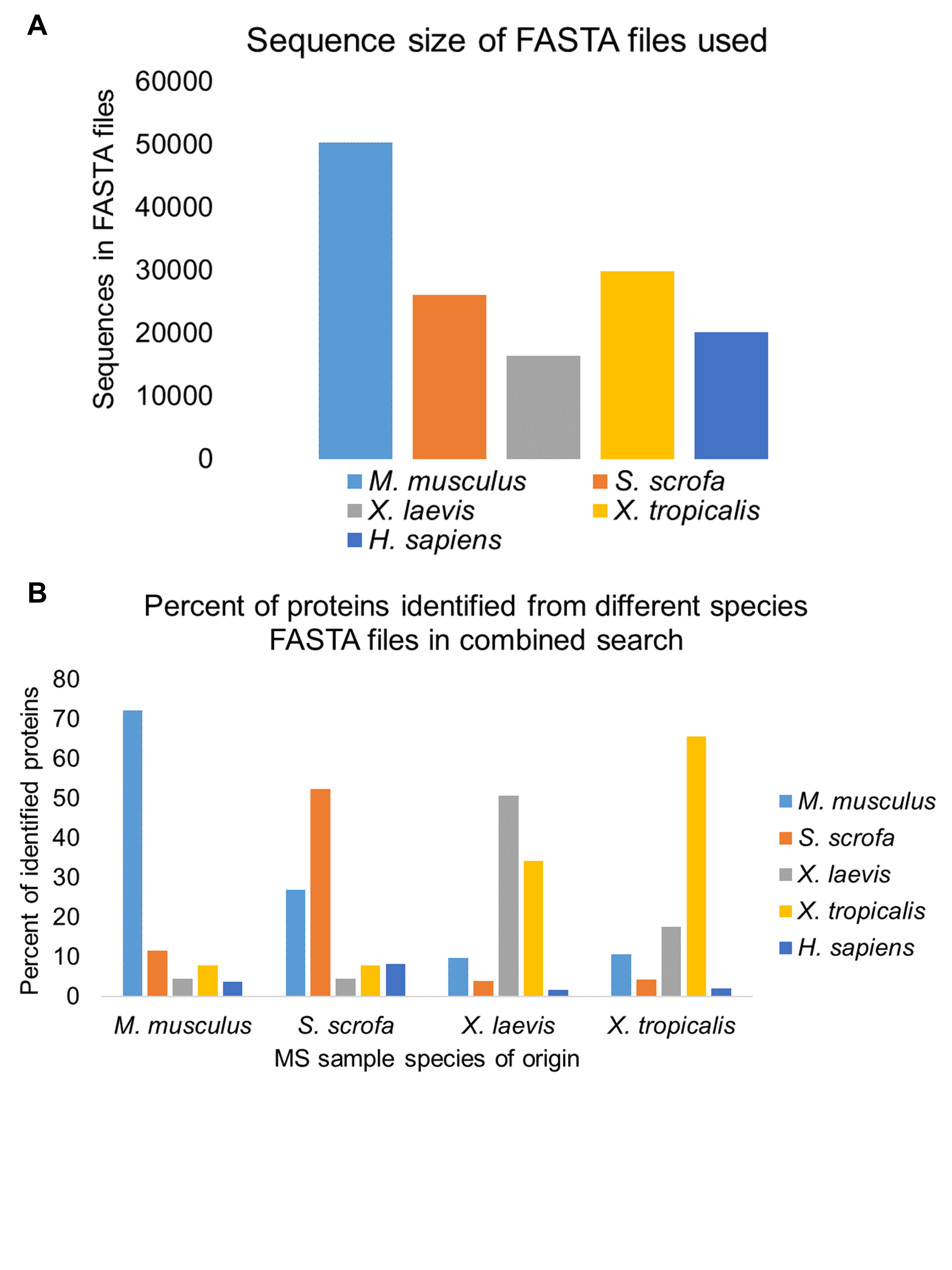

Supplement: S1 Fig — (A) The number of sequences present in the FASTA databases for each species varied, with mouse having the most depth. (B) Percent of proteins identified from the 5 searched species in the 4 species analyzed by MS. See S1 Table for numerical data underlying figure. MS, mass spectrometry. (TIF) [file pbio.3000437.s001.TIF]

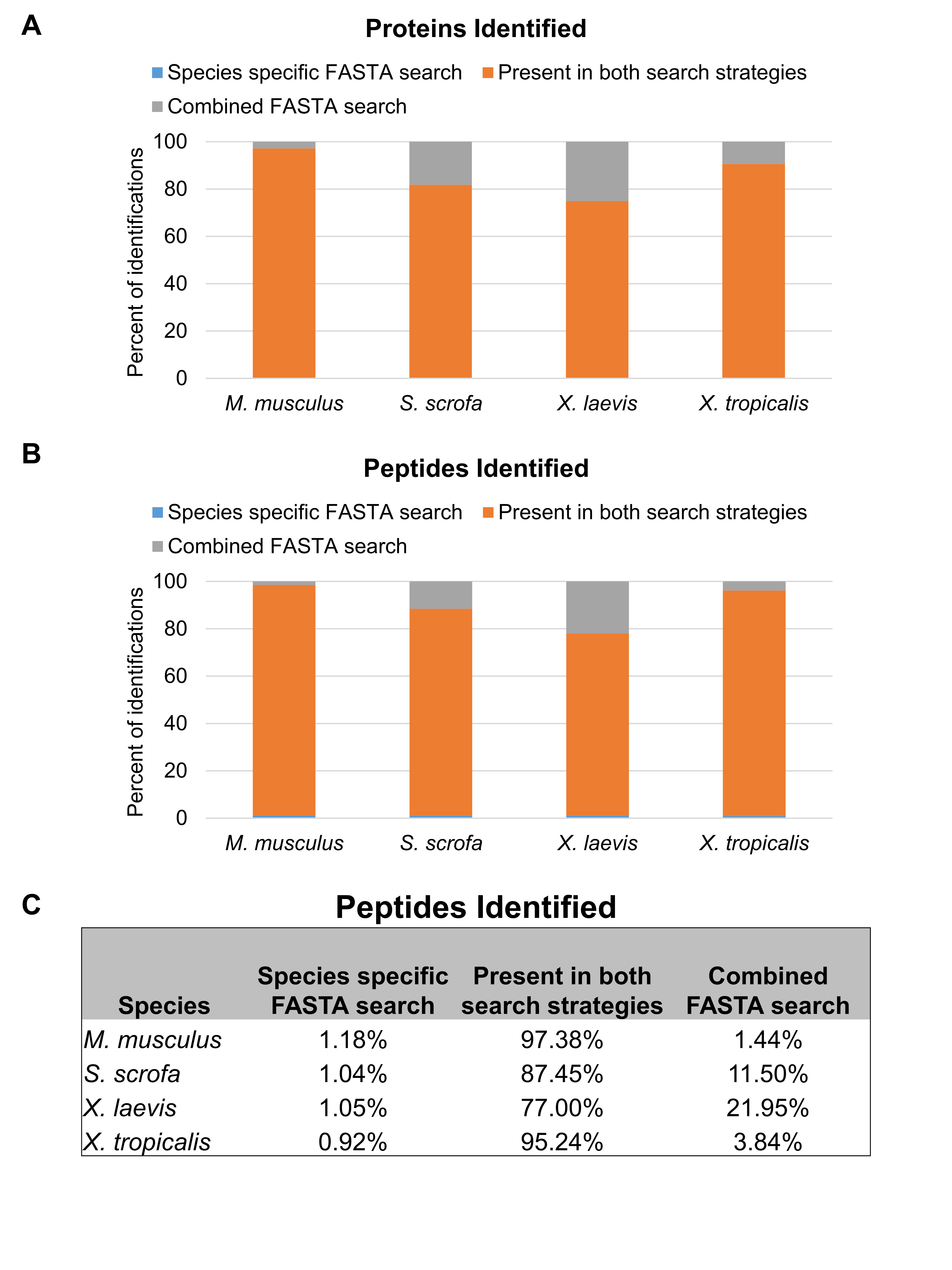

Supplement: S2 Fig — Percent of (A) proteins and (B) peptides identified uniquely in either a species-specific (blue bars) or combined species (gray bars) search. Orange bars represent proteins and peptides found in both search modes. (C) Table of values from (B). See S2 Table for numerical data underlying figure. ID, Identification. (TIF) [file pbio.3000437.s002.TIF]

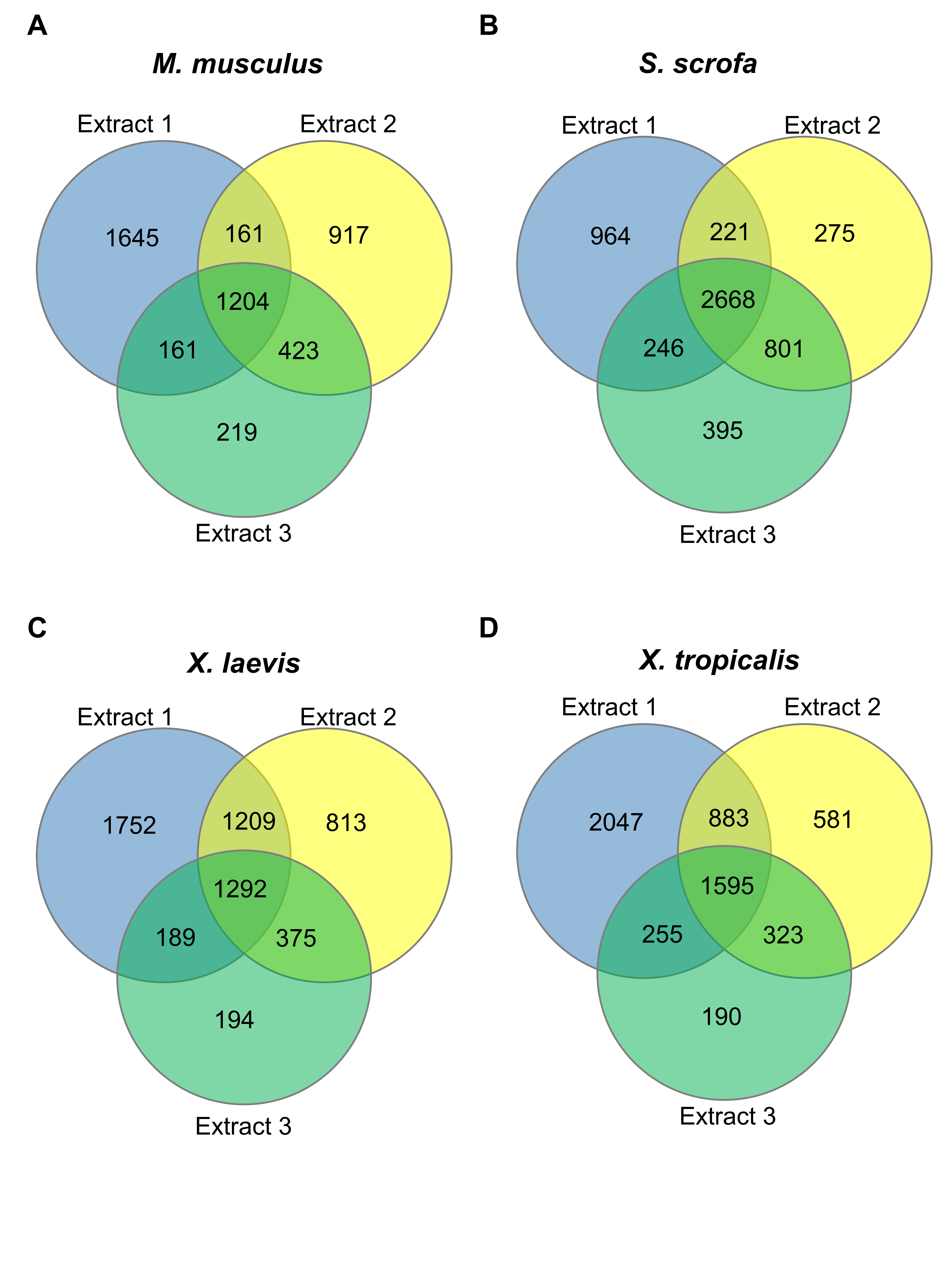

Supplement: S3 Fig — (A) M. musculus, (B) S. scrofa, (C) X. laevis, and (D) X. tropicalis. (TIF) [file pbio.3000437.s003.TIF]

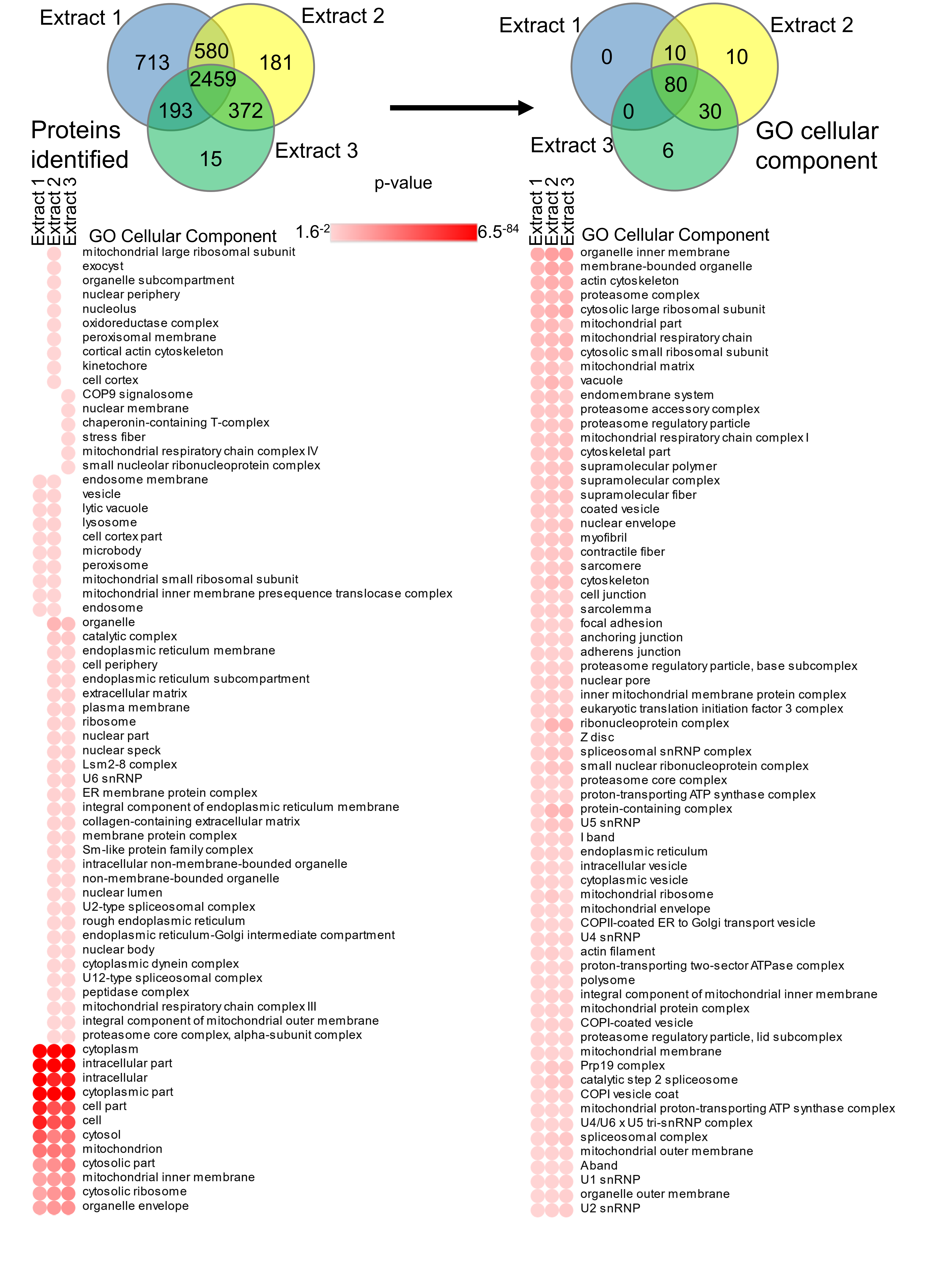

Supplement: S4 Fig — GO cellular component terms and p-value enrichments are shown for terms enriched in each extract. GO, Gene Ontology. (TIF) [file pbio.3000437.s004.TIF]

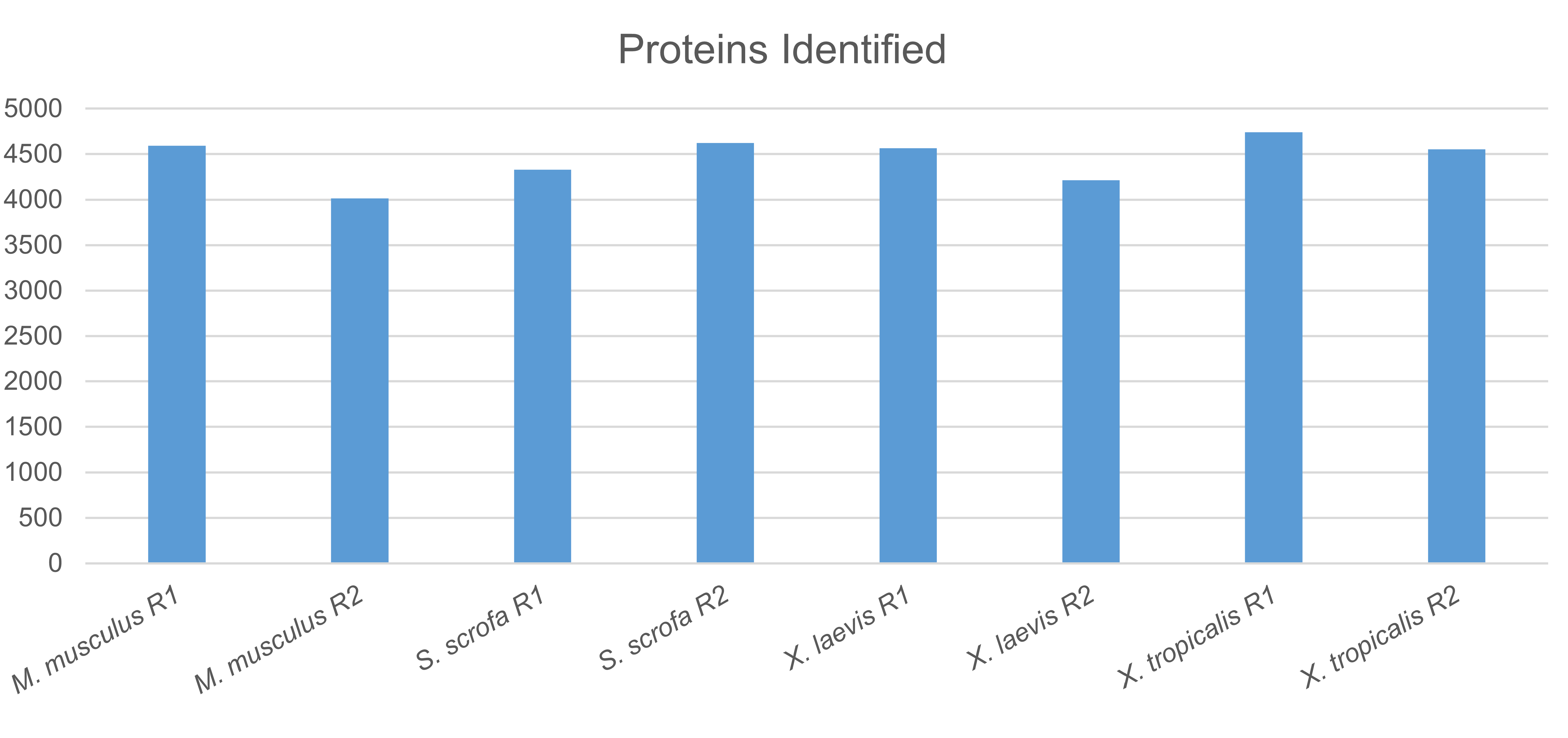

Supplement: S5 Fig — See S5 Table for numerical data underlying figure. (TIF) [file pbio.3000437.s005.TIF]

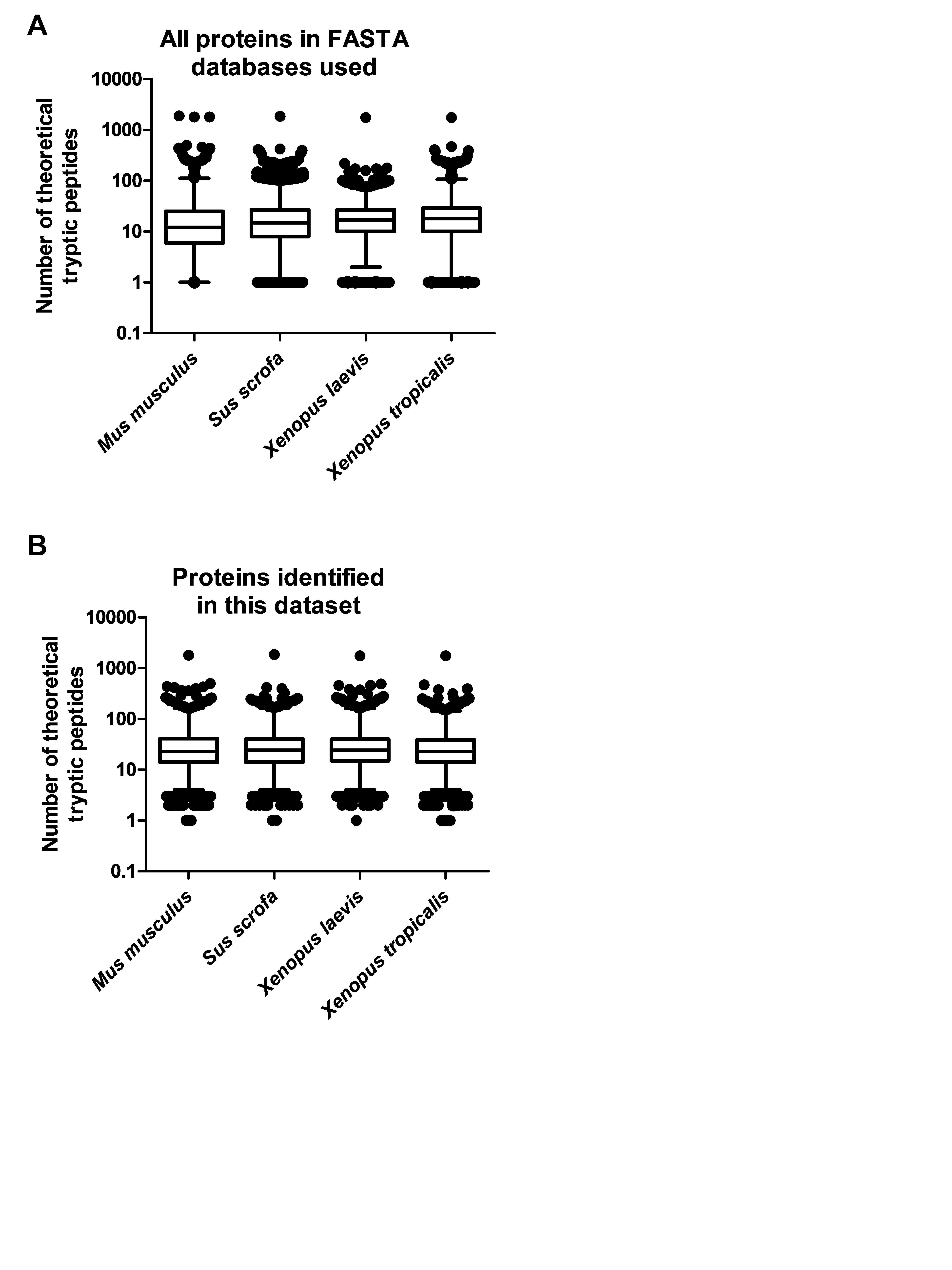

Supplement: S6 Fig — (A) The distribution of theoretical tryptic peptides present in the FASTA databases used in this study was significantly different between all species (p < 0.0001). (B) However, no significant difference in the distribution of theoretical tryptic peptides was observed across the 4 species for the proteins analyzed in this data set. Whiskers show 1 to 99 percentile. See S7 Table for numerical data underlying figure. (TIF) [file pbio.3000437.s006.TIF]

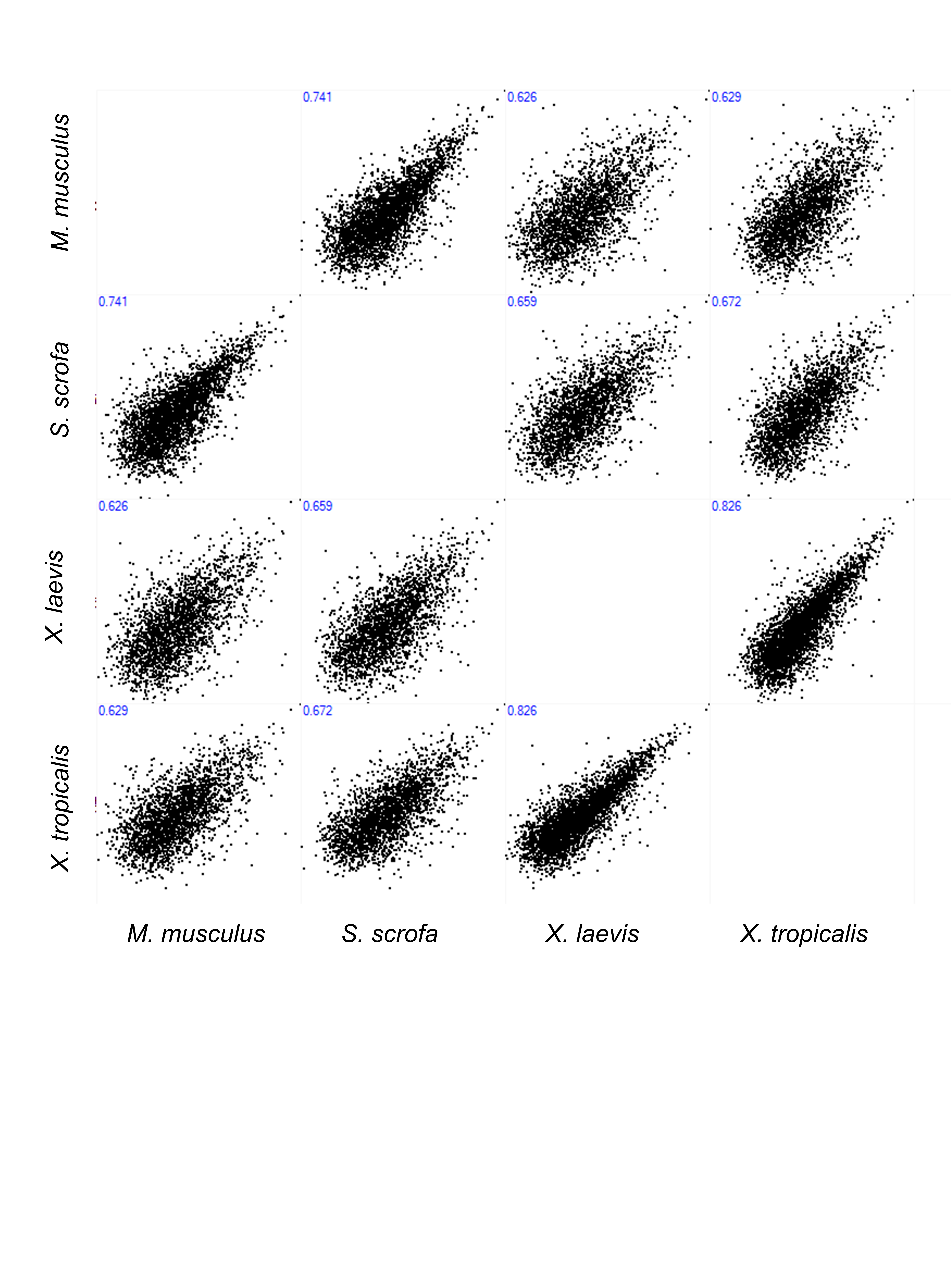

Supplement: S7 Fig — The 2 Xenopus species exhibit the greatest degree of similarity. (TIF) [file pbio.3000437.s007.TIF]

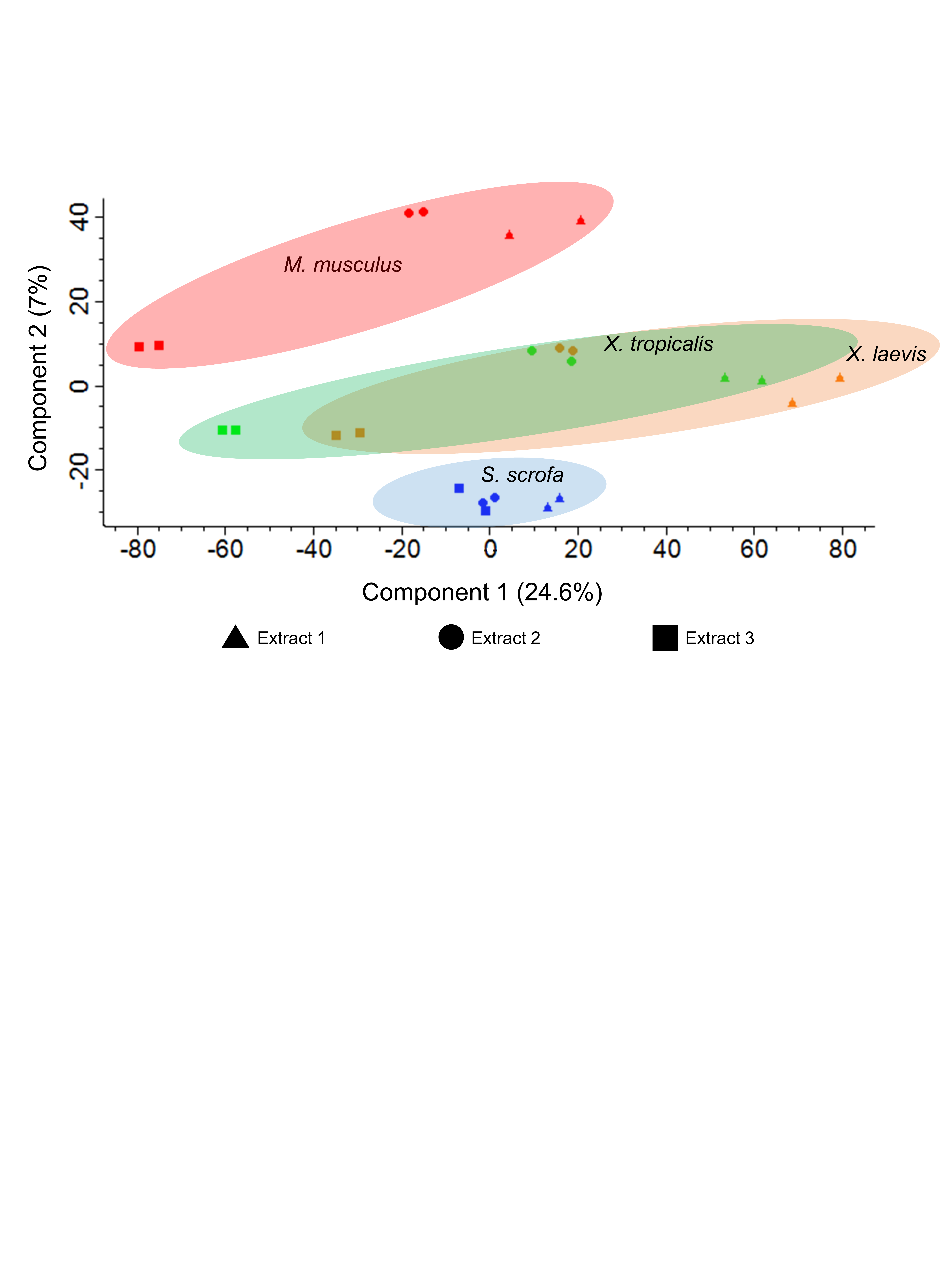

Supplement: S8 Fig — See S3 Table for numerical data underlying figure. PCA, principal component analysis. (TIF) [file pbio.3000437.s008.TIF]

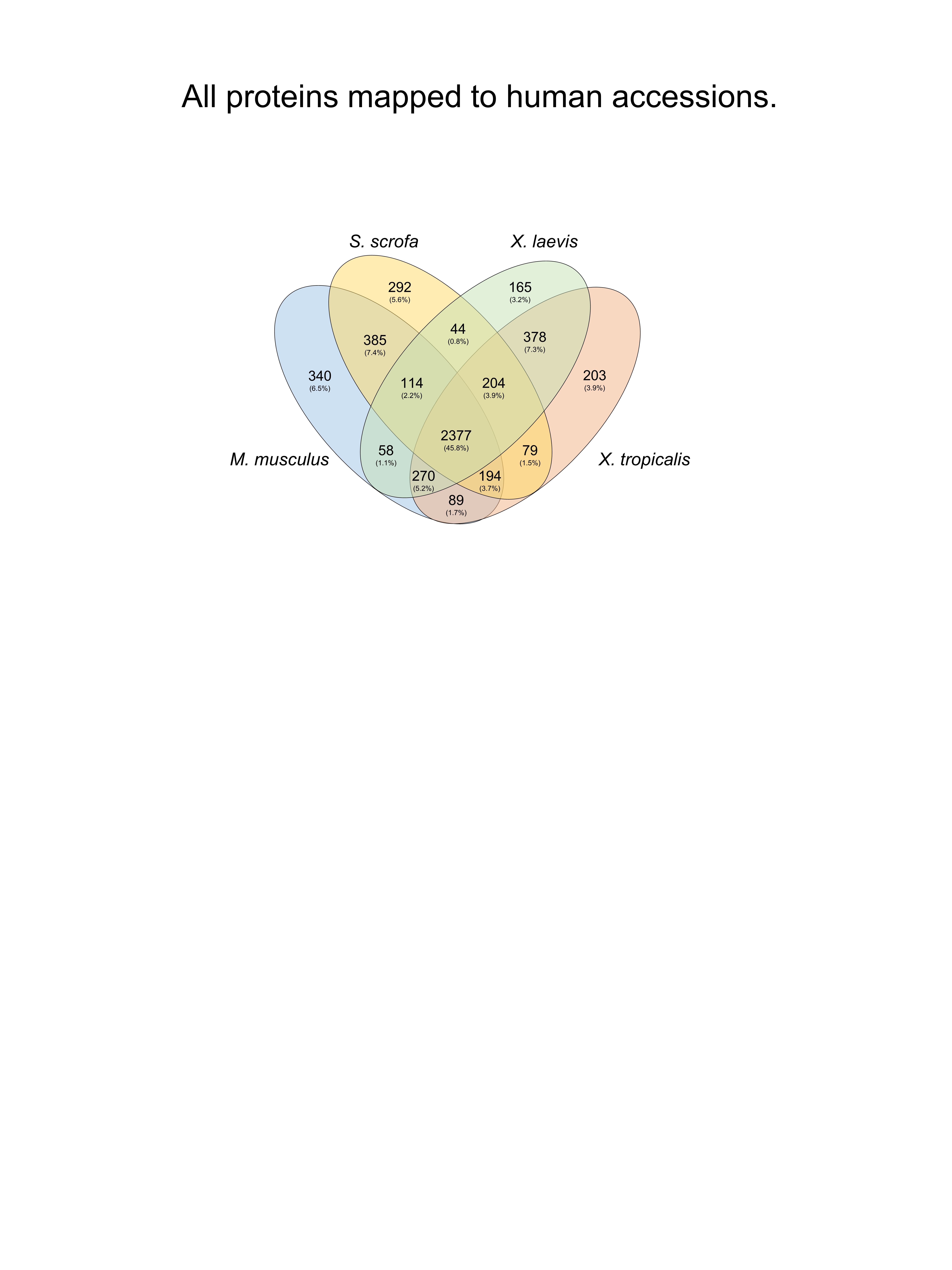

Supplement: S9 Fig — The data in Fig 1E includes quantified proteins only. This schematic also includes any identified but not quantified proteins. (TIF) [file pbio.3000437.s009.TIF]

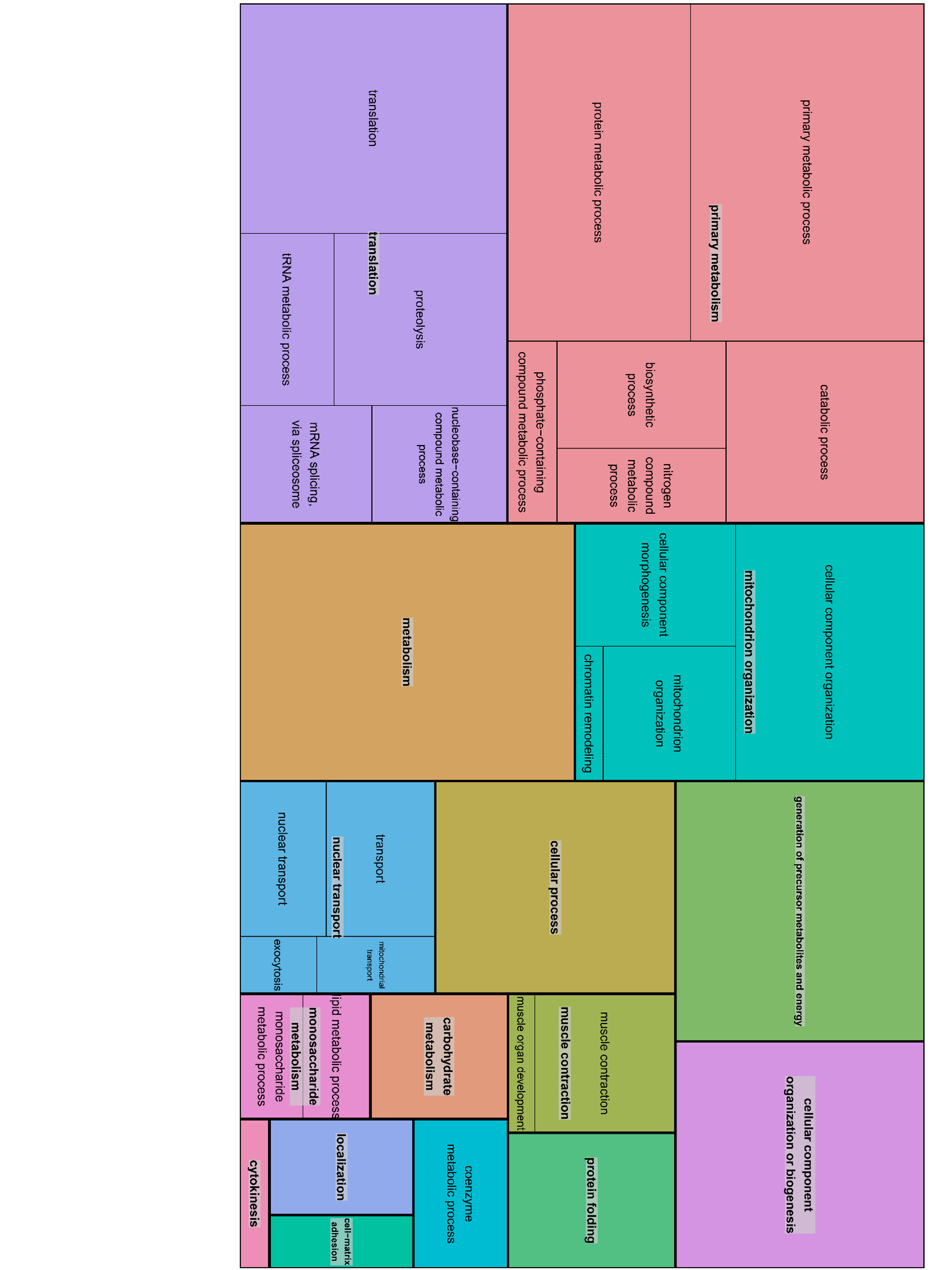

Supplement: S10 Fig — This is an extended version of Fig 1F that includes all enriched GO terms. The size of the box correlates to the significance of enrichment for that term with larger box sizes being more significant. GO, Gene Ontology. (TIF) [file pbio.3000437.s010.TIF]

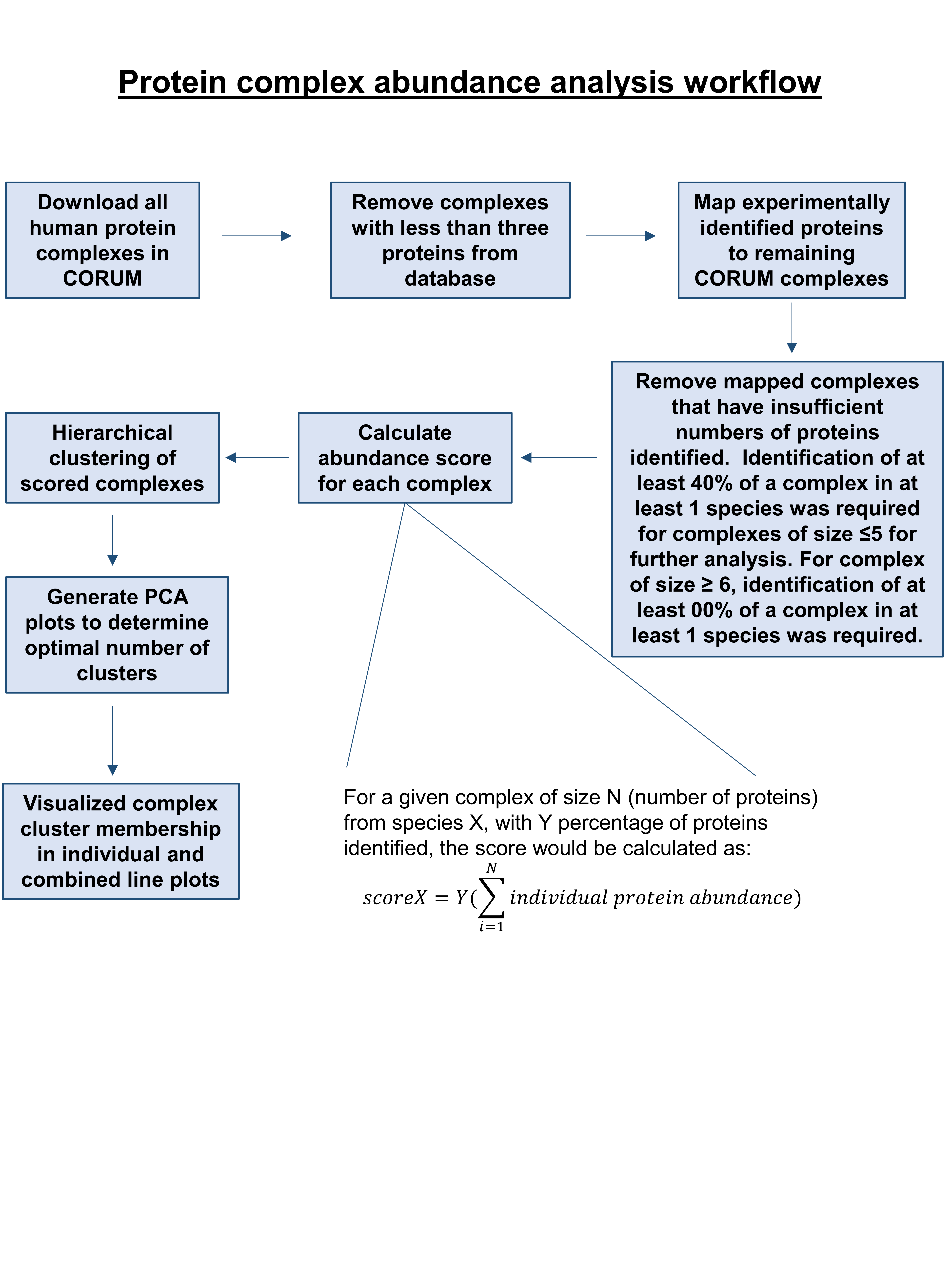

Supplement: S11 Fig — (TIF) [file pbio.3000437.s011.TIF]

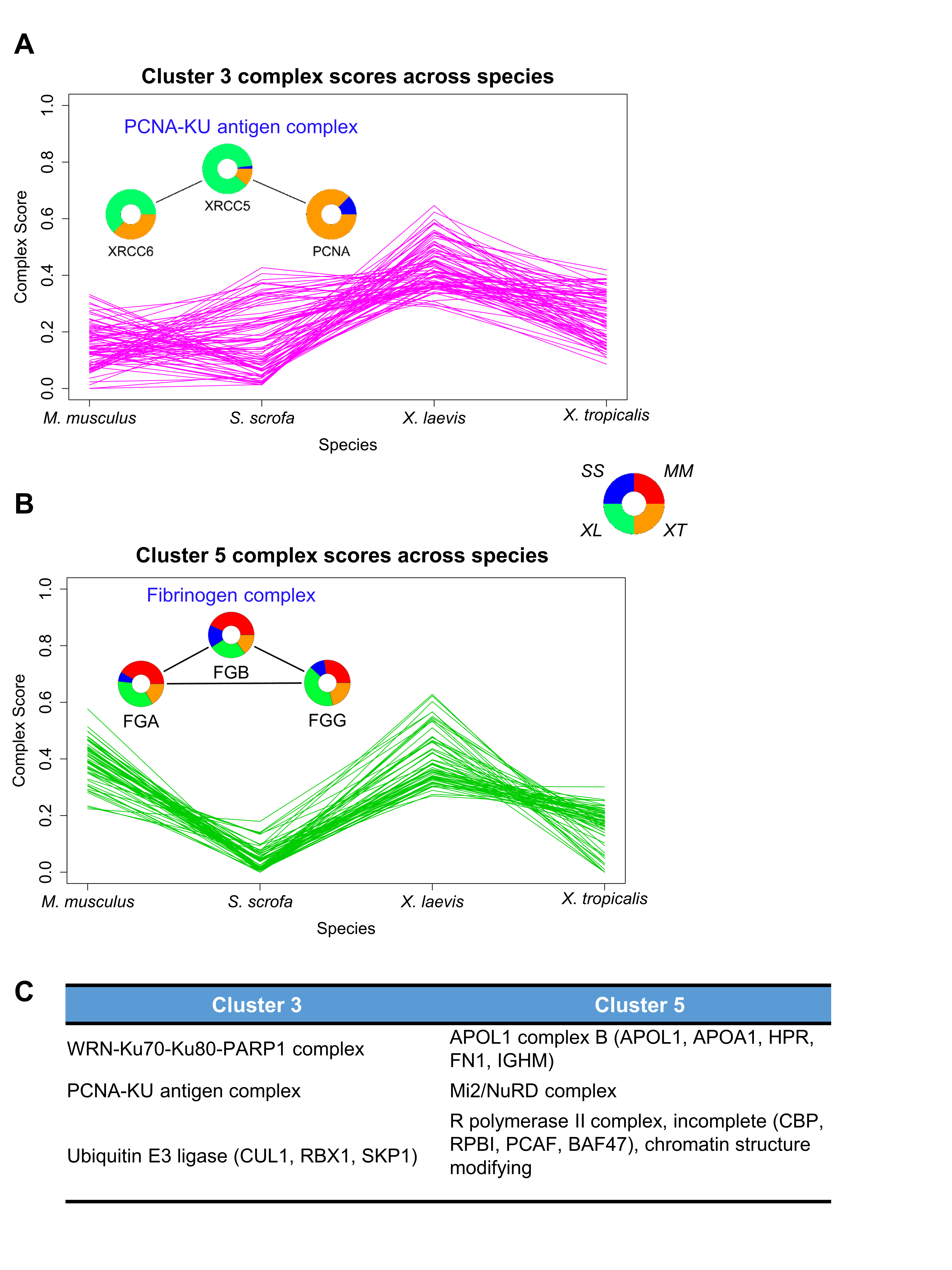

Supplement: S12 Fig — Individual clusters 3 (A) and 5 (B) from the protein complex clustering in Fig 2 are shown and the top 3 complexes from each cluster are listed (C). See S9 Table for numerical data underlying figure. (TIF) [file pbio.3000437.s012.TIF]

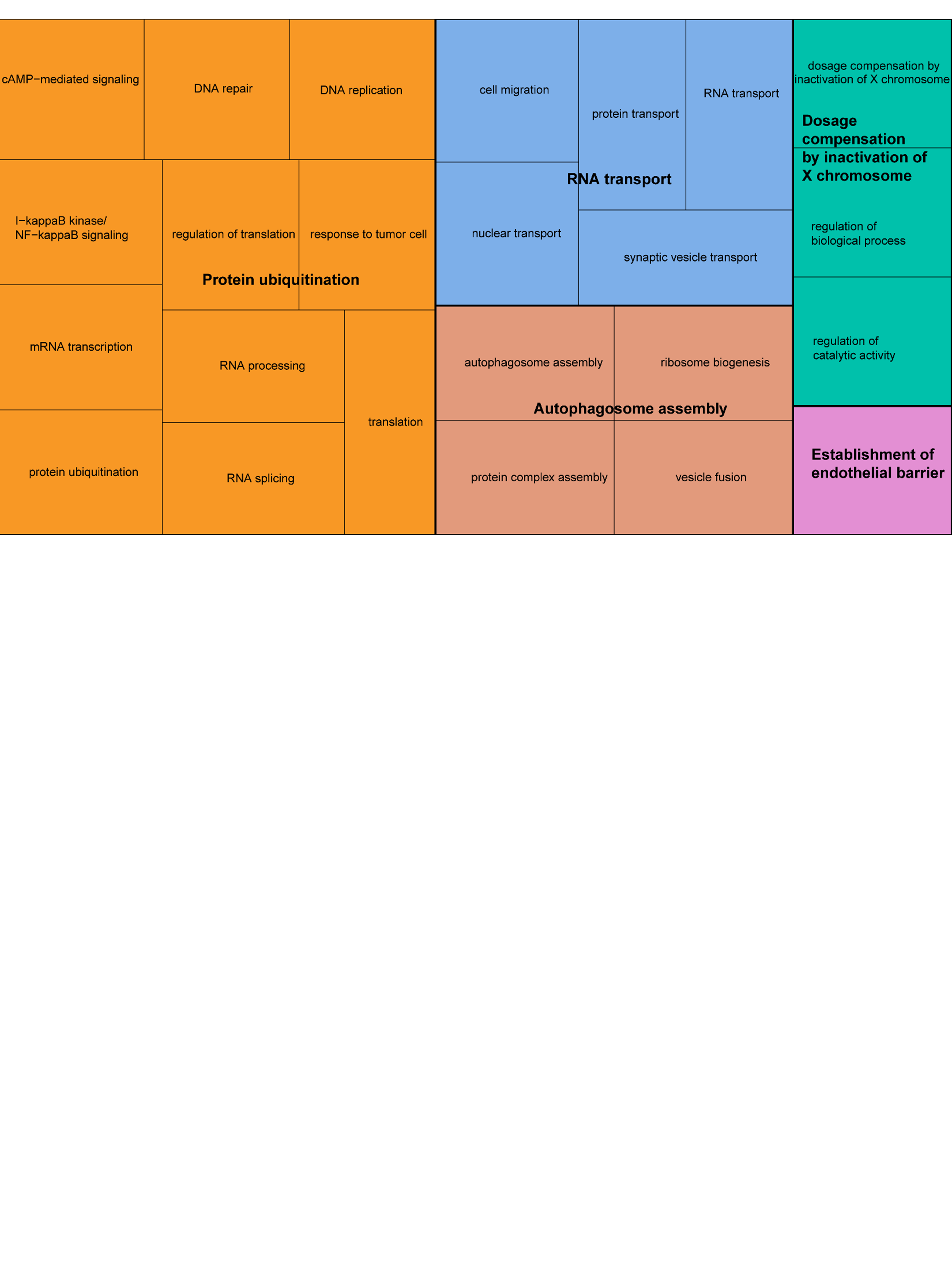

Supplement: S13 Fig — GO, Gene Ontology. (TIF) [file pbio.3000437.s013.TIF]

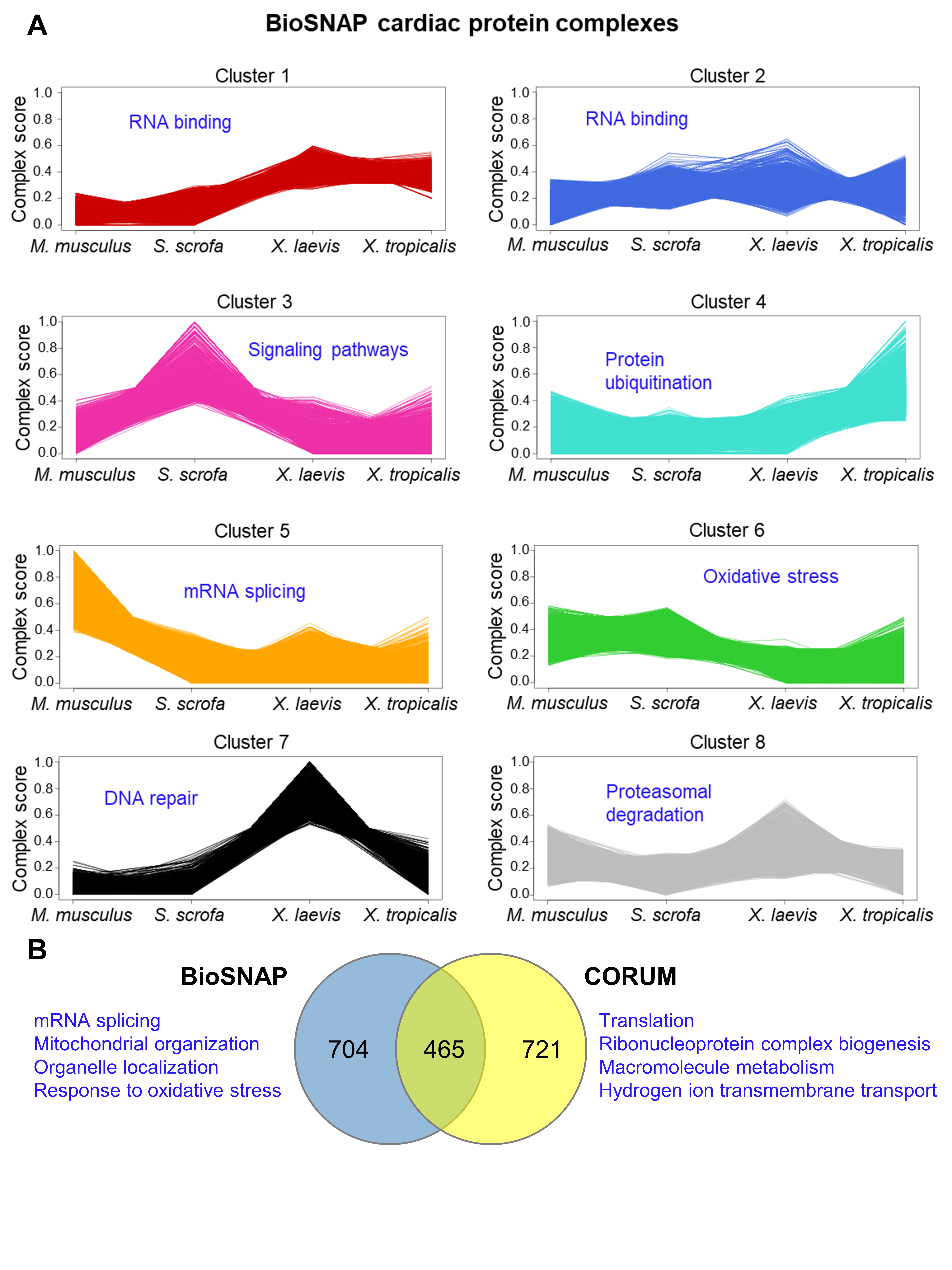

Supplement: S14 Fig — (A) The 8 clusters resulting from mapping the 4 species data set to cardiac complex information in BioSNAP are shown. One of the top GO terms for each cluster is show in blue. (B) Comparison of BioSNAP and CORUM complex analysis. Top enriched GO terms for each unique set of proteins are listed. See S10 Table for numerical data underlying figure. CORUM, Comprehensive Resource of Mammalian Protein Complexes; GO, Gene Ontology. (TIF) [file pbio.3000437.s014.TIF]

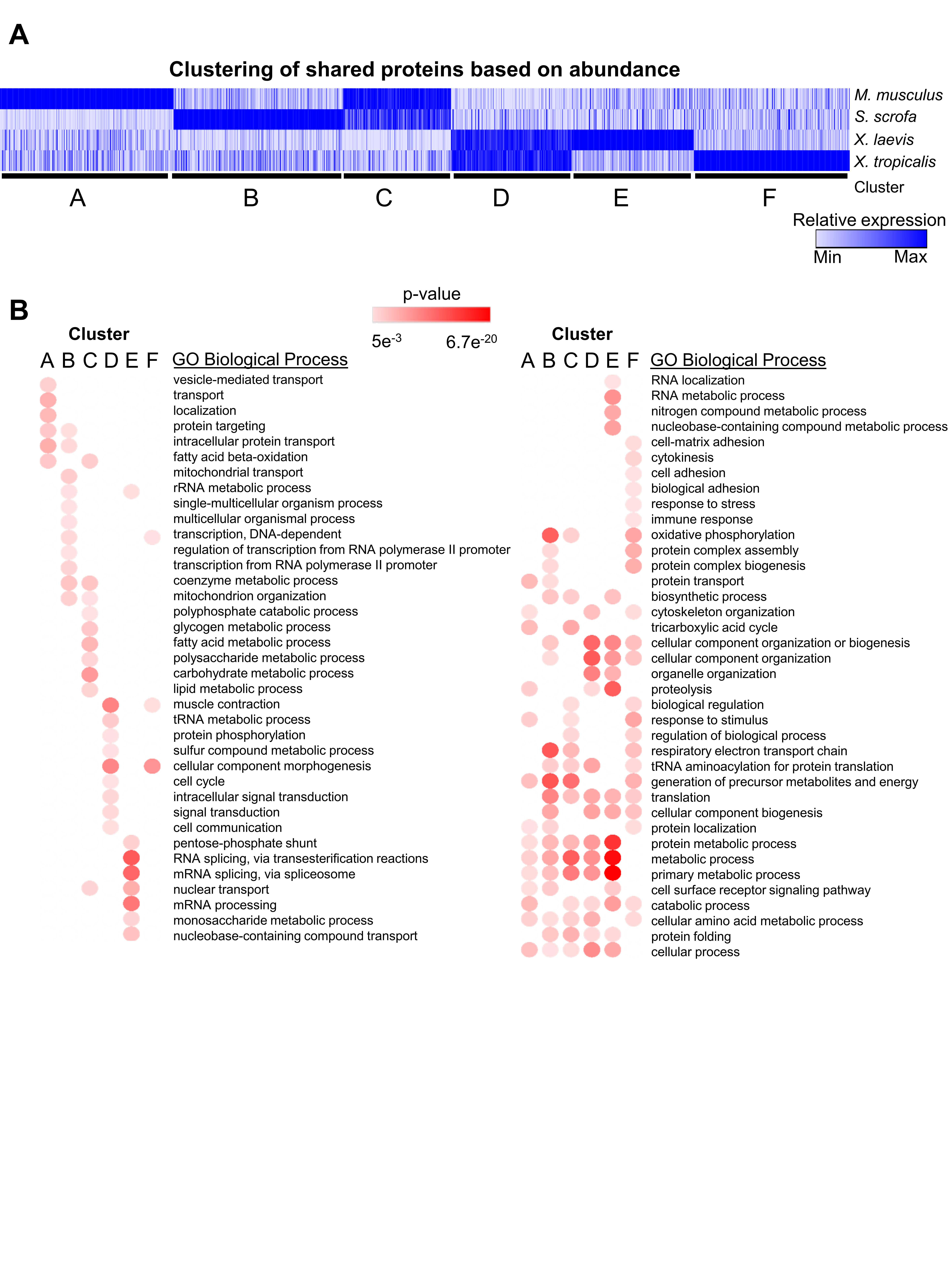

Supplement: S15 Fig — (A) The 1,770 proteins that are shared across all 4 species were clustered using k means with k = 6 revealing clusters driven by higher protein expression in one or more species. (B) Each of the clusters from the heat map were analyzed for overrepresentation of GO biological process terms and the p-values of the significantly enriched terms were shown in a heat map. See S8 Table and S11 Table for numerical data underlying figure. GO, Gene Ontology. (TIF) [file pbio.3000437.s015.TIF]

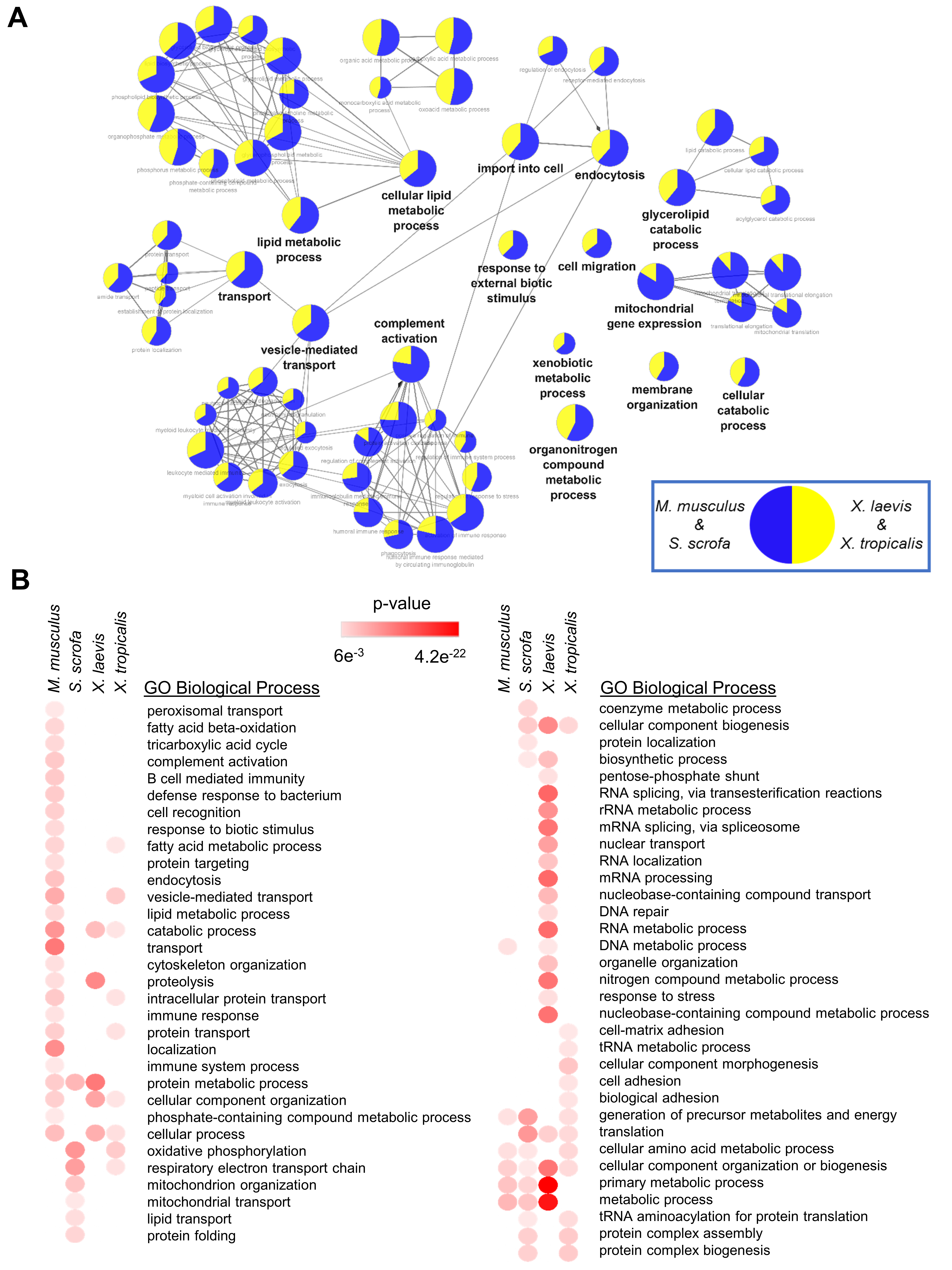

Supplement: S16 Fig — (A) The proteins uniquely identified in the mammalian and Xenopus species were used to examine evolutionary driven protein expression differences in these organisms using ClueGO (B) Proteins found uniquely in one species only were combined with the cluster of proteins that were more highly expressed in that species and analyzed for overrepresentation of GO biological process terms. The p-values of the significantly enriched terms are shown in a heat map. See S12 Table for numerical data underlying figure. GO, Gene Ontology. (TIF) [file pbio.3000437.s016.TIF]

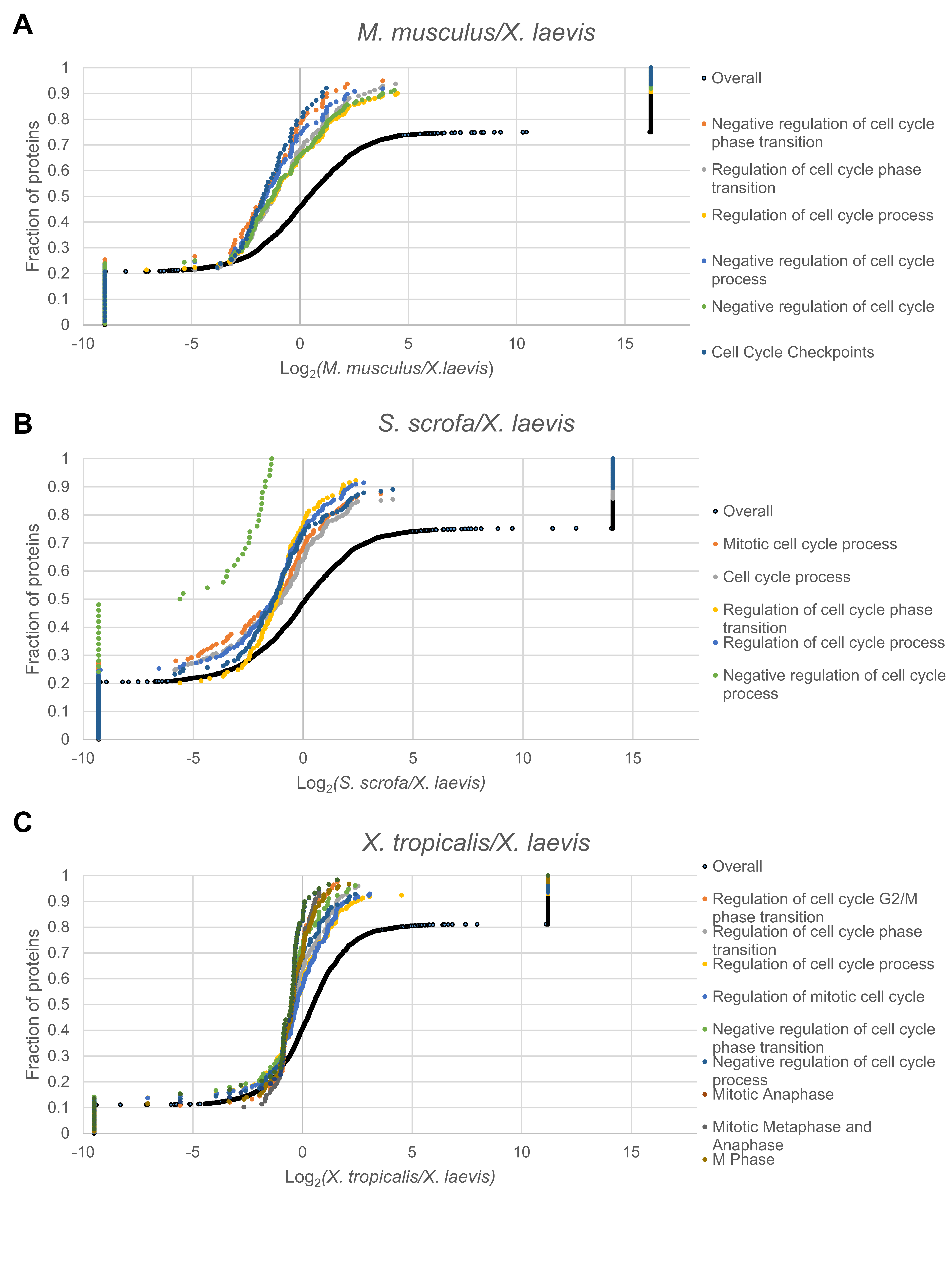

Supplement: S17 Fig — (A) M. musculus/X. laevis; (B) S. scrofa/X. laevis; (C) X. tropicalis/X. laevis. See S13 Table and S14 Table for numerical data underlying figure. GO, Gene Ontology. (TIF) [file pbio.3000437.s017.TIF]

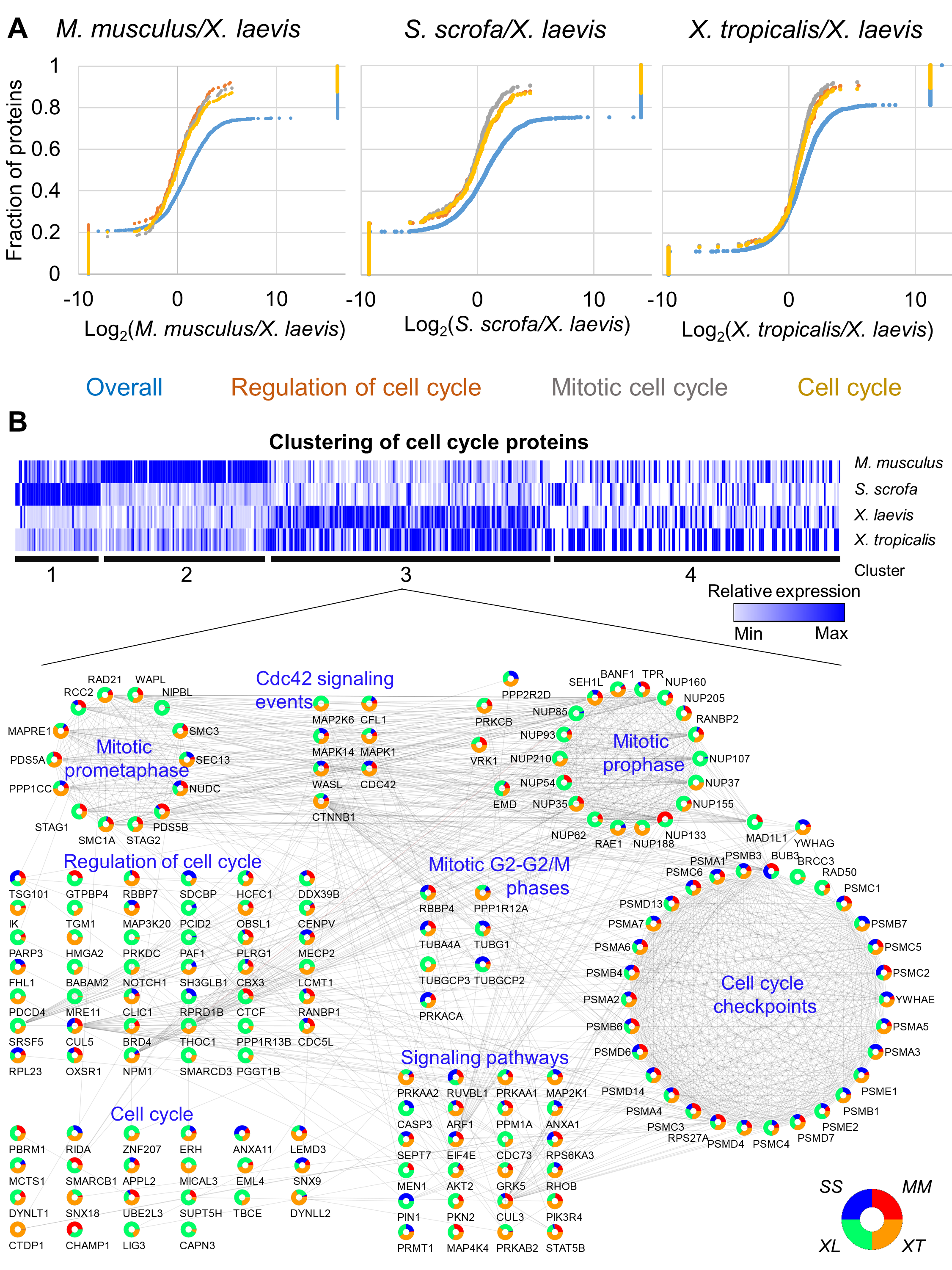

Supplement: S18 Fig — Data from Fig 3 were reproduced here with correction for gene duplication in X. laevis. Similar trends were observed as before. See S15 Table for numerical data underlying figure. (TIF) [file pbio.3000437.s018.TIF]

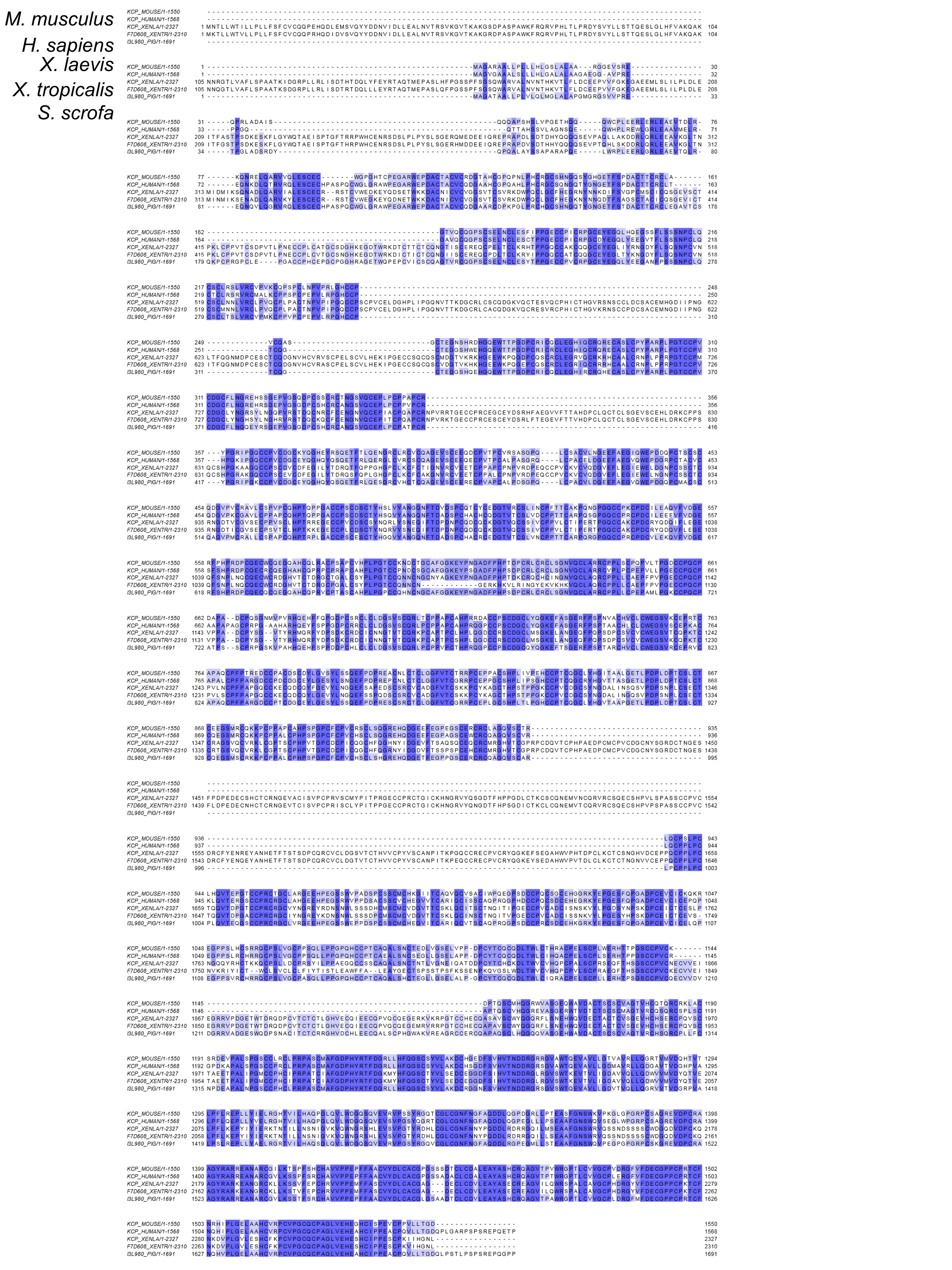

Supplement: S21 Fig — Kcp protein sequences from mouse, human, X. laevis, X. tropicalis, and pig were aligned to examine sequence conservation. Highlighted in dark blue are residues conserved among all 5 species, medium blue shows conservation in 4 species, and light blue depicts sequence conservation in 3 species. Kcp, Kielin/chordin-like protein. (TIF) [file pbio.3000437.s021.TIF]

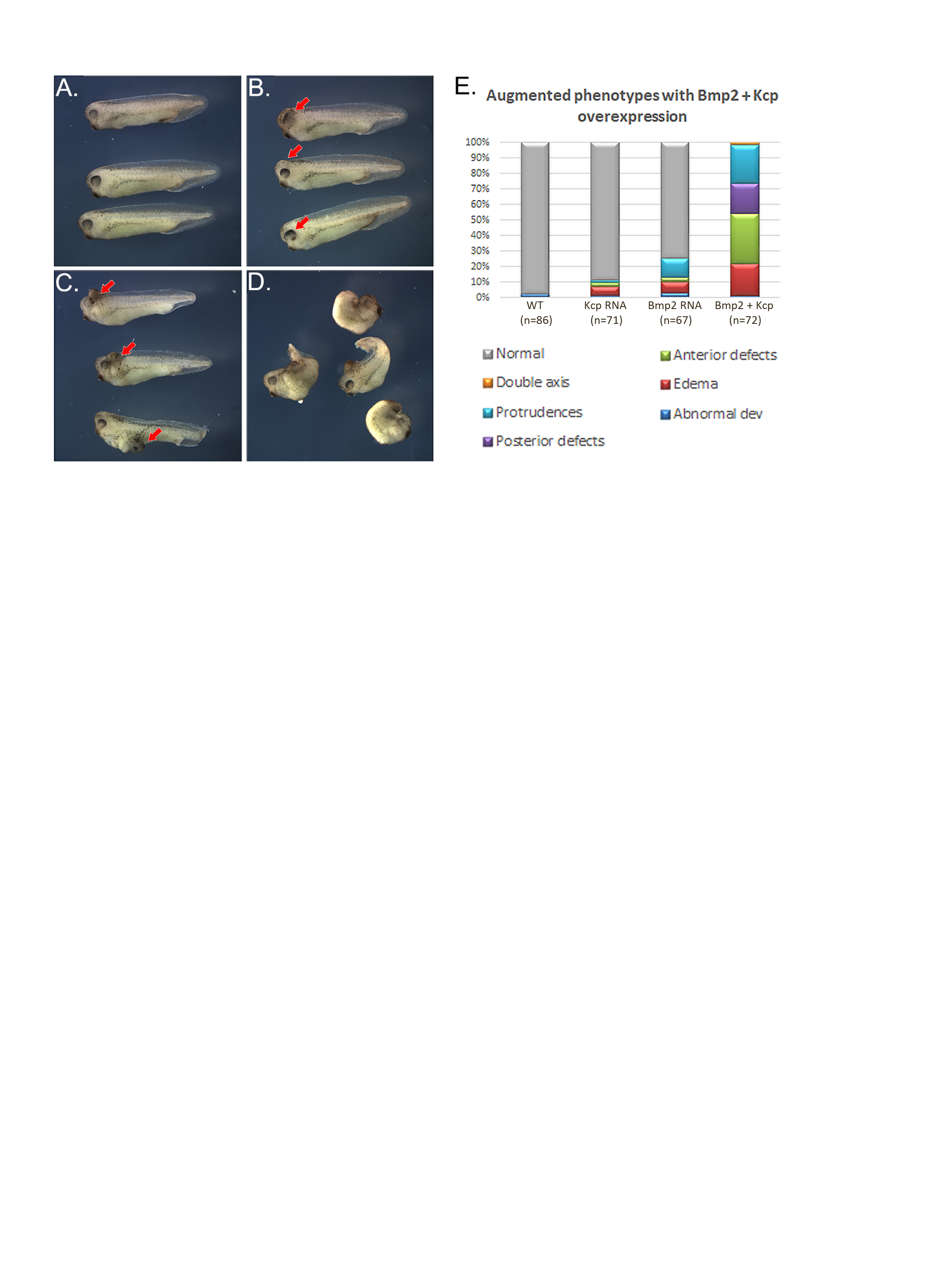

Supplement: S22 Fig — (A) Control embryos; mock injected. (B) Embryos injected with 500 pg Kcp mRNA. Embryos display protrusions (red arrows). (C) Embryos injected with 500 pg Bmp2 mRNA. Embryos displaying partial double axis (red arrows). (D) Embryos injected with 500 pg Kcp mRNA + 500 pg Bmp2 mRNA. Embryos displaying partial double axis, posterior truncations, edema. (E) Graph of the classes of phenotypic abnormalities in wild-type embryos verses those injected with Kcp alone, BMP2 alone, or BMP2 + Kcp. Results from 2 independent biological replicates with total number of embryos scored under each heading. See S17 Table for numerical data underlying figure. BMP, bone morphogenetic protein; Kcp, Kielin/chordin-like protein. (TIF) [file pbio.3000437.s022.tif]
