# Supplementary material for: Conservation and divergence of protein pathways in the vertebrate heart
Source: PLoS Biol. 2019 Sep 6;17(9):e3000437. doi: 10.1371/journal.pbio.3000437 (PMC6750614; doi:10.1371/journal.pbio.3000437)

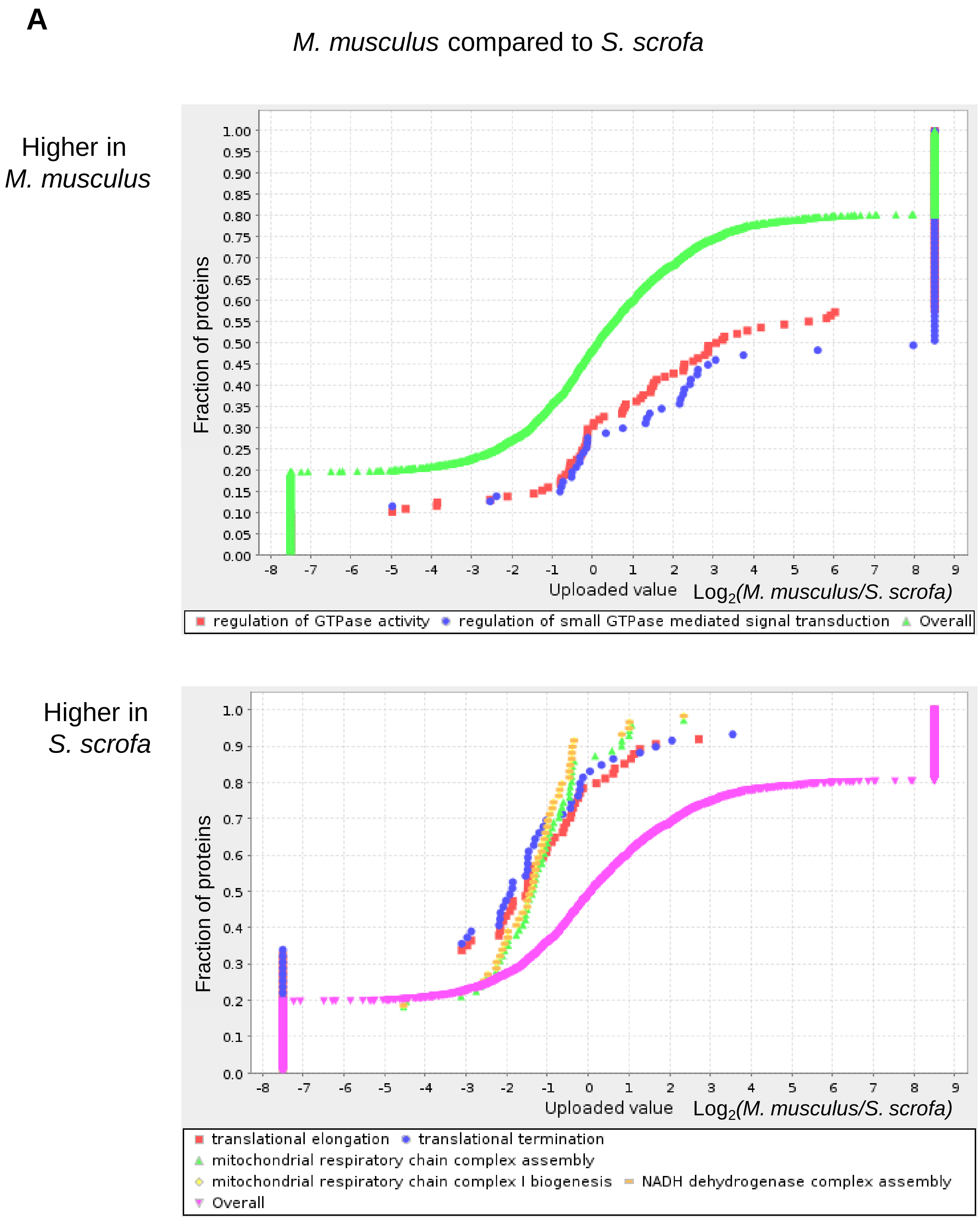

A
M. musculus compared to *S. scrofa*
Higher in *M. musculus*
Fraction of proteins
1.00
0.95
0.90
0.85
0.80
0.75
0.70
0.65
0.60
0.55
0.50
0.45
0.40
0.35
0.30
0.25
0.20
0.15
0.10
0.05
0.00
-8 -7 -6 -5 -4 -3 -2 -1 0 1 2 3 4 5 6 7 8 9
Uploaded value
Log_2(*M. musculus/S. scrofa*)
regulation of GTPase activity
regulation of small GTPase mediated signal transduction
Overall
Higher in *S. scrofa*
Fraction of proteins
1.0
0.9
0.8
0.7
0.6
0.5
0.4
0.3
0.2
0.1
0.0
-8 -7 -6 -5 -4 -3 -2 -1 0 1 2 3 4 5 6 7 8 9
Uploaded value
Log_2(*M. musculus/S. scrofa*)
translational elongation
translational termination
mitochondrial respiratory chain complex assembly
mitochondrial respiratory chain complex I biogenesis
NADH dehydrogenase complex assembly
Overall

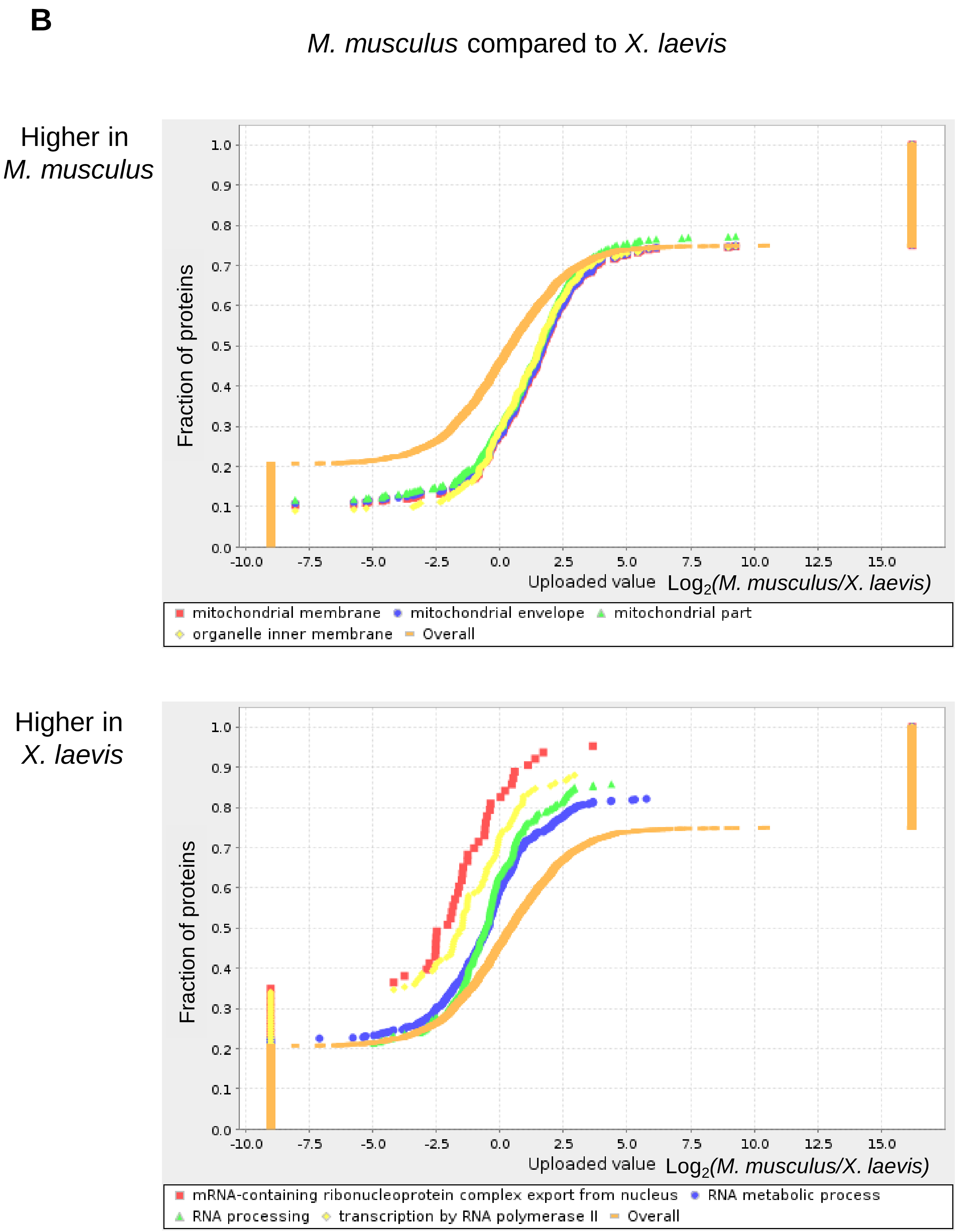
B
M. musculus compared to X. laevis
Higher in M. musculus
Fraction of proteins
1.0
0.9
0.8
0.7
0.6
0.5
0.4
0.3
0.2
0.1
0.0
-10.0
-7.5
-5.0
-2.5
0.0
2.5
5.0
7.5
10.0
12.5
15.0
Uploaded value
Log2(M. musculus/X. laevis)
mitochondrial membrane
mitochondrial envelope
mitochondrial part
organelle inner membrane
Overall
Higher in X. laevis
Fraction of proteins
1.0
0.9
0.8
0.7
0.6
0.5
0.4
0.3
0.2
0.1
0.0
-10.0
-7.5
-5.0
-2.5
0.0
2.5
5.0
7.5
10.0
12.5
15.0
Uploaded value
Log2(M. musculus/X. laevis)
mRNA-containing ribonucleoprotein complex export from nucleus
RNA metabolic process
RNA processing
transcription by RNA polymerase II
Overall

C

M. musculus compared to *X. tropicalis*

Higher in
M. musculus

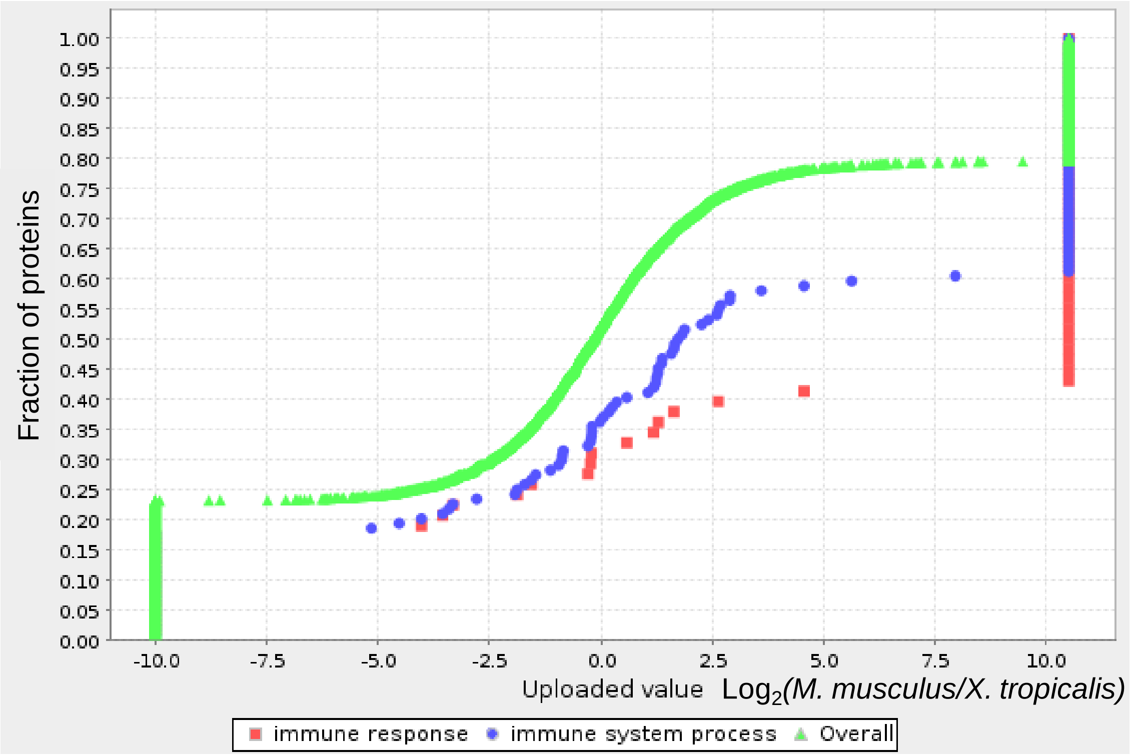

Higher in
M. musculus

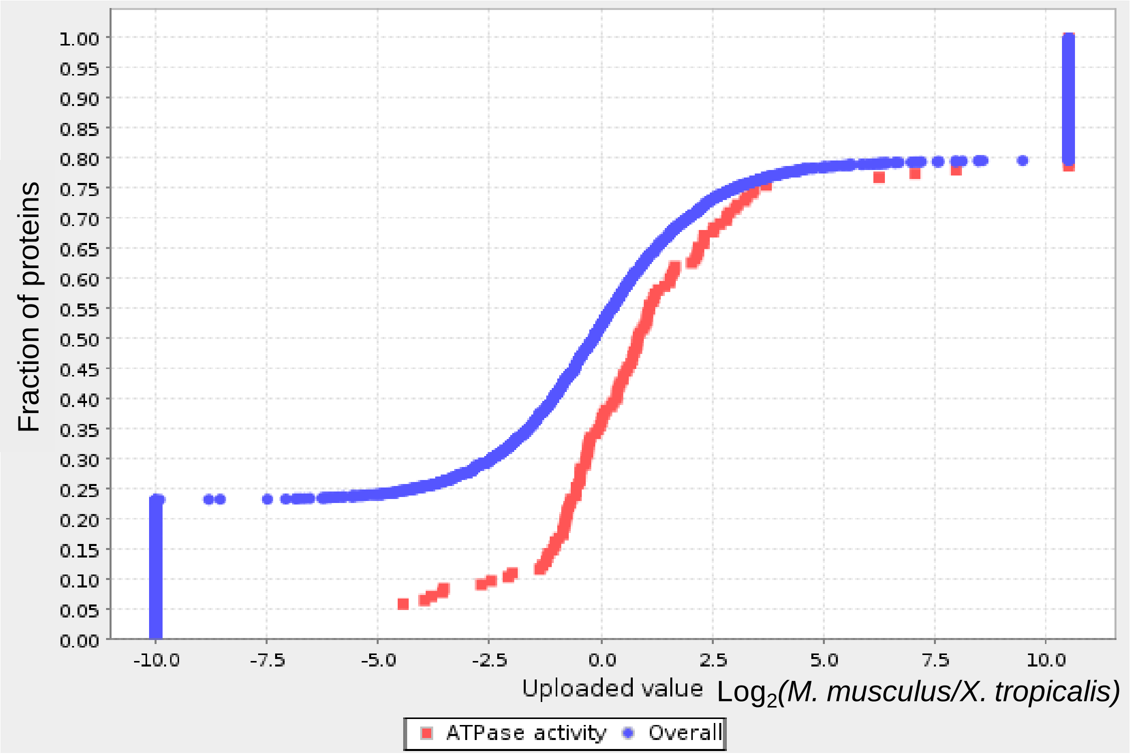

D

S. scrofa compared to *X. laevis*

Higher in *S. scrofa*

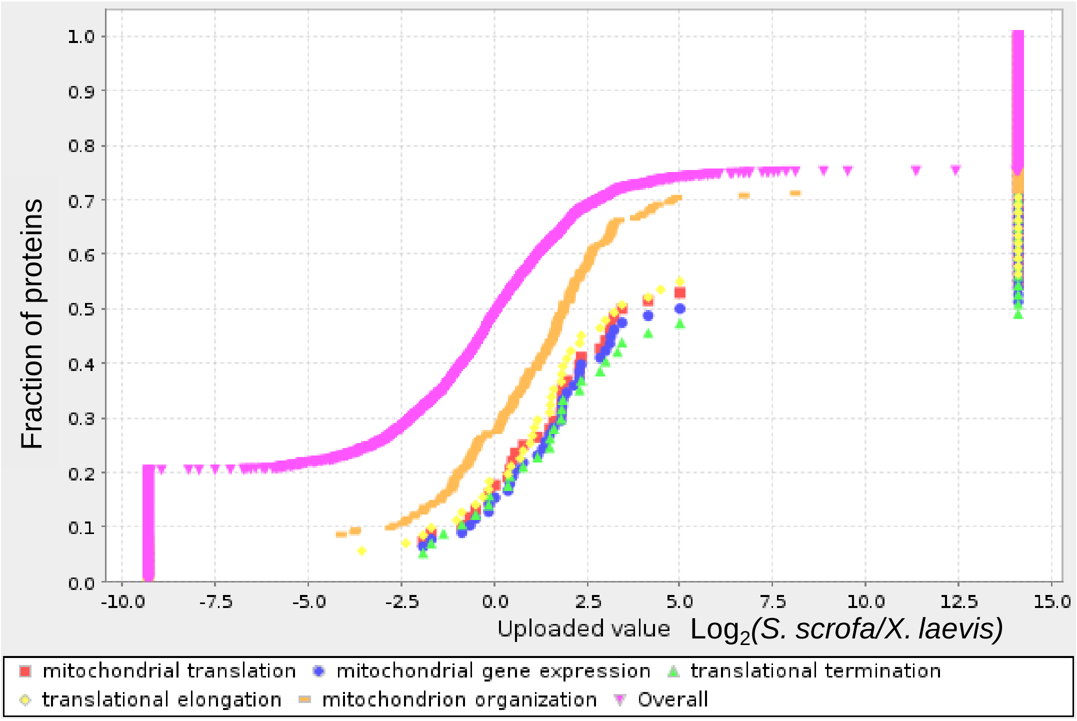

Higher in *X. laevis*

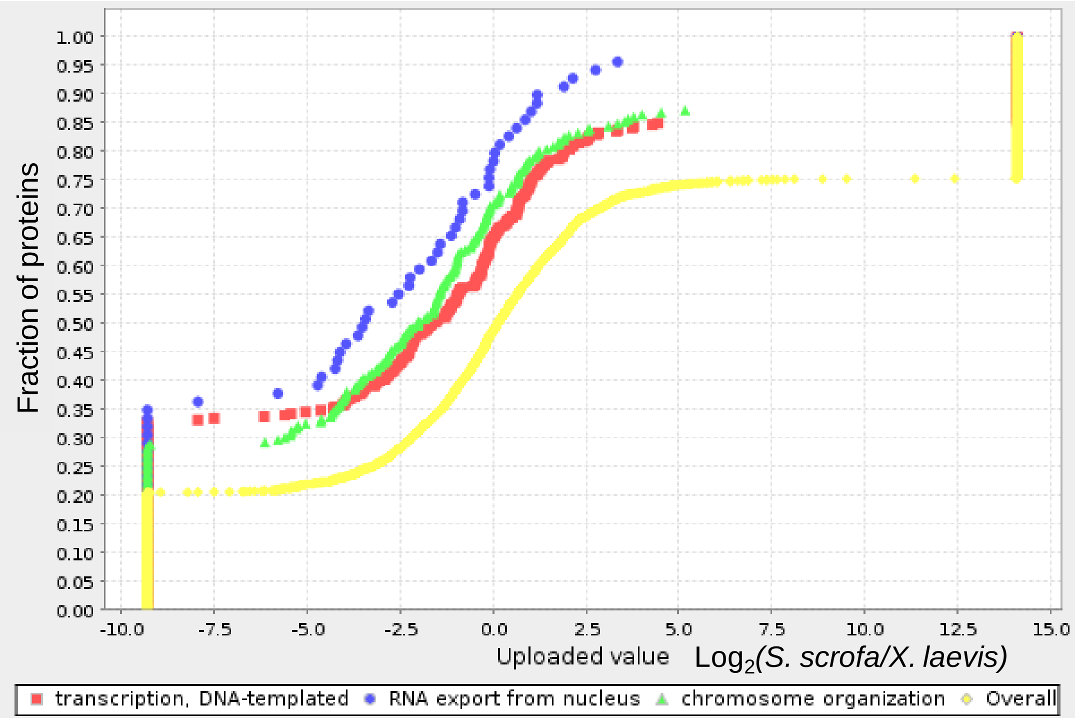

E

S. scrofa compared to *X. tropicalis*

Higher in *S. scrofa*

Higher in *X. tropicalis*

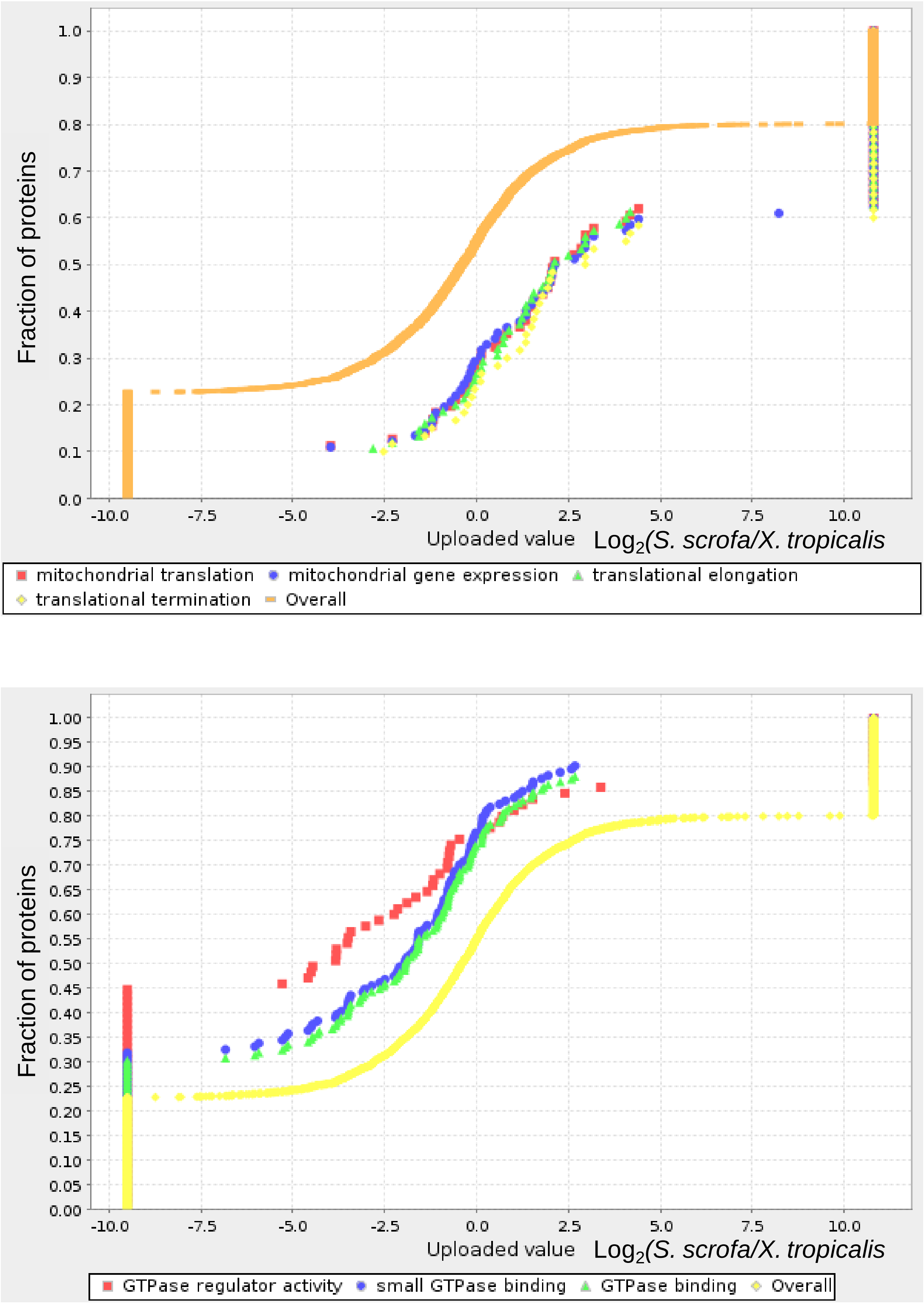

F

X. tropicalis compared to *X. laevis*

Higher in
X. tropicalis

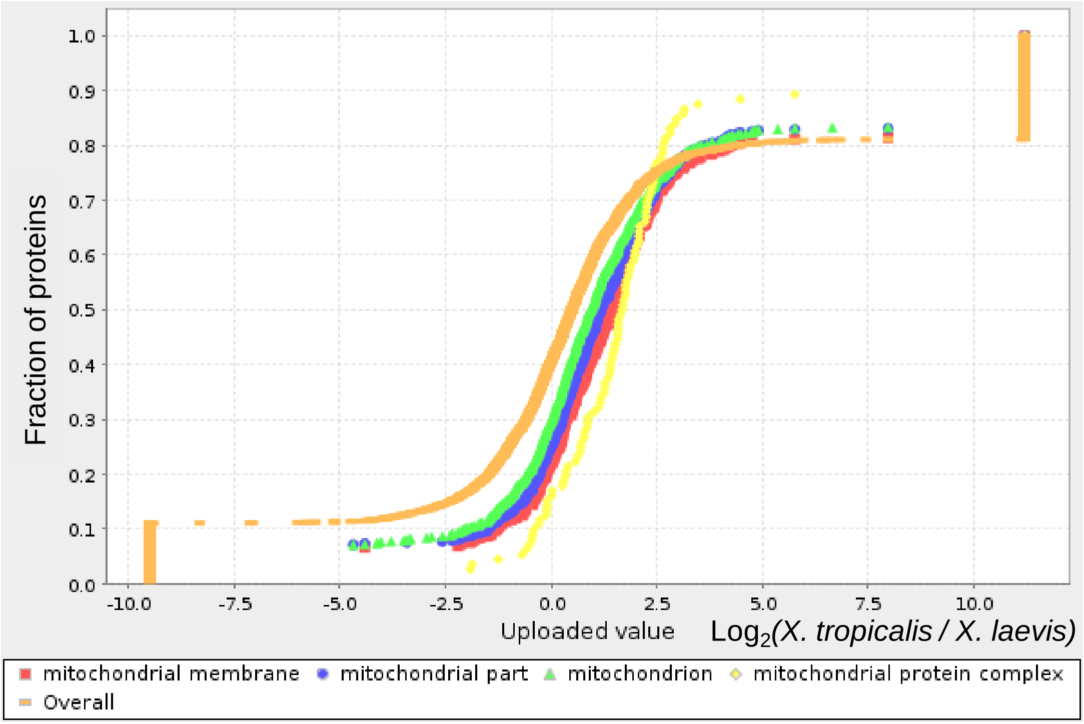

Higher in
X. laevis

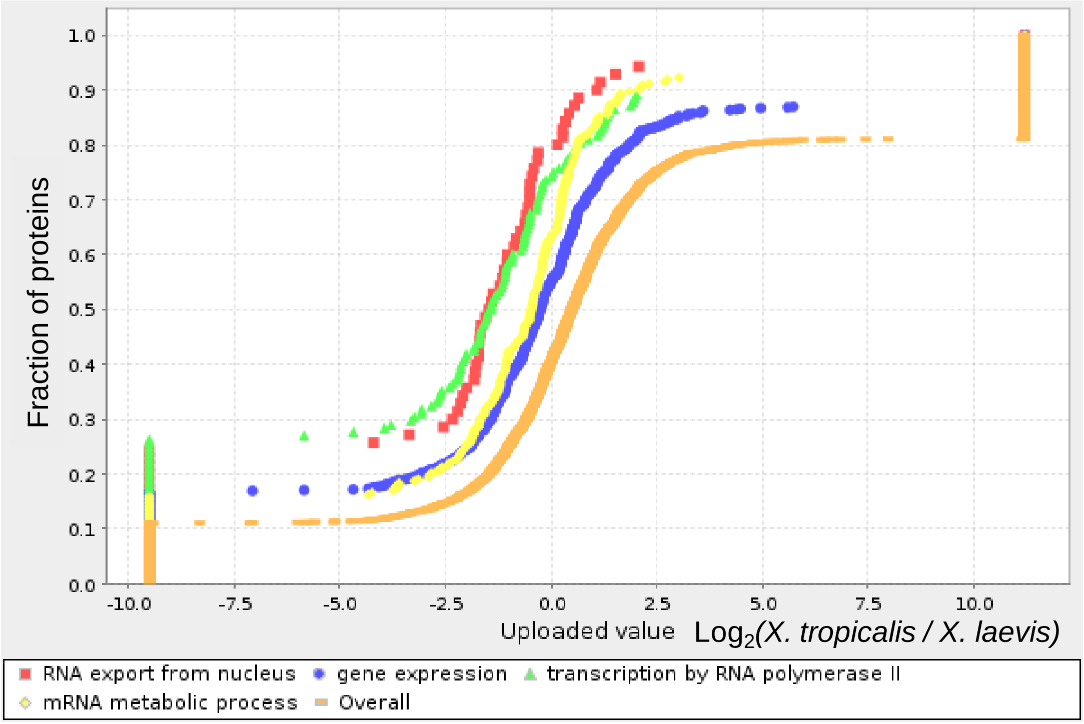

Supplement: S19 Fig — (A) M. musculus/S. scrofa; (B) M. musculus/X. laevis; (C) M. musculus/X. tropicalis; (D) S. scrofa/X. laevis; (E) S. scrofa/X. tropicalis; (F) X. laevis/X. tropicalis. See S13 Table and S14 Table for numerical data underlying figure. GSEA, Gene Set Enrichment Analyses. (PDF) [file pbio.3000437.s019.pdf]
