# Supplementary material for: Conservation and divergence of protein pathways in the vertebrate heart
Source: PLoS Biol. 2019 Sep 6;17(9):e3000437. doi: 10.1371/journal.pbio.3000437 (PMC6750614; doi:10.1371/journal.pbio.3000437)
Supplement: S20 Fig — Gene names are listed above the graphs. Proteins for which X. laevis was significantly higher than the other species are noted with an asterisk. (*p ≤ 0.05, **p ≤ 0.01, ***p ≤ 0.001, ****p ≤ 0.0001). See S16 Table for numerical data underlying figure. PRM, parallel reaction monitoring. (PDF) [file pbio.3000437.s020.pdf]

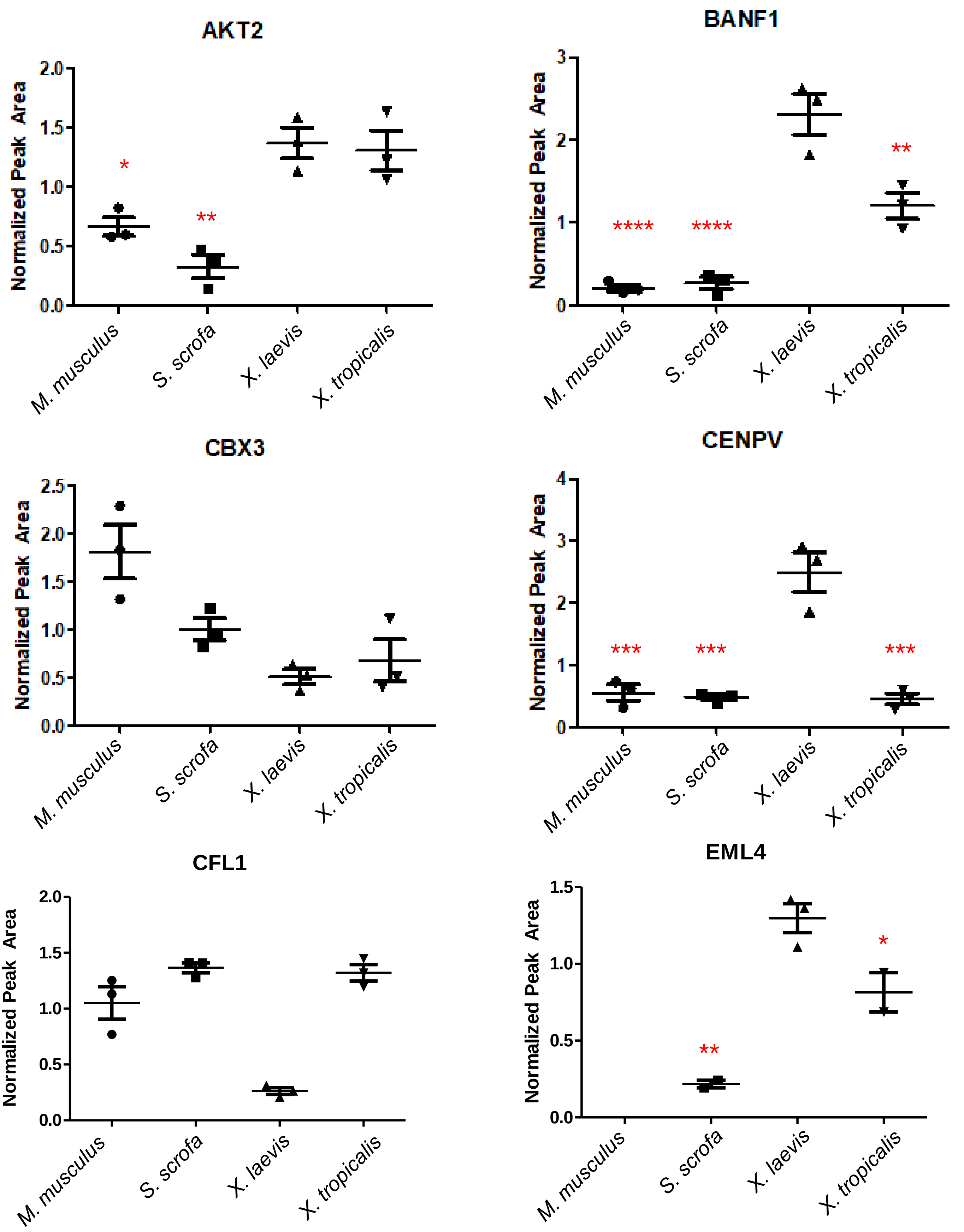

AKT2
Normalized Peak Area
2.0
1.5
1.0
0.5
0.0
*
**
M. musculus
S. scrofa
X. laevis
X. tropicalis
BANF1
Normalized Peak Area
3
2
1
0
****
****
**
M. musculus
S. scrofa
X. laevis
X. tropicalis
CBX3
Normalized Peak Area
2.5
2.0
1.5
1.0
0.5
0.0
M. musculus
S. scrofa
X. laevis
X. tropicalis
CENPV
Normalized Peak Area
4
3
2
1
0
***
***
***
M. musculus
S. scrofa
X. laevis
X. tropicalis
CFL1
Normalized Peak Area
2.0
1.5
1.0
0.5
0.0
M. musculus
S. scrofa
X. laevis
X. tropicalis
EML4
Normalized Peak Area
1.5
1.0
0.5
0.0
**
*
M. musculus
S. scrofa
X. laevis
X. tropicalis

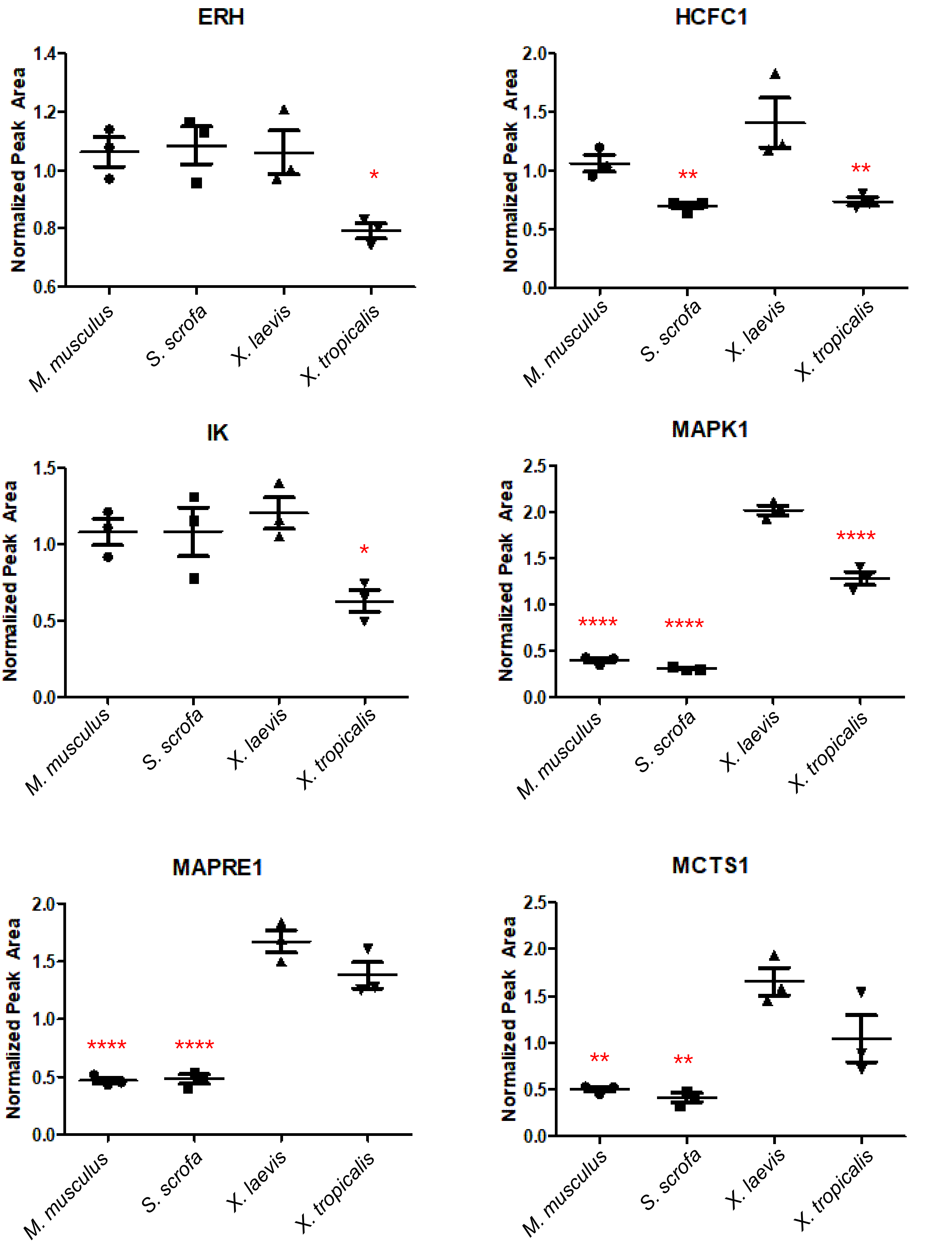

ERH
HCFC1
IK
MAPK1
MAPRE1
MCTS1
Normalized Peak Area
M. musculus
S. scrofa
X. laevis
X. tropicalis
*
**
****

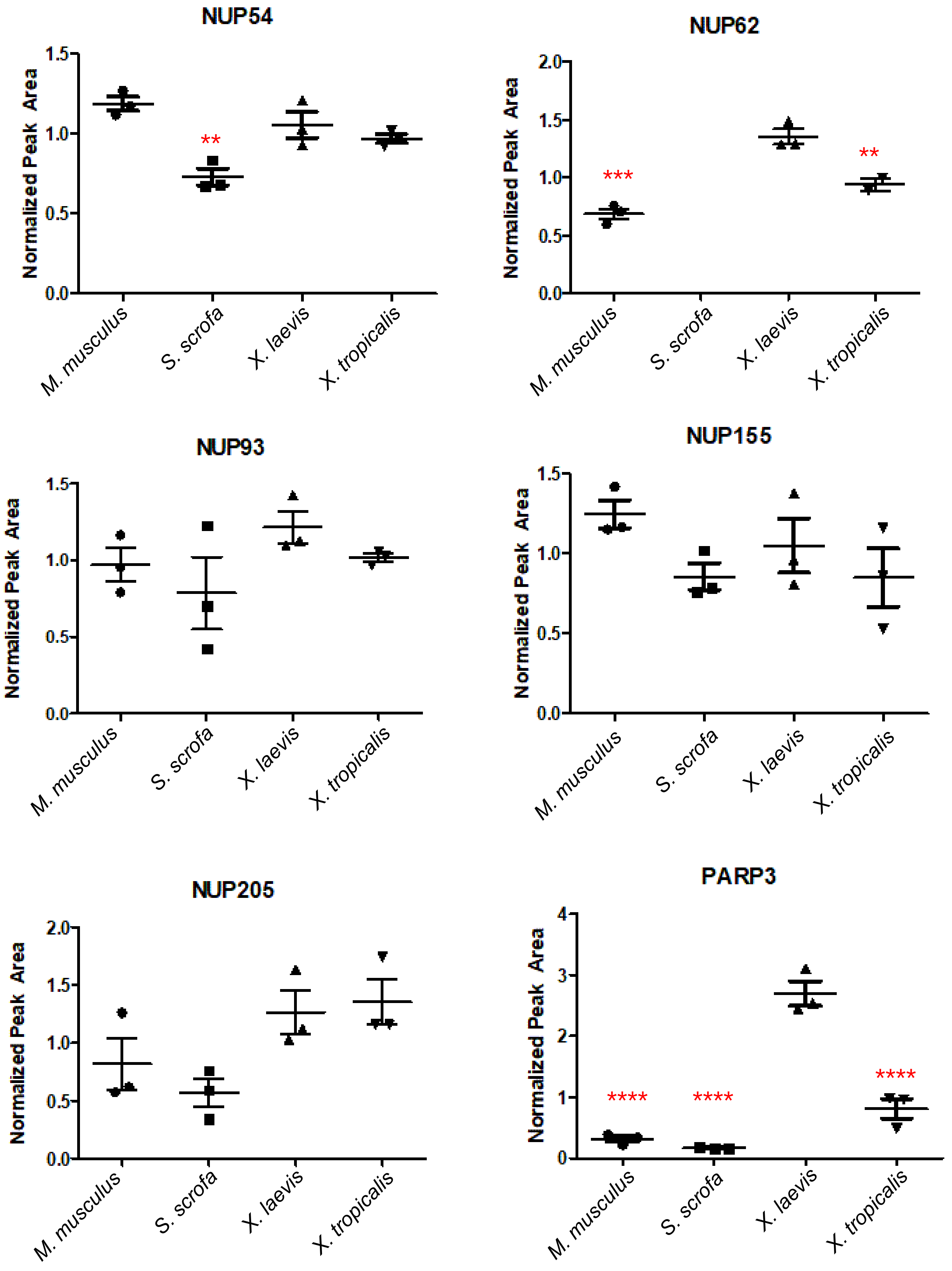
NUP54
NUP62
NUP93
NUP155
NUP205
PARP3
Normalized Peak Area
M. musculus
S. scrofa
X. laevis
X. tropicalis
**
***
**
****
****
****

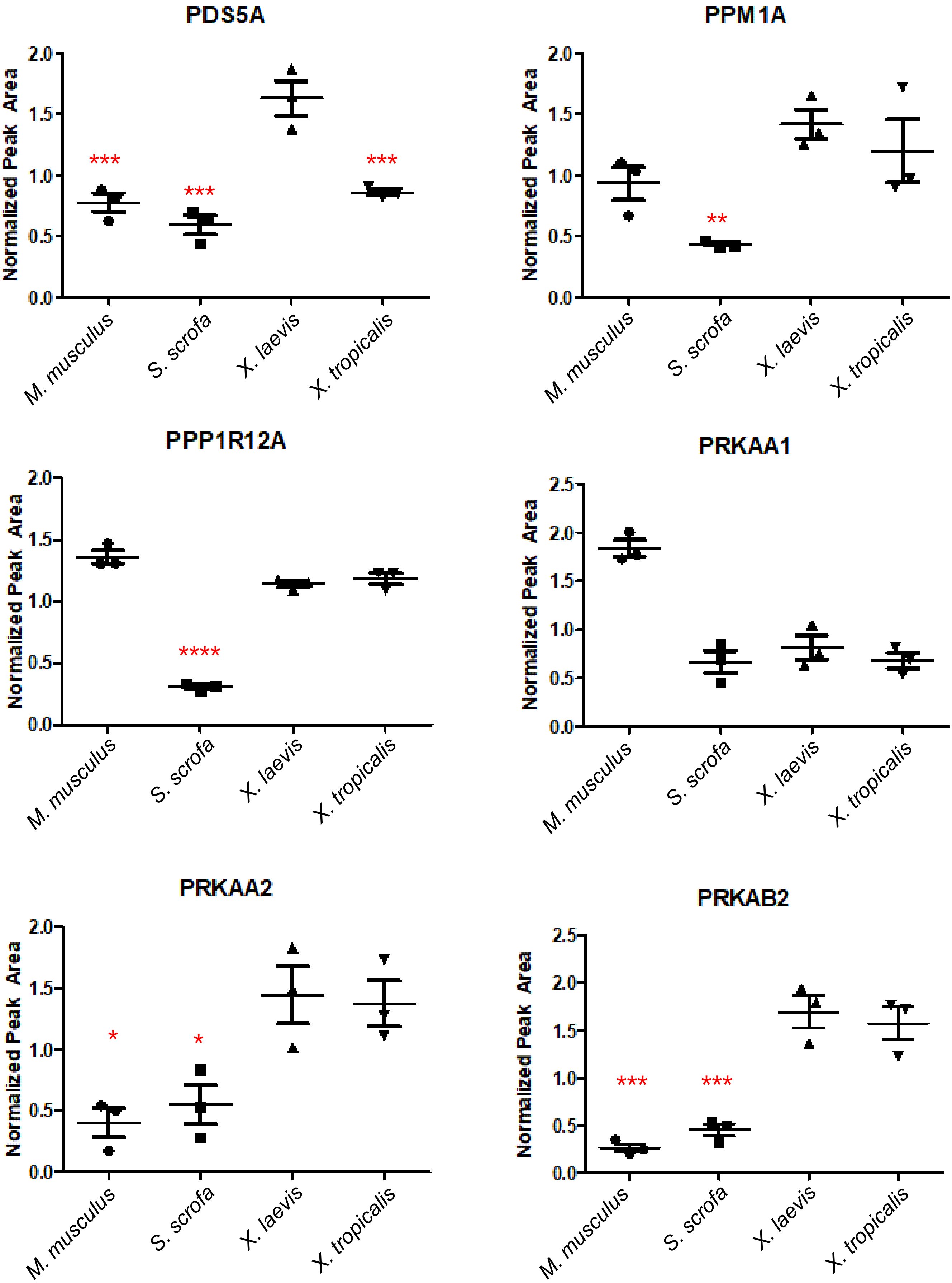
PDS5A
PPM1A
PPP1R12A
PRKAA1
PRKAA2
PRKAB2
Normalized Peak Area
0.0
0.5
1.0
1.5
2.0
2.5
M. musculus
S. scrofa
X. laevis
X. tropicalis
***
**
****
*

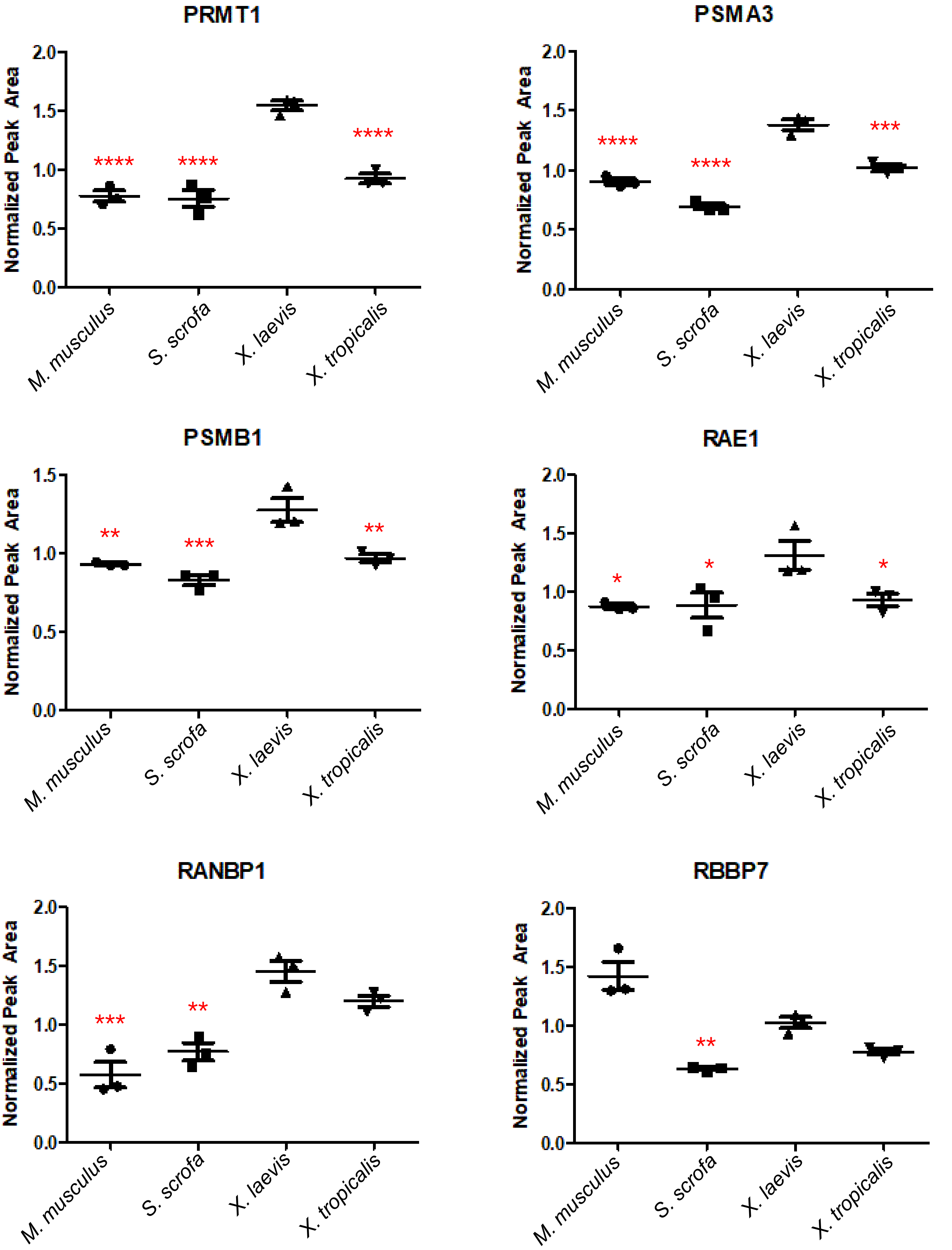
PRMT1
PSMA3
PSMB1
RAE1
RANBP1
RBBP7
Normalized Peak Area
2.0
1.5
1.0
0.5
0.0
M. musculus
S. scrofa
X. laevis
X. tropicalis
****
***
**
*

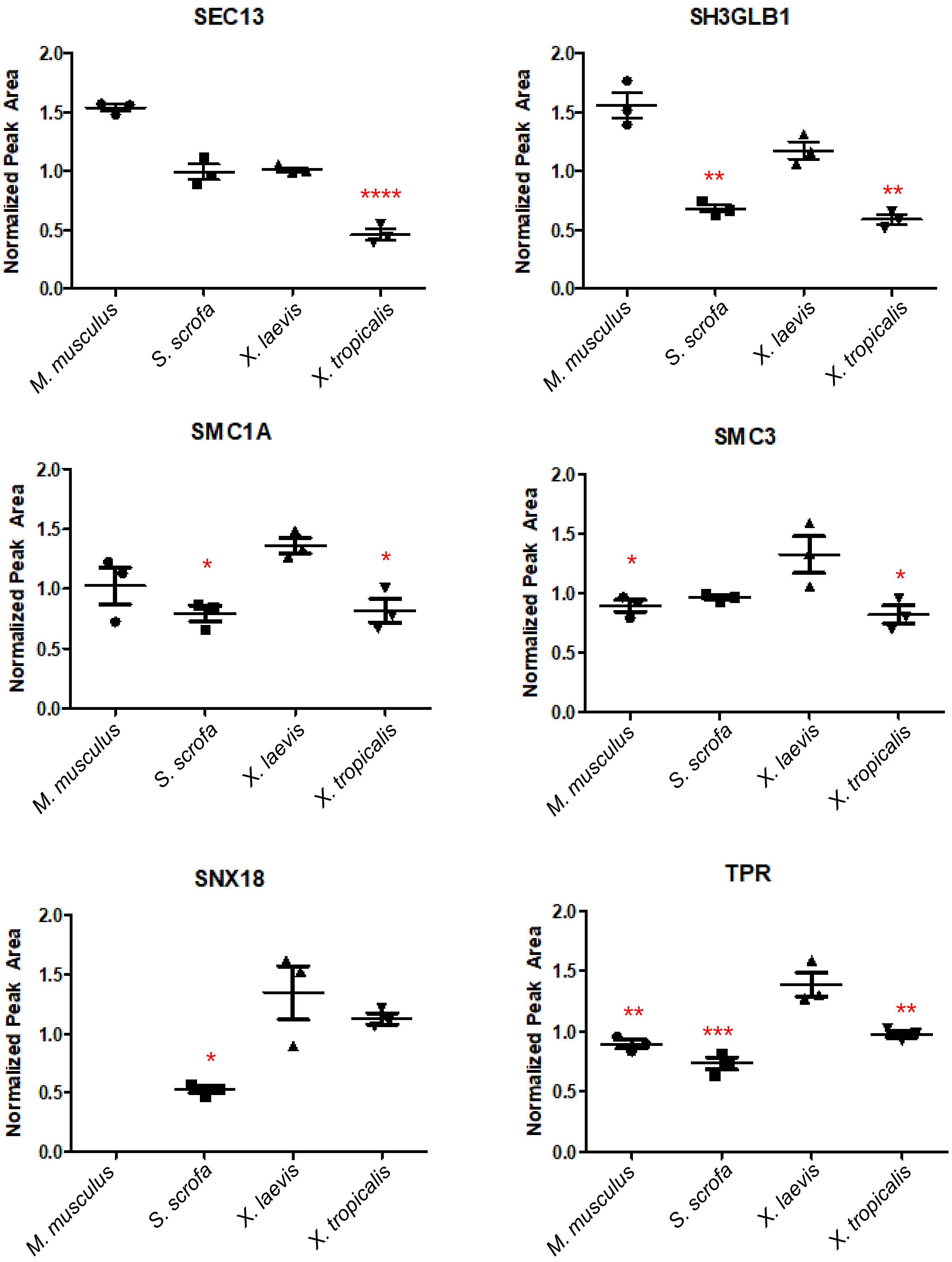
SEC13
SH3GLB1
SMC1A
SMC3
SNX18
TPR
Normalized Peak Area
2.0
1.5
1.0
0.5
0.0
M. musculus
S. scrofa
X. laevis
X. tropicalis
****
**
*

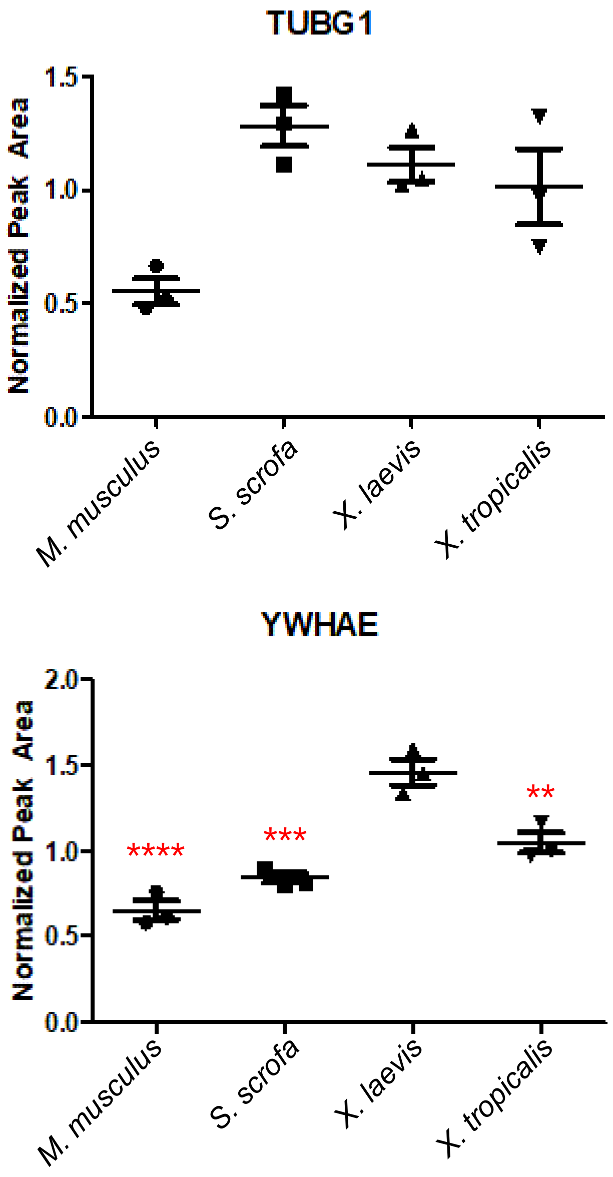

TUBG1
UBE2L3
YWHAE
Normalized Peak Area
1.5
1.0
0.5
0.0
2.0
M. musculus
S. scrofa
X. laevis
X. tropicalis
**
*
****
***
**
